# Epstein‐Barr Virus Expressed Long Non‐Coding RNA (*lncBART*s) Regulate EBV Latent Genome Replication

**DOI:** 10.1002/advs.202507286

**Published:** 2025-11-11

**Authors:** Jiayan Liu, Dittman Lai‐Shun Chung, Larry Ka‐Yue Chow, Shuo Han, Wenkai Yi, Conor J. Cremin, Yingyin Liao, Pui Wang, Bobo Wing‐Yee Mok, Mingfang Ji, Jian Yan, Wei Dai, Honglin Chen

**Affiliations:** ^1^ State Key Laboratory for Emerging Infectious Diseases and Department of Microbiology The University of Hong Kong Pokfulam Hong Kong SAR China; ^2^ Centre for Virology Vaccinology and Therapeutics Limited Hong Kong SAR China; ^3^ Department of Clinical Oncology The University of Hong Kong Pokfulam Hong Kong SAR China; ^4^ School of Biomedical Sciences Li Ka Shing Faculty of Medicine The University of Hong Kong, Pokfulam Hong Kong SAR China; ^5^ Department of Biomedical Sciences City University of Hong Kong Hong Kong SAR China; ^6^ Cancer Research Institute of Zhongshan City Zhongshan People's Hospital Zhongshan Guangdong China

**Keywords:** BRD4, EBV copy number, EBV *LncBART*s, oncogenesis

## Abstract

Epstein‐Barr virus (EBV) is a ubiquitous human virus that is also linked to various human cancers. In EBV‐associated nasopharyngeal carcinoma and gastric carcinoma cells, EBV expresses only essential viral antigens but high levels of long non‐coding RNAs known as BamHI‐A rightward transcripts (*lncBART*s). The exact roles *lncBART*s in the EBV life cycle and the development of EBV‐associated cancers are largely not understood. This study demonstrates that *lncBART*s play a role in maintaining viral genome replication by affecting the tethering of EBV *ori*P region to chromosome. Mechanistically, *lncBART*s interact functionally with a complex consisting of Bromodomain‐containing protein 4 (BRD4), CCTC‐binding factor (CTCF), and EBV nuclear antigen 1 (EBNA1), anchoring their binding at *ori*P to facilitate EBV episome replication. Additionally, the *lncBART*s‐BRD4/CTCF complex contributes to the regulation of *MYC* and *BCL2* expression. These findings suggest that *lncBART*s modulate EBV latency through interactions with *ori*P region, while *lncBART*s‐BRD4/CTCF complexes promote host epigenome reprogramming and drive tumorigenesis in EBV‐associated epithelial tumors.

## Graphical abstract

EBV produces abundant level of *lncBART*s, which are essential for maintaining viral genome replication in EBV‐associated cancers. *LncBARTs* interact with a complex comprising BRD4, CTCF and viral protein EBNA1 at EBV *oriP* region. This interaction tethers *oriP* to host chromosomes, facilitating EBV episome replication. Additionally, *lncBART*s‐BRD4 complex alters the expression of proto‐oncogenes including *MYC* and *BCL2* to drive tumorigenesis.

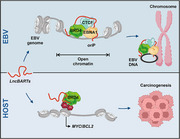

## 1 Introduction

Epstein‐Barr virus (EBV), the first confirmed oncogenic virus, infects ≈95% of the world's population.^[^ ^1^ ^]^ EBV is strongly linked to the development of certain tumors affecting lymphoid or epithelial tissues, such as Burkitt lymphoma, Hodgkin lymphoma, nasopharyngeal carcinoma (NPC) and EBV‐associated gastric cancer (EBVaGC).^[^ ^2^, ^3^, ^4^ ^]^ Undifferentiated NPC exhibits the strongest association with EBV infection.^[^ ^5^, ^6^, ^7^ ^]^ To circumvent the host immune system and establish a conducive intracellular environment for its survival, EBV institutes latent infections in tumor cells, expressing only minimal amounts of viral proteins.^[^ ^8^, ^9^ ^]^ Interestingly, increased levels of RNAs are transcribed from the EBV BamHI‐A rightward transcript (BART) region. First referred to as complementary strand transcripts (CSTs), this family of transcripts include microRNAs originating from introns, EBV circRNAs transcribed from exon‐intron regions and long non‐coding BARTs (*lncBART*s) derived from multiple exons.^[^ ^10^, ^11^, ^12^, ^13^ ^]^ Multiple alternative spliced isoforms of BARTs have been identified but all BART transcripts are 3′ end coterminal.^[^ ^14^ ^]^ As the major isoform of *lncBART*s, *RPMS1* is recognized as a full length transcript that contains all exons across the entire BART region.^[^ ^11^, ^15^ ^]^ Early attempts to identify proteins expressed from putative open reading frames (ORFs) of BARTs were not successful,^[^ ^16^ ^]^ and subsequent research indicates that BARTs function as a class of lncRNAs expressed by EBV.^[^ ^17^, ^18^ ^]^ *LncBART*s were found to localize predominantly in the nucleus and overexpression of a BART transcript isoform in EBV‐negative AGS cells shown to alter gene transcription.^[^ ^17^, ^19^ ^]^ Recent research has shown the significance of EBV non‐coding RNAs in contributing to the tumor phenotype through promoting cell cycle progression, suppressing both innate and adaptive immune responses, and inhibiting apoptosis.^[^ ^18^, ^20^, ^21^ ^]^ Our previous research showed that *lncBART*s interact with the CBP/p300 complex and RNA Pol II in the nucleus, implying that *lncBART*s regulate gene expression through an epigenetic mechanism.^[^ ^18^, ^22^ ^]^ Despite these promising finding related to *lncBART*s, our understanding of their precise functional mechanism in EBV life cycle and tumorigenesis remains limited.

LncRNAs have emerged as key regulators in cancer development and progression. Similarly, viruses produce lncRNAs to enhance viral survival and pathogenicity. Using non‐coding RNAs to manipulate host cell signalling pathways is a viral tactic for shaping the host cell environment to meet the virus's requirements without triggering immune elimination and can serve a key role in the host‐virus interaction. For instance, the latency‐associated transcript (*LAT*), the only viral gene expressed during herpes simplex virus‐1 (HSV‐1) latency, is essential for maintaining viral persistence.^[^ ^23^ ^]^ Moreover, polyadenylated nuclear (*PAN*) RNA, the most abundant lncRNA encoded by Kaposi's sarcoma‐associated herpesvirus (KSHV), which plays a pivotal role in activating viral replication.^[^ ^24^ ^]^ Despite these promising findings of viral lncRNAs, our knowledge of functions of viral expressed lncRNAs in EBV‐infected cells require further investigation. EBV‐associated cancers contain latent forms of the virus, in which the viral DNA is preserved as multicopy episomes.^[^ ^25^, ^26^ ^]^ EBV maintains a stable episome copy number in proliferating cells through the binding of the EBV origin of plasmid replication (*ori*P) with EBNA1. This interaction tethers viral genomes to host chromosomes during mitotic division.^[^ ^27^ ^]^ The process also involves the participation of host factors. Studies have shown that Bromodomain‐containing protein 4 (BRD4, NCBI Gene ID: 23 476), a member of the BET (bromodomain and extra‐terminal domain) family, interacts with the bovine papillomavirus (EPV) E2 protein to link viral DNA to host mitotic chromosomes.^[^ ^28^ ^]^ BRD4 also facilitates the binding of Kaposi's sarcoma‐associated herpesvirus (KSHV) latency‐associated nuclear antigen (LANA) to host chromosomes.^[^ ^29^ ^]^ However, the specific role of BRD4 in the maintenance of the EBV genome remains unclear. Additionally, CCCTC‐binding factor (CTCF, NCBI Gene ID: 10 664), a chromatin‐organizing factor, is involved in loop formation in the EBV *ori*P region.^[^ ^30^, ^31^ ^]^ Whether BRD4 and CTCF function in the same complex to regulate EBV genome maintenance has not yet been investigated.

In this study, we investigate the functions of *lncBART*s in NPC and EBVaGC. Our research confirms that *lncBART*s are highly expressed in EBV‐infected NPC and EVaGC cells, playing a crucial role in maintaining the EBV genome in epithelial cancer cells. We observed that *lncBART*s are localized to nuclear speckles. Our mechanistic investigation demonstrates that *lncBART*s specifically interact with the BRD4/CTCF/EBNA1 complex, acting as an anchor to facilitate the binding of this complex to the *ori*P region. This interaction enhances the association between *ori*P and chromatin, which is essential for maintaining EBV episomes. Notably, the interaction between *lncBART*s and BRD4 complex leads to the upregulation of host proto‐oncogenes, such as MYC (NCBI Gene ID: 4609) and BCL2 (NCBI Gene ID: 596). We propose a mechanism in which EBV utilizes *lncBART*s to modulate host‐virus interactions, altering the cellular environment to support viral latent persistence in infected cells. However, the manipulation of host cell machinery may disrupt normal cellular functions, triggering aberrant signalling pathways and promoting tumorigenesis.

## 2 Results

### 2.1 LncBARTs Mediate the Maintenance of EBV Episomes in EBV‐Infected Epithelial Cells

**Figure 1:**
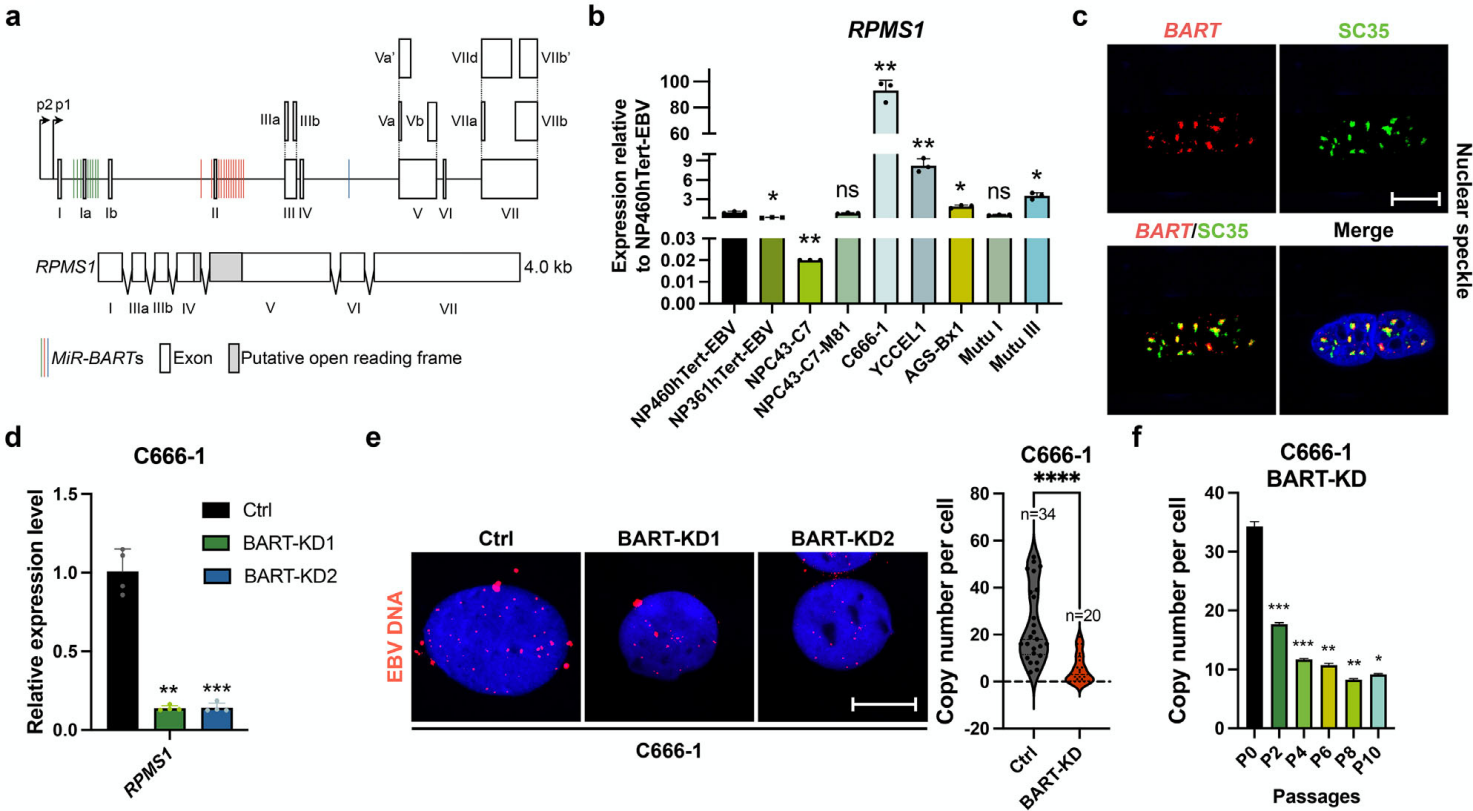
Role of *lncBART*s in maintaining EBV copy number. a) The diagram displays the structure of the major isoform of *lncBART*s, *RPMS1*. Boxes represent exons and the black line shows intronic sequences. Two miRNA clusters are indicated by green (Cluster 1) and red (Cluster 2) bars. b) Expression of *RPMS1* was compared by RT‐qPCR in EBV‐positive cell lines (NP460hTert‐EBV, NP361hTert‐EBV, NPC43‐C7, NPC43‐C7‐M81, C666‐1, YCCEL1, AGS‐Bx1, MutuI, MutuIII). Gene expression was normalized to *GAPDH* and presented as fold‐difference compared to NP460hTert‐EBV (n = 3). c) Representative images of RNA FISH/IF assay for *lncBART*s (red) and nuclear speckles marker SC35 (green) in C666‐1 cells. Scale bar, 10 µm. d) *LncBART*s knockdown cells were generated by transfection with 4 pairs of dox‐inducible shRNAs targeting BART exons. Expression of *lncBART*s knockdown (passage 16) and control C666‐1 cells were analyzed by RT‐qPCR (n = 3). Control represents scramble shRNA with no specific target on the genome. e) Representative images of DNA FISH assay of EBV DNA copies (red) levels in *lncBART*s knockdown (passage 16) and control C666‐1 cells. Scale bar, 10 µm. The accompanying bar charts illustrate the quantification and statistical comparison of EBV copy number between *lncBART*s knockdown (n = 20) and control (n = 34) cells. f) Average EBV copy number per cell in *lncBART*s knockdown and control C666‐1 cells after serial passage (n = 3), determined by qPCR. Gene expression was normalized to that of *GAPDH*. Statistical analysis was performed using unpaired two‐tailed Student's *t*‐test. Data are presented as mean ± SEM. *p < 0.05, **p < 0.01, ***p < 0.001, ns, no significance.

Previous studies have shown that *lncBART*s are expressed in all EBV latency programs and more abundantly in EBV‐associated epithelial tumors than in EBV‐infected B cells.^[^ ^32^, ^33^, ^34^ ^]^ *RPMS1* is the major *lncBART*s transcript, containing most of the known exons (**Figure** **1**a; Figure S1a, Supporting Information).^[^ ^11^, ^15^ ^]^ The expression level of *RPMS1* in the NPC cell line C666‐1 and EBVaGC cell line YCCEL1 was much higher than in NP460hTert‐EBV, NP361hTert‐EBV, NPC43‐EBV, NPC43‐C7‐M81, AGS‐Bx1 (EBVaGC cell line), and Burkitt lymphoma Mutu cell lines with either EBV latency I or latency III (Figure 1b). We confirmed nuclear localization of *lncBART*s in three EBV‐infected epithelial cell lines, C666‐1, NP460Tert‐EBV, and AGS‐Bx1, observing a distinct punctate pattern of *lncBART*s distribution in the nucleus using RNA fluorescence in situ hybridization (FISH) (Figure S1b, Supporting Information). To determine the precise localization of *lncBARTs* within particular nuclear organelles, we performed RNA FISH assays in combination with immunofluorescence (IF) and found that *lncBARTs* appear in the nuclear speckles, but not in nucleolus or paraspeckles (Figure 1c; Figure S1c, Supporting Information). Previously, we used a GapmeR‐mediated RNase H cleavage approach to knock down endogenous *lncBART*s in C666‐1 cells.^[^ ^18^ ^]^ However, this approach generated only a transient knockdown effect, making it impossible to maintain stable cell lines for evaluating the function of *lncBART*s in EBV‐infected NPC cells. In this study, we employed a doxycycline (dox)‐induced system where four pairs of small hairpin RNAs (shRNAs) that target *lncBART*s to generate *lncBART*s knockdown cell lines (BART‐KD) in C666‐1, YCCEL1, and NPC43‐C7‐M81 (Figure 1d; Figure S1d, Supporting Information). These *lncBART*s knockdown cell lines were further passaged and used to investigate the role of *lncBART*s in EBV‐associated cancers. Notably, knockdown of *lncBART*s (after treatment with dox and passage 16 times) resulted in a significant decrease of EBV DNA copy number, as demonstrated through DNA FISH (Figure 1e; Figure S1e, Supporting Information). Quantitative PCR (qPCR) assay was used to assess EBV copy number in *lncBART*s knockdown cell lines every two passages after dox treatment. The results demonstrated a gradual decrease in EBV copy number in these in *lncBART*s knockdown cell lines, with the EBV copy number maintaining a consistently low level after 10 passages (Figure 1f; Figure S1f, Supporting Information).

**Figure 2:**
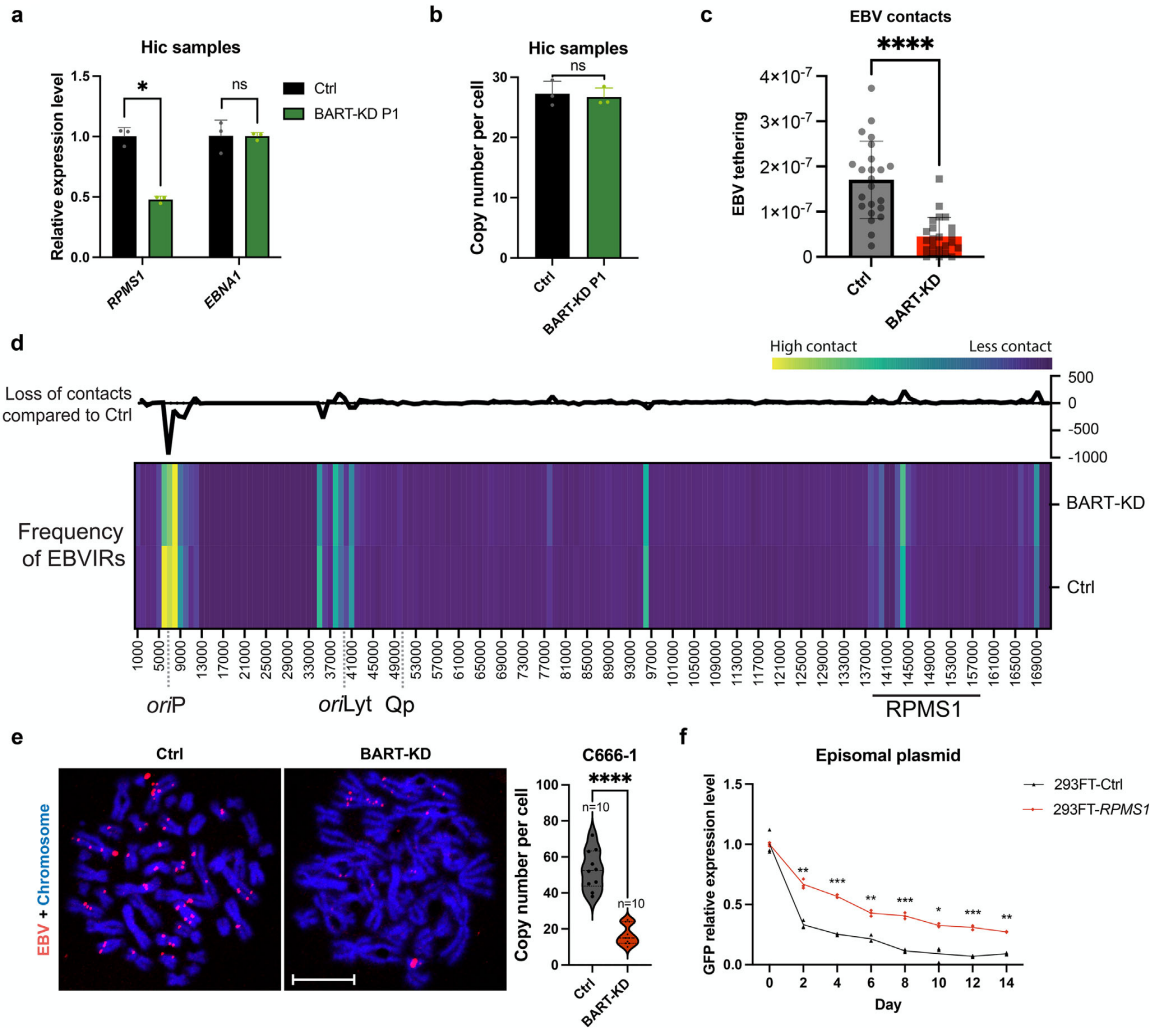
*LncBART*s facilitates EBV‐host chromosome tethering. a) Expression of *RPMS1* and *EBNA1* in *lncBARTs* knockdown (passage 1) and control C666‐1 cells treated with 2 mg mL^−1^ dox for 24 h, assessed using qPCR (n = 3). Gene expression was normalized to that of *GAPDH*. b) The average EBV copy number per cell in *lncBARTs* knockdown (passage 1) and control C666‐1 cells treated with 2 mg mL^−1^ dox for 24 h, assessed using qPCR (n = 3). c) Hi‐C analysis of EBV‐host chromosome tethering in *lncBART*s knockdown and control C666‐1 cells. d) Hi‐C sequencing illustrates the frequency of EBV interacting regions (EBVIRs) on the EBV genome (NC_0 07605.1) in *lncBART*s knockdown and control C666‐1 cells. The line graph across the top highlighted changes at points of interaction between EBV and the host genome in *lncBART*s knockdown cells as compared to control cells. e) Representative images of DNA FISH assay showing the location of EBV DNA copies (red) in mitotic spreads. The *lncBART*s knockdown and control C666‐1 cells were treated with 0.5 mg mL^−1^ of nocodazole (NOC) at 37 °C for 16 h to trigger metaphase arrest. Scale bar, 10 µm. The accompanying bar chart illustrates the quantification and statistical comparison of EBV copy number between *lncBART*s knockdown (n = 10) and control (n = 10) cells. f) The 293FT cells were initially transduced with either *RPMS1* or a control sequence using lentiviral vectors. Episomal vector levels would decrease within cells over time following the withdrawal of puromycin selection. These cells were then transfected with episomal vectors and compare the rates of episomal vector loss with or without *RPMS1* (n=3). The episomal vector levels were monitored every two days using qPCR, targeting the GFP sequence and *GAPDH*. Statistical analysis was performed using unpaired two‐tailed Student's *t*‐test. Data are presented as mean ± SEM. *p < 0.05, **p < 0.01, ***p < 0.001, ****p < 0.0001, ns, no significance.

EBV replicates latent episome replication by anchoring to the host chromatin, ensuring the distribution of the viral genome into daughter cells.^[^ ^27^ ^]^ EBNA1 is recognized as the essential viral protein required for this process.^[^ ^35^ ^]^ To investigate whether *lncBART*s also play a role in EBV latent replication, we examined EBV‐chromatin association using dox‐induced *lncBART*s knockdown C666‐1 cells. After 24 h of dox treatment (without cell passaging, marked with passage 1), *RPMS1* expression was reduced, while *EBNA1* expression and EBV genome copies remained not significant changed (**Figure** **2**a,b). We then performed high‐throughput chromatin conformation capture (Hi‐C) to assess the interactions between the EBV genome and chromosomes in control and passage 1 of *lncBART*s knockdown C666‐1 cells. Our results showed that contact between EBV and host chromosomes was significantly reduced in *lncBART*s knockdown cells (Figure 2c; Figure S1g, Supporting Information). Importantly, a marked reduction in interaction of the EBV *ori*P region with the host chromosome in *lncBART*s knockdown C666‐1 cells was detected (Figure 2d). Notably, we visualized the positioning of EBV DNA and chromosomes under the same treatment conditions as the Hi‐C samples, followed by induction of metaphase arrest with the microtubule‐depolymerizing agent nocodazole (NOC). DNA FISH results demonstrated that EBV DNA colocalized with mitotic chromosomes in control cells, whereas in *lncBART*s knockdown cells, there was a reduction in EBV DNA colocalization to chromosomes (Figure 2e; Figure S1h, Supporting Information). To further verify the role of *lncBART*s in maintaining EBV latent genome, we utilized an episomal vector containing EBV‐derived *ori*P/EBNA1 components which are known essential components for ensuring plasmid duplication during cell division^[^ ^36^ ^]^ (Figure S1i, Supporting Information). Upon withdrawal of puromycin selection, the episomal vector within the cells decreased over time. Interestingly, we found that the decrease of episome was more significant in the control 293TF cells compared to those overexpressing *RPMS1* (Figure 2f). These results suggest that *lncBART*s are involved in the EBV *ori*P/EBNA1 latent replication process to maintain EBV genome in infected cells.

### 2.2 LncBARTs Engagement with Host Factors at the *ori*P Region of EBV

**Figure 3:**
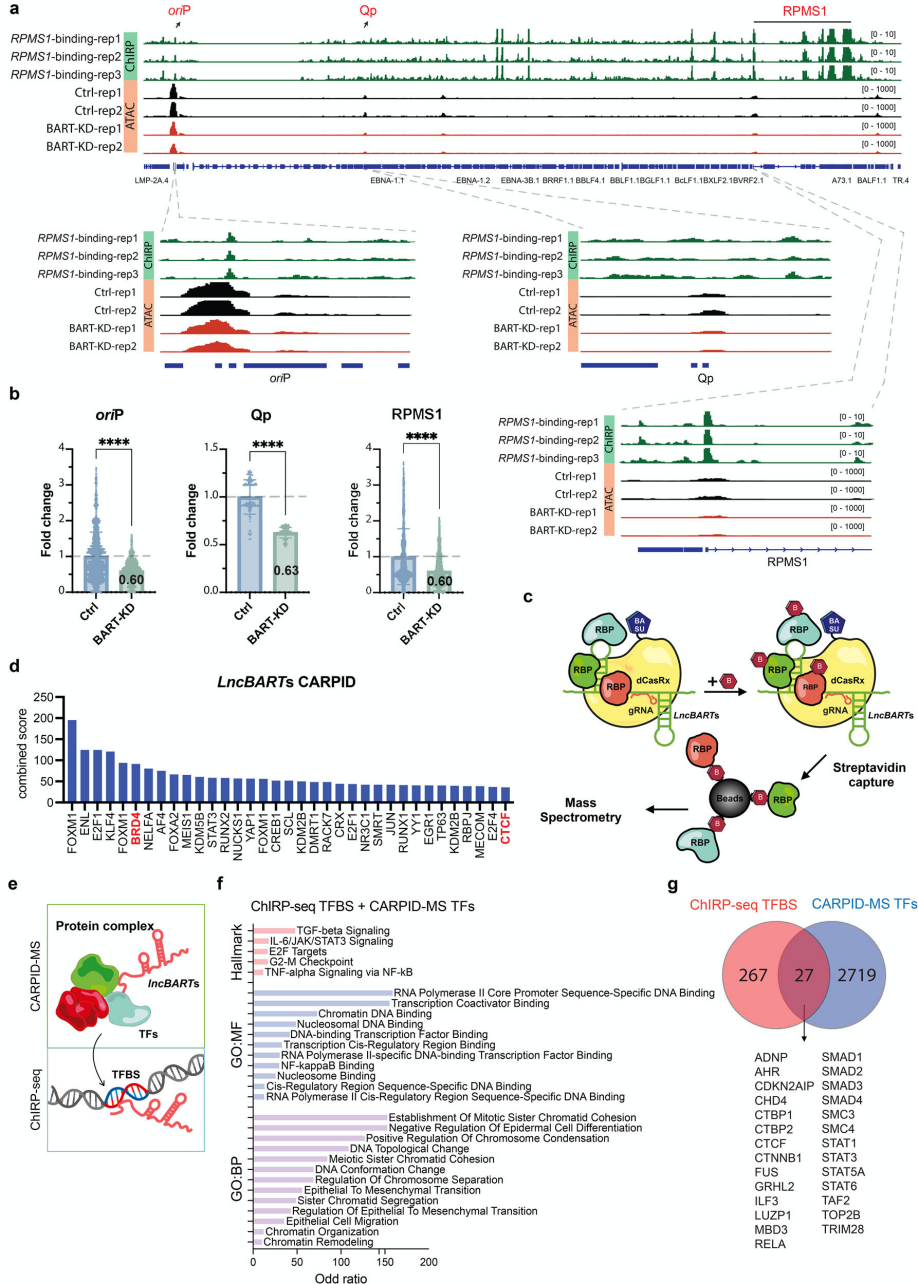
*LncBART*s interact with host factors at the *ori*P region. a) ChIRP‐seq for *lncBART*s performed in triplicate and ATAC‐seq replicates indicate distribution of DNA that *lncBART*s bind to and the open accessibility sites, respectively, across the EBV genome (NC_0 07605.1) for *lncBART*s knockdown and control C666‐1 cells. The control samples ("Ctrl‐rep1" and "Ctrl‐rep2") of ATAC‐seq were cells infected with a non‐targeting shRNA lentivirus and treated with 2 mg mL^−1^ dox for the same duration as the dox‐induced *lncBART*s knockdown cells. The results were visualized by IGV. The DNA regions occupied by *lncBART*s and open accessibility sites determined through ChIRP‐seq and ATAC‐seq adjacent to *ori*P, Qp and *RPMS1* are highlighted and illustrated in detail in the lower panel. b) Bar graph showing quantitative ATAC‐seq signals at the *ori*P region in *lncBART*s knockdown and control C666‐1 cells. Fold change between control and *lncBART*s knockdown is indicated on each column. c) Schematic illustration of the CARPID assay workflow. CARPID combines dCasRx with the engineered biotin ligase BASU to track the expression of proteins interacting with target RNA in live cells. Biotin is represented by “B”. d) Host proteins enriched by *lncBART*s in CARPID were ranked by a combined score (odds ratio * Z‐score). A higher combined score indicates greater significance. e) Overlap of transcription factor (TF) identified via CARPID mass spectrometry and transcription factor binding sites (TFBS) interacting with *lncBART*s, along with a list of the 27 intersecting genes. f) Gene Ontology (GO), which examines molecular functions (MF) and biological processes (BP), and Hallmark analysis of the 27 intersecting genes. The odds ratio represents the strength of enrichment. g) Overlap of transcription factors (TFs) identified by CARPID mass spectrometry with transcription factor binding sites (TFBS) associated with *lncBART*s detected by ChIRP. Statistical analysis was performed using unpaired two‐tailed Student's t‐test. Data are presented as mean ± SEM. ****p < 0.0001.

To further investigate the regulatory role of *lncBART*s at the *ori*P region, we performed chromatin isolation by RNA purification followed by deep sequencing (ChIRP‐seq). ChIRP‐seq analysis of *lncBART*s showed widespread distribution of *lncBART*s across the EBV genome, with a concentration in *ori*P and *RPMS1* regions, confirming the genomic occupancy of *lncBART*s on EBV genome (**Figure** **3**a‐ChIRP). Furthermore, we found that knockdown of *lncBART*s led to decreased accessibility in these regions, as determined by assay of transposase‐accessible chromatin sequencing (ATAC‐seq) in C666‐1 cells (Figure 3a‐ATAC and Figure 3b). Since the EBV episome copy number decreases following *lncBART*s knockdown, the quantified ATAC‐seq read counts at *ori*P, Qp and RPMS1 regions were normalized to the total number of EBV reads in each sample (Figure S2a, Supporting Information). This analysis demonstrated that the observed changes in chromatin accessibility are not solely due to episome loss. EBV *ori*P is critical for EBV latent replication and stable maintenance in infected cells while Qp is the promoter that drives EBNA1 expression in EBV‐infected NPC and EBVaGC cells. Binding to the *RPMS1* region could potentially be a result of the antisense probes of *lncBART*s binding to this region of EBV genome. Given that apoptosis can significantly impact host chromatin, it is important to determine whether the observed chromatin inaccessibility caused by knockdown of *lncBART*s is associated with apoptosis. To test this possibility, we conducted a series of experiments using H_2_O_2_ to induce apoptosis and the pan‐caspase inhibitor z‐VAD‐fmk to inhibit apoptosis in C666‐1 cells. First, we established the optimal concentration of H_2_O_2_ for inducing apoptosis in C666‐1 cells (Figure S2b, Supporting Information). To investigate whether apoptosis influences chromatin accessibility, cells were pre‐treated with z‐VAD‐fmk prior to H_2_O_2_ exposure. Pre‐treatment with z‐VAD‐fmk significantly reduced late apoptosis levels in C666‐1 cells (Figure S2c, Supporting Information). Subsequently, ATAC‐seq was performed on cells pre‐treated with z‐VAD‐fmk, followed by H_2_O_2_ exposure. Our analysis revealed that approximately half of the genes downregulated in H_2_O_2_‐treated cells were not overlapping with those downregulated following *lncBART*s knockdown, indicating that the chromatin accessibility changes induced by *lncBART*s knockdown are not identical to those associated with apoptosis process (Figure S2d, Supporting Information). Importantly, analysis of the chromatin accessibility at the EBV *ori*P region showed that neither H_2_O_2_‐induced apoptosis nor apoptosis inhibition with z‐VAD‐fmk significantly affected accessibility at *ori*P (Figure S2e, Supporting Information). In contrast, decreased accessibility at *ori*P was observed in *lncBART*s knockdown samples (Figure 3b). These findings suggest that chromatin accessibility at EBV *ori*P, influenced by *lncBART*s knockdown, is independently of apoptosis in EBV infected cells. Overall, these results validate that *lncBART*s bind to the EBV *ori*P regions and modulate chromatin accessibility, thereby affecting the interaction between *ori*P and the host chromosome to regulate EBV episome maintenance in infected cells.

To identify cellular proteins that interact with *lncBART*s in EBV‐infected cells, we employed the CRISPR‐assisted RNA‐protein interaction detection (CARPID) assay, which features augmented targeting specificity and reduced background noise (Figure 3c).^[^ ^37^ ^]^ This method integrates CRISPR‐CasRx‐mediated RNA targeting and proximity labelling to isolate host proteins that interact with *lncBART*s (detailed in Materials and Methods section). We quantified 2500 host proteins as potential components of the *lncBART*s interactome. Using a combined score (odds ratio * Z‐score), we ranked those related to mitosis and selected BRD4 and CTCF, both reported to play key roles in viral replication, as the candidate to interact with *lncBART*s (Figure 3d). We overlapped transcription factors (TFs) identified via CARPID mass spectrometry with transcription factor binding sites (TFBS) interacting with *lncBART*s detected by ChIRP (Figure 3e). This revealed 27 shared genes, including key factors like SMAD, STAT, CTBP, SMC complexes, and TOP2B. Gene Ontology (GO) analysis highlighted RNA polymerase II core promoter sequence‐specific DNA binding, and the establishment of mitotic sister chromatid cohesion, with the highest odds ratios (Figure 3f). These results indicate that these proteins may associate with *lncBART*s as part of a regulatory complex which is associated with gene expression and chromatin reprogram in EBV‐infected cancer cells.

### 2.3 LncBARTs Interact with BRD4

**Figure 4:**
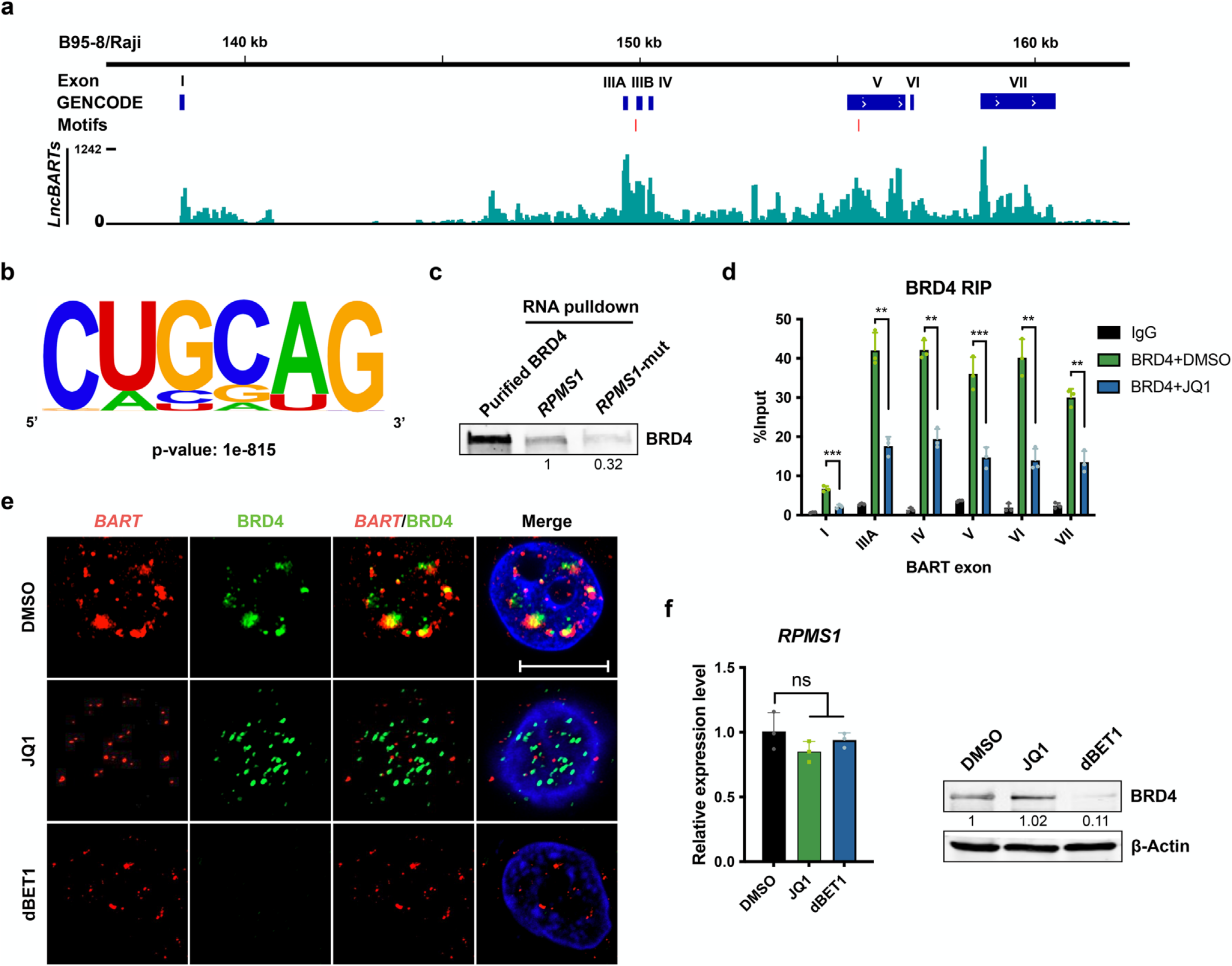
Interaction of *lncBART*s with the N‐terminal of BRD4. a) Genome browser display of BRD4 binding to BART exons (NC_0 07605.1) based on CLIP‐seq data in C666‐1 cells. The red lines highlight the location of BRD4 binding motifs on BART exons. b) Sequence logos corresponding to enriched sequence elements identified by motif analysis of BRD4 SpyCLIP clusters. c) RNA pulldown assay using 5 µg of biotin‐labeled wild‐type *RPMS1* or 5 µg of a mutant *RPMS1* (*RPMS1*‐mut) with a deletion in the BRD4 binding motif (generated by IVF) to capture 5 µg of purified BRD4 protein. In the western blot analysis, 5 µg of purified BRD4 protein was used as the input control. d) RIP analysis using BRD4‐specific or IgG control antibody was performed on C666‐1 cell extracts (n = 3), and *lncBART*s exons detected via RT‐qPCR. Comparisons were made between the 48 h, 500 µM JQ1‐treated group and the untreated control group. e) Representative images of RNA FISH/IF assay for *lncBART*s (red) and BRD4 (green) in C666‐1 cells treated with DMSO for 48h, 500 nM JQ1 for 48 h or 250 nM dBET1 for 24 h. Scale bar, 10 µm. f) Analysis of the levels of *RPMS1* RNA and BRD4 protein after treatment as in d) (n = 3), measured by RT‐qPCR and western blot, respectively. Statistical analysis was performed using unpaired two‐tailed Student's *t*‐test. Data are presented as mean ± SEM. **p < 0.01, ***p < 0.001, ns, no significance.

One of the major *lncBART*s‐interacting factors identified in the CARPID experiment is BRD4 (Figure 3d), a member of the BET family.^[^ ^38^ ^]^ BRD4 has been reported to assist in the replication of viruses.^[^ ^28^, ^29^ ^]^ Furthermore, BRD4 plays a crucial role in regulating gene expression and is recognized as an essential factor for expression from *MYC* and other tumor driver genes.^[^ ^39^ ^]^ Therefore, BRD4 was selected to investigate its role in facilitating EBV genome replication through interaction with *lncBART*s. To further validate a direct interaction between *lncBART*s and BRD4, we used the SpyTag‐based CLIP (SpyCLIP) method to analyze BRD4‐*lncBART*s binding in C666‐1 cells. SpyCLIP is a covalent link‐based CLIP method noted for its high efficiency and accuracy, which can resist harsh washing conditions, allowing effective removal of non‐specific interactions (Figure S3a–c, Supporting Information). The analysis revealed that most of the RNA that binds with BRD4 belongs to categories such as protein coding, snoRNA, and lncRNA (Figure S2d, Supporting Information). The reads from SpyCLIP were then mapped to the annotated EBV genome (GenBank accession number NC_0 07605.1) indicating binding site locations. In the Integrative Genome visualization (IGV), it was observed that a substantial portion of BRD4 signal identified by SpyCLIP was specifically enriched across the seven exons of *lncBART*s (**Figure** **4**a). Moreover, using HOMER and STREME, we identified the RNA motif CUGCAG as the highest‐scoring BRD4 recognition sequence, located in *lncBART*s exons III and V (Figure 4b). We verified the BRD4 binding site by constructed a mutant version of *RPMS1* (*RPMS1*‐mut) in which the BRD4 binding motif CUGCAG, identified by BRD4‐CLIP, was replaced with AAAAAA. RNA pulldown assays using this mutant *RPMS1*‐mut and the wild‐type *RPMS1* generated via in‐vitro transcription to pulldown purified BRD4 protein. The western blot analysis demonstrated a reduced interaction between BRD4 and *RPMS1*‐mut compared to the wild‐type *RPMS1* (Figure 4c).

To determine which domain in BRD4 mediates association with *lncBART*s, we performed RNA pulldown experiments with biotinylated full‐length *lncBART*s and purified recombinant truncated BRD4 protein expressed in E. coli. Strong signals were detected for the truncated BRD4 with the BD domain (1‐444), but not with truncations containing both the BD and ET domains (1‐722) or the PID domain (1047‐1362 and 1224‐1362), indicating that *lncBART*s specifically interact with the N‐terminal of BRD4 (Figure S2e, Supporting Information). BD domains exhibit binding affinity to acetylated lysines on target proteins.^[^ ^38^ ^]^ JQ1, a BRD4 inhibitor, specifically targets the BD domains of BRD4, competitively disrupting the recognition of acetyl‐lysine on chromatin. This interaction of *lncBART*s and BD domains was further validated through RNA immunoprecipitation (RIP) qPCR assays and treatment of JQ1 can disrupt this association (Figure 4d). RNA FISH/IFA assay with BRD4 inhibitor JQ1 and dBET1 (a BRD4 protein degrader) treatment in C666‐1 cells showed the same effect, in which the clustered *lncBART*s were dispersed (Figure 4e). Neither JQ1 nor dBET1 treatment altered the expression of *lncBART*s, and JQ1 treatment did not significantly affect BRD4 protein levels (Figure 4f). These results clearly confirm an interaction between *lncBART*s and the BD domains of BRD4 in EBV‐infected cells.

### 2.4 LncBARTs Collaborate with the BRD4/CTCF/EBNA1 Complex Modulate EBV Replication at the *ori*P Region

**Figure 5:**
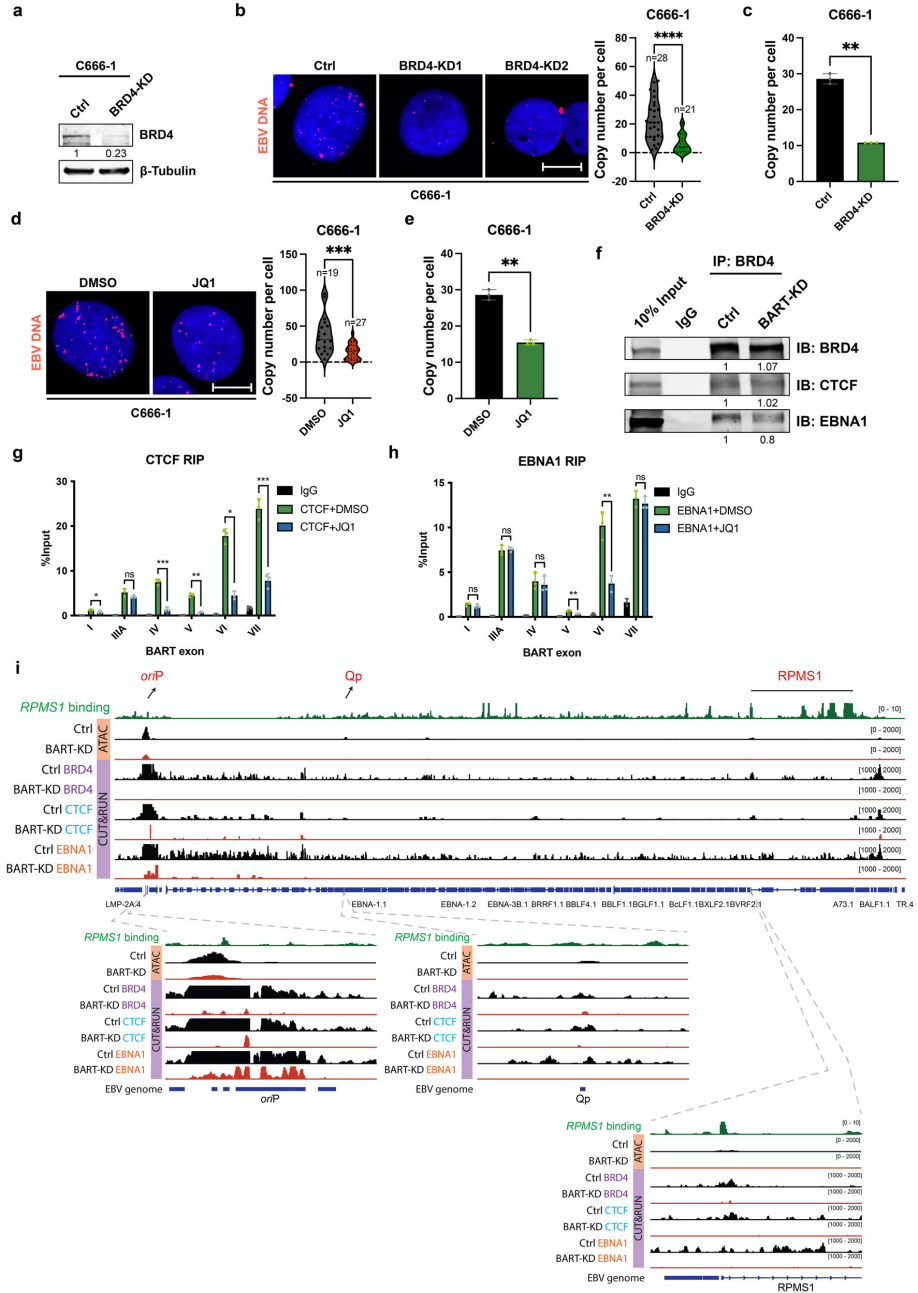
The BRD4/CTCF/EBNA1 complex support *lncBART*s’ regulatory on *ori*P. a) Expression levels of BRD4 were evaluated by western blot in BRD4 knockdown and control C666‐1 cells. b) Representative images of DNA FISH assay of EBV DNA copies (red) in BRD4 knockdown and control C666‐1 cells. Scale bar, 10 µm. The accompanying bar charts illustrate the quantification and statistical comparison of EBV copy number between BRD4 knockdown (n = 21) and control (n = 28) cells. c) EBV copy number as determined by qPCR in BRD4 knockdown and control C666‐1 cells (n = 3). d) Representative images of DNA FISH assay of EBV DNA copies (red) in C666‐1 cells treated with DMSO or 500 nM JQ1 for 7 days, during which the cells were passaged once. Scale bar, 10 µm. The accompanying bar charts illustrate the quantification and statistical comparison of EBV copy number between JQ1 (n = 27) and DMSO (n = 19) treated C666‐1 cells. e) EBV copy number determined by qPCR in C666‐1 cells treated with DMSO or 500 nM JQ1 for 7 days (n = 3). f) Co‐IP assays showing BRD4 interaction with CTCF and EBNA1 in *lncBART*s knockdown and control C666‐1 cells. Cell extracts were immunoprecipitated using a BRD4 antibody and then western blot analysis performed with antibodies against CTCF and EBNA1. RIP analysis was performed on C666‐1 cell extracts using either g) CTCF‐specific, h) EBNA1‐specific, or IgG control antibody, followed by RT‐qPCR detection of *lncBART*s exons. Comparisons were made between cells treated with 500 µM JQ1 for 48 h and untreated control group (n = 3). i) The CUT&RUN‐seq track images display the binding profiles of BRD4 and CTCF on the EBV genome (NC_0 07605.1) in *lncBART*s knockdown and control C666‐1 cells. This is visualized alongside *lncBART*s ChIRP (top track: RPMS1 binding) and ATAC‐seq analysis using IGV. Areas of co‐occupancy adjacent to *ori*P, Qp and *RPMS1* revealed in ChIRP‐seq, ATAC‐seq, BRD4 and CTCF CUT&RUN‐seq are highlighted and illustrated in detail in the lower panel. Statistical analysis was performed using unpaired two‐tailed Student's *t*‐test. Data are presented as mean ± SEM. **p < 0.01, ***p < 0.001, ****p < 0.0001.

To explore the potential role of *lncBART*s‐BRD4 interaction in regulating EBV latency, we generated BRD4 knockdown C666‐1 and YCCEL1 cell lines using dox‐induced, stably expressed shRNAs targeting BRD4 (**Figure** **5**a; Figure S3a, Supporting Information). We reasoned knockdown of BRD4 may cause a similar effect to the knockdown of *lncBART*s on EBV genome copy numbers, as shown above (Figure 1d–f). To examine this possibility, we performed DNA FISH and quantitative qPCR analyses to examine EBV copy numbers in both C666‐1 and YCCEL1 cells with BRD4 knockdown and compared with control cells. Our results demonstrated a significant decrease in EBV copy number in both BRD4 knockdown cell lines (Figure 5b,c; Figure S3b,c, Supporting Information). To further validate the role of *lncBART*s‐BRD4 in regulating EBV genome copy numbers, we demonstrated that treatment of C666‐1 cells with the BRD4 inhibitor JQ1 also led to a decrease in EBV viral copies (Figure 5d,e). Considering that C666‐1 cells have an approximate doubling time of 3 days, viral copy number was assessed by EBV DNA FISH and qPCR after 7 days of treatment with JQ1. These findings indicate that BRD4 plays a critical role in the maintenance of EBV genome in EBV‐associated NPC and EBVaGC cells.

In addition to BRD4, another cellular factor identified to interact with *lncBART*s is CTCF. A previous study indicated that CTCF is involved in loop formation in the EBV *ori*P region.^[^ ^40^ ^]^ Since EBNA1 is essential for maintaining the EBV latent episome via *ori*P binding, there is significant interest in determining how the BRD4/CTCF/EBNA1 complex is associated with *lncBART*s. IF assay showed the co‐localization of BRD4 with both CTCF and EBNA1 (Figure S3d, Supporting Information). Additionally, the association of *lncBART*s with CTCF and EBNA1 was validated through RIP‐qPCR and RNA FISH/IF assays (Figure 5g,h; Figure S3e, Supporting Information). RIP‐qPCR results showed that treatment with JQ1 decreased the association of exons I, IV, V, VI, and VII of *lncBART*s with CTCF, as well as the binding of exons V and VI of *lncBART*s to EBNA1. These results suggest that *lncBART*s interact with CTCF and EBNA1 via the BD domain of BRD4. Furthermore, co‐immunoprecipitation (co‐IP) result indicated that CTCF and EBNA1 were interacted with BRD4 (Figure 5f). Knockdown of *lncBART*s did not affect the BRD4‐CTCF interaction but reduced the association between BRD4 and EBNA1, suggesting that *lncBART*s specifically mediate the interaction between BRD4 and EBNA1.

Cleavage under targets and release using nuclease sequencing (CUT & RUN‐seq) was conducted in *lncBART*s knockdown and control C666‐1 cells. We found enrichment of BRD4, CTCF and EBNA1 occupation on *ori*P, which overlapped with the region bound by *lncBART*s, as well as with differentially accessible sites, as determined by ATAC‐seq. Knockdown of *lncBART*s led to significant decreases in the presence of BRD4, CTCF and EBNA1 on the *ori*P region (Figure 5i). A similar reduction was observed at the Qp promoter and the RPMS1 locus, with lower intensity compared to *ori*P. The result suggested that *lncBART*s might anchor the binding of BRD4/CTCF/EBNA1 complexes to the *ori*P region, thereby regulating the maintenance of EBV latent episome replication. The knockdown of *lncBART*s resulted in decreased binding of H3K4me3 and H3K27ac histone modifications at the *ori*P and Qp regions, which may suggest a potential regulatory role of *lncBART*s on enhancer activity (Figure S3f, Supporting Information). All sequencing data in this study were analyzed using the wild‐type EBV genome assembly based on B95/Raji (GenBank accession number NC_0 07605.1). The cell model used is C666‐1. To account for potential variations between the two sequences, we performed BLAST analysis of the viral sequences from both the wild‐type EBV genome assembly and the C666‐1 genome (GenBank accession number KC617875.1), revealing a 99.02% similarity (Table S2, Supporting Information). Additionally, we reanalysed the ChIRP and CUT&RUN‐seq data using the C666‐1 reference genome, obtaining results consistent with those derived from the wild‐type EBV genome assembly (Figure S3g–i, Supporting Information). These findings suggest a functional association between *lncBART*s and BRD4/CTCF/EBNA1 complexes in EBV latent episome replication, as well as the potential regulation of Qp for EBNA1 expression by *lncBART*s.

### 2.5 LncBARTs‐BRD4 Interaction Regulates MYC Expression

**Figure 6:**
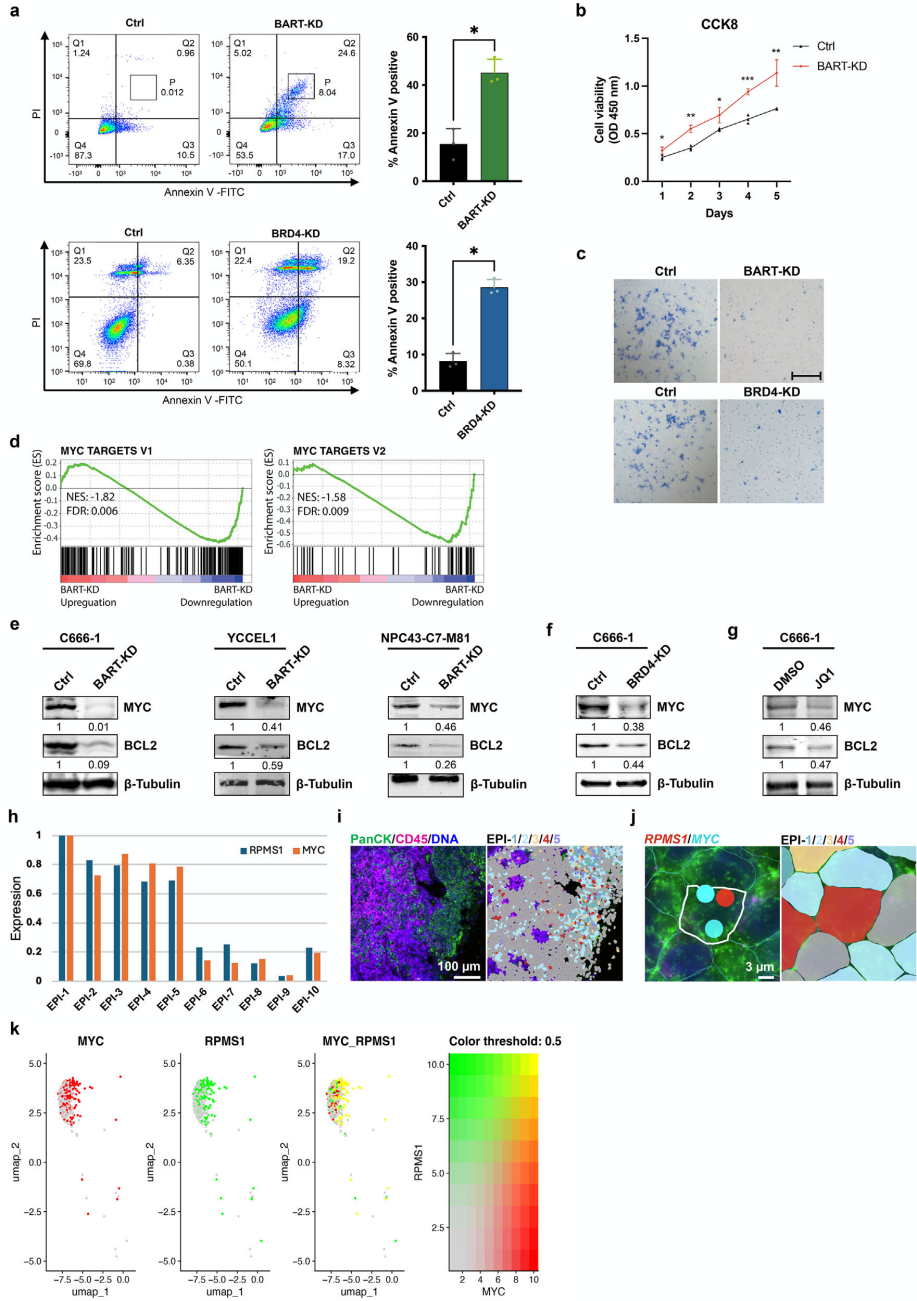
The role of *lncBART*s in driving tumorigenesis by regulating *MYC* expression. a) Annexin V and PI staining of *lncBART*s and BRD4 knockdown (passage 19) and control C666‐1 cells were analyzed by flow cytometry. Experiments were repeated three times and representative images are shown. The adjacent bar chart quantifies the percentage of Annexin V positive cells. b) CCK8 assays in *lncBART*s knockdown (passage 18) and control C666‐1 cells for 5 consecutive days following the treatment of 2 mg/ml dox to determine cell viability (n = 3). c) Transwell invasion assay indicates the invasion abilities in *lncBART*s and BRD4 knockdown (passage 20) and control C666‐1 cells. Scale bar, 100 µm. d) GSEA enrichment plots, using the Hallmark gene set, demonstrate that Hallmark MYC targets v1 and MYC targets v2 are enriched in *lncBART*s knockdown C666‐1 compared to control cells in RNA‐seq. e) The expression levels of MYC and BCL2 in *lncBART*s knockdown C666‐1, YCCEL1, and NPC43‐C7‐M81 cells, along with their controls, were assessed using western blot. f) The expression levels of MYC and BCL2 in BRD4 knockdown and control C666‐1 cells were assessed using western blot. g) The expression levels of MYC and BCL2 in C666‐1 cells treated with 500 nM JQ1 or DMSO for 48 h were assessed using western blot. h) The bar graph illustrates the expression levels of *RPMS1* and *MYC* in clusters EPI‐1 to EPI‐10. Clusters EPI‐1 to EPI‐5 are demonstrate enhancer activation, as determined through single‐cell RNA‐seq analysis. i) The Single‐cell spatial analysis with the CosMx Spatial Molecular Imager (SMI) analysis image demonstrates the expression of the *RPMS1* and *MYC* genes within an NPC biopsy. Cell types within the tissue are identified using PanCK (epithelial cancer cells, green), CD45 (lymphocyte cells, purple) and DNA (blue) staining, as shown on the left. Clusters EPI‐1 (blue), ‐2 (light blue), ‐3 (yellow), ‐4 (brown), and ‐5 (purple) are displayed on the right. Scale bar, 100 µm. j) An enlarged view of the boxed region in H displays a single cell expressing *RPMS1* (red) and *MYC* (blue) genes on the left, while the different cell types are shown on the right. Scale bar, 3 µm. k) The UMAP dimension reduction displays cellular maps at a single‐cell resolution, demonstrating the expression of *MYC* and *RPMS1*. The red color represents exclusive *MYC* expression, the green color signifies exclusive *RPMS1* expression, and the yellow color indicates co‐expression of both. Statistical analysis was performed using unpaired two‐tailed Student's *t*‐test. Data are presented as mean ± SEM. *p < 0.05, **p < 0.01, ***p < 0.001.

We demonstrated that *lncBART*s interact with BRD4 and regulate EBV latent replication in NPC and EBVaGC cells. BRD4, a known transcriptional and epigenetic regulator implicated in cancer development, was further analyzed in NPC tissues using immunohistochemistry (IHC) (Figure S4a, Supporting Information). Immunohistochemical analysis revealed that BRD4 expression was predominantly localized to cancer cells, with minimal staining observed in infiltrating lymphocytes. Notably, no BRD4 expression was detected in healthy control tissues, suggesting its potential role in NPC progression. We hypothesize that EBV‐expressed *lncBART*s hijack host BRD4 machinery to facilitate viral replication, thereby disrupting normal cellular processes, altering signaling pathways, and driving oncogenic cell fate. Supporting this, Annexin V/PI staining showed that knockdown of *lncBART*s or BRD4 in C666‐1 cells (passage 19) significantly increased apoptosis (**Figure** **6**a). Similarly, *lncBART*s knockdown reduced C666‐1 cell viability, as evidenced by CCK‐8 assays (Figure 6b). Furthermore, transwell invasion assays demonstrated diminished invasive capabilities in both C666‐1 and YCCEL1 cells (passage 20) following *lncBART*s or BRD4 depletion (Figure 6c; Figure S4b, Supporting Information). Collectively, these findings highlight the critical role of *lncBART*s and BRD4 in modulating apoptosis, proliferation, and invasion in EBV‐associated cancer cells.

ATAC‐seq analysis identified a total of 7664 differentially accessible chromatin peaks were identified. Heatmap visualization revealed a marked decrease in global chromatin accessibility signals in *lncBART*s knockdown C666‐1 cells compared to controls (Figure S4c, Supporting Information). The MA plot generated by DiffBind further demonstrated that the majority of differential accessibility manifested as a loss of open chromatin regions following *lncBART*s knockdown (Figure S4d, Supporting Information). Notably, these differentially accessible regions were predominantly localized to promoter regions (Figure S4e, Supporting Information). These findings suggest that *lncBART*s may epigenetically regulate host gene expression linked to cell growth and carcinogenesis.

To further investigate how *lncBART*s regulate host genes, we performed RNA‐seq on C666‐1 cells following *lncBART*s or BRD4 knockdown. This analysis identified 774 downregulated and 448 upregulated genes common to both knockdown conditions (Figure S4f, Supporting Information). Functional annotation revealed that downregulated genes were enriched in processes such as stress‐responsive RNA polymerase II‐mediated transcription initiation, mitotic spindle checkpoint regulation, endocytic recycling, intrinsic apoptotic pathway modulation, and suppression of cellular processes. Conversely, upregulated genes were associated with DNA replication‐dependent chromatin assembly, centromeric chromatin remodelling, mitotic DNA replication/integrity checkpoint signaling, and DNA unwinding during replication (Figure S4g, Supporting Information). Gene Set Enrichment Analysis (GSEA) on Hallmark pathways was used to identify significant differentially expressed gene sets in RNA‐seq under different conditions. GSEA enrichment plots revealed that several Hallmark categories including MYC targets v1, MYC targets v2 (Figure 6d), DNA repair, TNFA signalling via NFKB, p53 pathway, G2M checkpoint and mitosis spindle are significantly enriched in *lncBART*s knockdown cells compared to control cells in RNA‐seq (Figure S4h, Supporting Information).

Notably, RNA‐seq highlighted reduced expression of MYC and BCL2, key oncogenic regulators, in *lncBART*s‐knockdown C666‐1 cells. These findings were validated by qPCR and western blot across C666‐1, YCCEL1, and NPC43‐C7‐M81 cell lines (Figure 6e; Figure S4i, Supporting Information). Similarly, both BRD4 knockdown and treatment with JQ1 also downregulated *MYC* and *BCL2* expression in C666‐1 cells (Figure 5f‐g and Figure S4j, Supporting Information). Together, these results suggest that EBV‐encoded *lncBART*s cooperate with BRD4 to dysregulate host gene expression, promoting aberrant activation of oncogenic drivers like MYC and BCL2 in EBV‐associated epithelial cancers.

To investigate potential cooperative regulation between *lncBART*s and MYC in EBV‐infected NPC cells, we analyzed NPC tissues using single‐cell RNA‐seq, focusing on *RPMS1* and *MYC* expression in PanCK+ epithelial clusters (EPI‐1 to EPI‐10), which were defined by distinct transcriptional profiles. Enhancer activation was prominent in clusters EPI‐1 to EPI‐5, where both *RPMS1* and *MYC* showed concurrent upregulation (Figure 6h). Spatial single‐cell analysis of an NPC biopsy using the CosMx SMI further demonstrated that EPI‐1 to EPI‐5 clusters predominantly comprised cancer cells (Figure 6i). A representative cell within this population exhibited robust co‐expression of *RPMS1* and *MYC* (Figure 6j). Consistent co‐expression patterns of *RPMS1* and *MYC* in cancer cells were confirmed by UMAP visualization at single‐cell resolution (Figure 6k). These results suggest a significant correlation between *RPMS1* and *MYC* expression in NPC tumor cells.

Taken together, these results strongly suggest a mechanism by which EBV‐expressed *lncBART*s integrate into BRD4 complexes to regulate host gene expression, leading to aberrant expression of tumor‐driving genes, such as *MYC* and *BCL2*, in EBV‐associated epithelial cancers.

## 3 Discussion

EBV establishes different latency programs in vivo with varying outcomes. Although the majority of the population are asymptomatic carriers for their entire life, EBV is known to be associated with several human cancers.^[^ ^4^ ^]^ EBV maintains multicopy episomes in tumor cells, and elucidating the mechanisms governing viral‐episome maintenance is critical for understanding the molecular basis of EBV‐driven tumorigenesis. EBV‐immortalized lymphoblastic cell lines (LCL) provide an important in vitro model for studying EBV latent replication in human lymphoblastoid cells and associated malignancies.^[^ ^41^ ^]^ Recent studies showed that the 3D structure of the EBV genome differs in type I, II and III latencies of EBV infection.^[^ ^42^ ^]^ It is known that EBV‐expressed EBNA2 plays an essential role in B cell immortalization, in conjunction with actions of other EBNAs and LMP1, and has key functions in controlling the EBV regulome in lymphoblastoid cells.^[^ ^43^, ^44^ ^]^ The study of EBV association in epithelial cancers, namely NPC and EBVaGC which demonstrate latency II EBV infection, has been hampered by a lack of infection models. EBV gene expression is tightly regulated in NPC and EBVaGC, with only genes essential for latency being expressed. There is strong interest in understanding how EBV maintains the latency program in these cells in the absence of EBNA2 expression, as the latency program (II) of EBV in NPC and EBVaGC cells includes only expression of EBNA1, LMP1/2 and non‐coding RNAs which include EBERs and BARTs (*miR‐BART*s and *lncBART*s). EBNA1 is essential for EBV latent episome replication and LMP1 is a viral oncoprotein. Numerous functions of *miR‐BART*s have been reported, and EBER2 was found to interact with PAX5 to regulate EBV lytic replication.^[^ ^45^, ^46^ ^]^ While *lncBART*s are abundantly expressed in NPC cells,^[^ ^32^ ^]^ the biological significance of EBV *lncBART*s remains largely uncharacterized. The mechanism by which EBV maintains its episome in epithelial cells may provide insight into EBV oncogenesis in NPC and EBVaGC. The results of this study indicate that *lncBART*s play a crucial role in maintaining EBV episome persistence in NPC and EBVaGC cells. It is tempting to consider that *lncBART*s could function as a regulator, similar to EBNA2 in LCLs, to regulate EBV latency in NPC and EBVaGC cells.

Previous studies have shown that expression of *lncBART*s is elevated in NPC cells compared to EBV‐infected B cells, suggesting an important role for EBV latency in epithelial cancers.^[^ ^32^, ^34^ ^]^ In this study, we attempted to modulate the levels of *lncBART*s in EBV‐harboring NPC and gastric carcinoma cell lines and combined this with functional genomic analysis to understand the impact of *lncBART*s on EBV latent replication. Our results showed that *lncBART*s are critical for maintaining EBV copy numbers in both NPC and EBVaGC cell lines. We further demonstrated that *lncBART*s regulate EBV latency through interaction with BRD4 and CTCF regulatory machineries. BRD4 functions as an epigenetic reader that recognizes and binds to acetylated histones, exhibits preferential bind to di‐ and tetra‐acetylated histone H4 and diacetylated H3,^[^ ^47^ ^]^ and associates with mitotic chromosomes, a property that may facilitate its role in maintaining viral episomes during cell division.^[^ ^48^ ^]^ This aligns with our finding that BRD4 is required for maintaining EBV episomal copy numbers. CTCF has been well characterized for its role in regulating EBV type I and III latencies.^[^ ^49^ ^]^ It is unsurprising that CTCF is also involved in the EBV latency program in epithelial cells. Our data suggests that EBV may adopt a different mechanism than that found in EBV‐immortalized LCLs, relying on *lncBART*‐BRD4 interaction for replication in latently infected epithelial cells. Our findings strongly support the idea that *lncBART*s involvement in EBV latent replication is through their interaction with the BRD4‐CTCF complex. Interestingly, BRD4 also plays a key role in the replication of HPV and MCV.^[^ ^50^, ^51^ ^]^ *Ori*P is the origin of EBV genome replication and also functions as a transcription enhancer for Cp in the latency III program. Since EBV in both NPC and EBVaGC cells utilizes the latency II program and does not express EBNAs from Cp, we suggest that enrichment of *lncBART*s‐BRD4/CTCF near *ori*P may be associated with replication of the EBV genome. Indeed, we showed through Hi‐C analysis that contact between EBV, and host chromosomes is significantly reduced in *lncBART*s knockdown cells (Figure 2a). It is well characterized that EBNA1 binds to *ori*P and is essential for *ori*P replication.^[^ ^52^ ^]^ We identified *lncBART*s as another viral product, likely acting in conjunction with EBNA1, associated with the *ori*P region. Replication from EBV *ori*P also requires host initiation factors.^[^ ^53^, ^54^ ^]^ The spatial overlap of *lncBART*s, BRD4, CTCF, and EBNA1 at the *ori*P region suggests that these factors orchestrate EBV episome maintenance by modulating chromatin states. How *lncBART*s may interact with EBNA1, together with BRD4, CTCF and host origin recognition complexes to facilitate EBV genome replication requires further studies. Binding of the *lncBART*s‐BRD4/CTCF complex near the *ori*P region may generate a genome looping effect and serve as an enhancer for transcription from other latent promoter regions such as Qp, the promoter for EBNA1 expression in NPC and EBVaGC cells. In this study, enrichment of *lncBART*s‐BRD4/CTCF and EBNA1 was also observed in the Qp region and right‐most regions of the EBV genome (Figure 5g). Additional studies are needed to gain a better understanding of the role of *lncBART*s in EBV latent replication in NPC and EBVaGC cells.

EBV is a human tumor virus, and while the presence of EBV was shown to cause epigenome reprogramming in NPC cells, no specific viral function was characterized in this earlier study.^[^ ^55^ ^]^ In latency III LCLs, EBNA2, together with other EBNAs and LMP1, regulate EBV latency through modulation of EBV latent promoters and reprograms host gene expression to achieve immortalization of B cells.^[^ ^44^, ^56^ ^]^ How EBV contributes to the latency I (Burkitt's lymphoma) and II (NPC & EBVaGC) oncogenesis is not fully understood. We found that one of the targets of *lncBART*s regulation is *MYC*, which is also a target of EBNA2 in EBV‐immortalized LCLs.^[^ ^44^ ^]^ This finding reaffirms our hypothesis that *lncBART*s may mimic some functions performed by EBNA2 in the immortalization process of LCLs in their role in EBV‐associated epithelial cancers. It is notable that expression from *MYC* and *BCL2* is also subject to regulation by BRD4 in NPC cells. While the role of epithelial cells in EBV's lifelong persistence in vivo is still under debate,^[^ ^57^ ^]^ latent EBV infection in epithelial cells is certainly associated with the oncogenic process. In the CARPID analysis, besides BRD4 and CTCF, both characterized in this study, FOXM1 emerged as the most prominent interactor of *lncBART*s among the proteins associated with mitosis. FOXM1 is known as a cofactor to facilitate NF‐κB binding to DNA sites in LCL cells and coactivates the expression of key targets such as Tak1 and cIAP2.^[^ ^58^, ^59^ ^]^ Knockdown of FOXM1 markedly reduces NF‐κB target gene expression and induces rapid apoptosis in these cells, underscoring its importance in cell survival. However, there are no studies indicating that FOXM1 directly influence viral replication. The marked enrichment of FOXM1 suggests it may exert significant effects through alternative mechanisms, potentially involving NF‐κB regulation in EBV‐associated cancers, which warrants further investigation.

**Figure 7:**
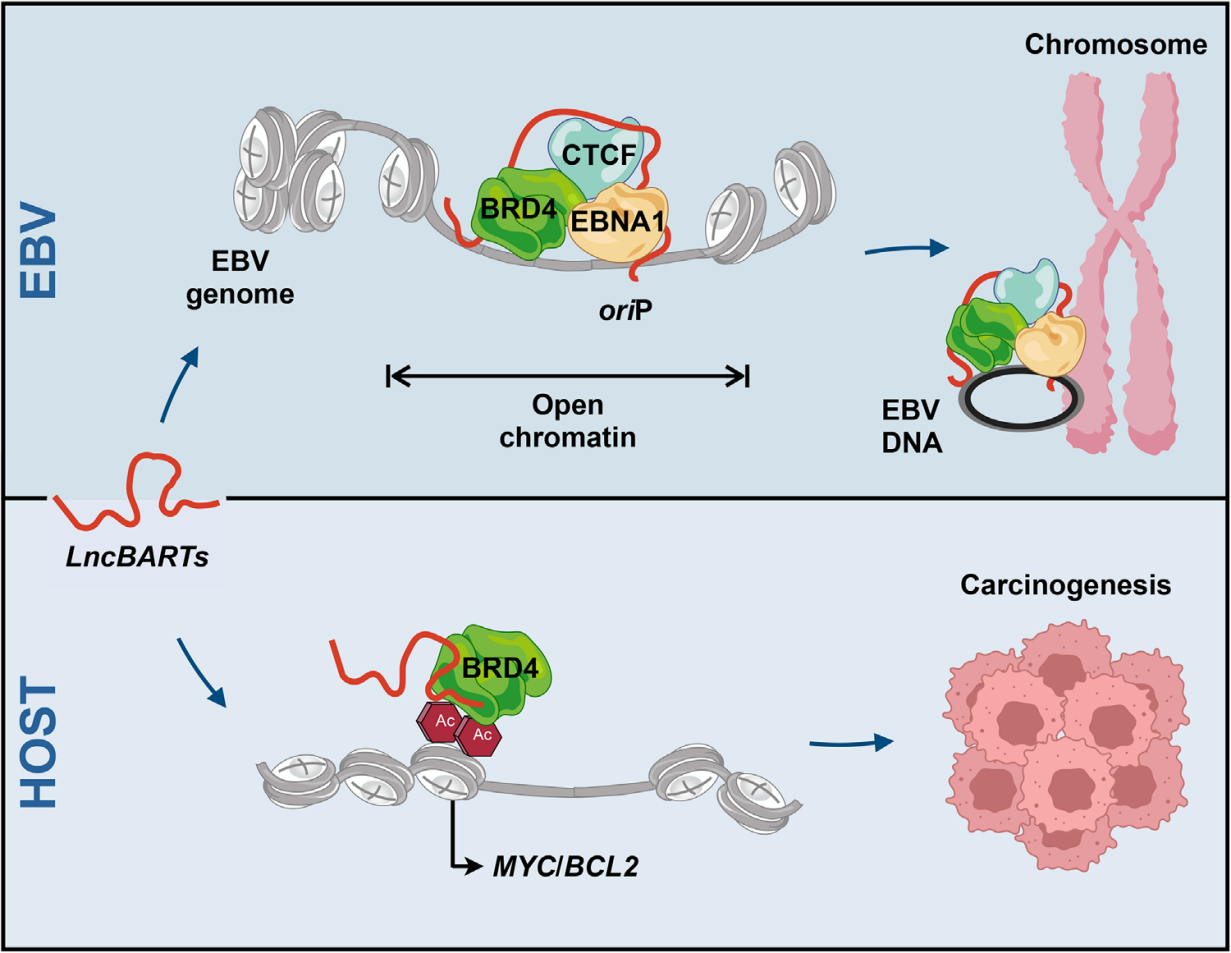
Working model of the *lncBART*s complex in maintaining EBV episome and promoting EBV‐associated oncogenesis. This working model illustrates that *lncBART*s anchor a protein complex, including host factors BRD4 and CTCF along with the viral factor EBNA1, to the EBV *ori*P region. This process is essential in tethering EBV to host chromosomes, thereby maintaining the EBV copy number in EBV‐infected cells. Besides, *lncBART*s affect the expression of host proto‐oncogenes, such as *MYC*, thereby leading to EBV‐associated oncogenesis.

In summary, to evade host immune surveillance, EBV enters EBNA2‐free latency, expressing only essential viral proteins (EBNA1) while utilizing virally encoded *lncBART*s to facilitate viral episomal replication in NPC and EBVaGC cells through their interaction with BRD4‐ and CTCF‐containing regulatory complexes. The interaction between *lncBART*s, BRD4, and CTCF leads to the reprogramming of the host epigenome and alteration of gene expression. This results in the aberrant expression of genes associated with growth and survival pathways, such as *MYC* and *BCL2*, ultimately driving EBV‐associated oncogenesis in these cells (**Figure** **7**).

This study is the first attempt to elucidate the epigenetic role of *lncBART*s in maintaining viral genome replication. Several issues remained to be further investigated. Although the BRD4 binding motif on *lncBART*s was identified via spyCLIP and validated with mutant RNA pulldown assays, these biochemical findings do not establish causality within a cellular context. Future studies involving re‐expression of an shRNA‐resistant, mutated *lncBART*s designed to prevent BRD4 binding are necessary to confirm its role in EBV episome maintenance. Off‐target effects are also a concern in the experiment utilizing various RNA knockdown approaches. While the doxycycline‐inducible shRNA system effectively reduced *lncBART*s levels with minimal off‐target effects supported by RNA‐seq analysis in this study, studies employing additional independent shRNAs are needed to exclude off‐target effects.

## 4 Experimental Section

### Human Tissue Samples

NPC biopsies and nasopharynx‐gectomized tissues used in this study were from patients admitted to Zhongshan City People's Hospital, Guangdong, P.R. China. The collection and use of these NPC specimens for this experimental study were approved by the Hong Kong Clinical Research Ethics Committee (HKECREC‐2021‐078), and the patients’ consents were obtained.

### Cell Lines

The EBV‐positive C666‐1, NPC43‐C7/NPC43‐C7‐M81, YCCEL1 and Namalwa cell lines were cultured in RPMI‐1640 medium, supplemented with 10% FBS, 100 U mL^−1^ penicillin, and 100 µg mL^−1^ streptomycin. The hTERT immortalized NP epithelial cell line NP460‐hTERT‐EBV and NP361‐hTERT‐EBV were kept in Defined Keratinocyte‐SFM (Thermo Fisher Scientific) and Epilife (Thermo Fisher Scientific) media mixed at a 1:1 ratio with 10% FBS, 100 U mL^−1^ penicillin, and 100 µg mL^−1^ streptomycin. The EBV‐infected gastric carcinoma cell line AGS‐BX1 was cultured in F‐12K Nutrient mixture (Invitrogen) supplemented with 10% FBS, 100 U mL^−1^ penicillin, and 100 µg/ml streptomycin. The YCCEL1 cell line was generously provided by Professor Qian Tao (Chinese University of Hong Kong), while the NPC43‐C7‐M81 and Namalwa cell lines were kindly supplied by Professors George Sai Wah Tsao and Wenwei Tu (The University of Hong Kong), respectively. These cells were maintained at 37 °C with 5% CO_2_ in humidified air (Table S1, Supporting Information).

### Dox‐Inducible shRNA Cell Line Construction

To create a dox‐inducible knockdown system, shRNA sequences targeting *lncBART*s or BRD4 were inserted into Tet‐pLKO‐puro vectors. To generate lentiviral particles, individual Tet‐pLKO‐puro vectors were co‐transfected with psPAX2 and pMD2.G into Lenti‐X 293T cells, and the supernatant collected at 48 h post‐transfection. To generate stable *lncBART*s knockdown cell lines, C666‐1, YCCEL1 or NPC43‐C7‐M81 cells were infected with lentiviral particles containing 8 mg/ml polybrene for 48 h and selected with puromycin at a final concentration of 2 mg mL^−1^ for five additional days. Cells were treated with dox (Thermo Fisher Scientific) at a final concentration of 2 mg mL^−1^ to induce the transcription of shRNAs and cleavage of endogenous *lncBART*s or BRD4. The dox‐containing medium was changed every 24 h to maintain the activity of the inducible promoter. The primers applied in this process are listed in Table S1 (Supporting Information).

### RNA‐seq

A NucleoSpin RNA kit (Macherey Nagel, Cat. No 740 955.50) was used to extract total RNA. Three biological replicates of each of *lncBART*s knockdown and control conditions were included in this study. Total RNA was processed for ribosomal RNA depletion with acceptable integrity (RIN). Sequencing and library preparation were performed by the Centre for PanorOmic Sciences (The University of Hong Kong) on the Illumina NovaSeq 6000 platform using the paired‐end 150bp approach. The quality of the raw data was evaluated via FastQC, and alignment to the reference human (hg38) and EBV (GenBank accession number NC_0 07605.1) genomes was performed by the STAR program. The quality of the BAM files, including the percentage of mRNA bases and ribosomal bases, was evaluated by Picard. The raw read counts for each gene were estimated by HTSeq. Differential expressed genes were identified by DESeq2 using the conditional comparison with a false discovery rate (FDR) *q*‐value <0.05 and Log2fold (L2FC) change ≥ 2.

### Quantification of EBV Copy Number

The experimental method refers to the report by Prof. George Sai Wah Tsao's group.^[^ ^60^ ^]^ DNA from cell lines was isolated using the DNeasy Blood&Tissue Kit (Qiagen, Cat. No 69 504). PCR amplification was conducted using a LightCycler 480 instrument (Roche). The primers and probes for *BALF5* and *β‐globin* were designed based on a protocol provided by Professor George Sai Wah Tsao's group (Table S1, Supporting Information). Each PCR reaction mixture contained 2 µl DNA (25 ng µL^−1^), 0.4 µL forward and reverse *BALF5* primer (10 µM), 0.2 µl forward and reverse *β‐globin* primer (10 µM), 0.2 µL *BALF5* probe with FAM (10 µM), 0.1 µL *β‐globin* probe with VIC (10 µM), 5 µL 2X TaqMan Universal PCR Master Mix (Thermo Fisher Scientific), and 2.1 µL PCR‐grade water. The reaction commenced with a 10 min pre‐incubation at 95 °C. Forty amplification cycles were performed, involving DNA denaturation at 95 °C for 15 s and annealing and elongation at 60 °C for 60 s. Serially diluted DNA from Namalwa Burkitt Lymphoma cells, which contain two integrated EBV genomes per cell, was used in the PCR process to create individual calibration curves for *BALF5* and *β‐globin*. The average EBV copy number per cell was calculated using the standard calibration curves generated from Namalwa DNA.

### EBV DNA FISH

The experimental method used in this study is based on the 2018 report by Sai Wah Tsao's group.^[^ ^60^ ^]^ Cells were harvested and treated with 2 mL of 0.8% sodium citrate for 15 min at 37 °C, followed by addition of 20 µL of a 1:3 acetic acid/methanol (fixative solution) for 5 min at 37 °C. The cells were then centrifuged at 115×*g* for 5 min at room temperature (RT), and the supernatant discarded. Cells were then resuspended in 5 mL of fixative solution and centrifuged again at 115×*g* for 5 min. The supernatant was removed, and another 5 mL of fixative solution was added. This washing process was repeated three times before the cells were spread onto a slide and air‐dried. The slide was then aged at RT for 5‐7 days. For FISH analysis, the aged slide was treated with 0.1 mg mL^−1^ RNase A for 1 h at 37 °C, 2× SSC (0.30 m sodium chloride, 0.03 m sodium citrate) for 10 min at RT, 0.015 µg mL^−1^ proteinase K for 30 min at 37 °C, then fixed with 3% paraformaldehyde for 10 min at RT, and washed with 2× SSC for 10 min at RT. The slide was then dehydrated using 70%, 85%, and 95% ethanol sequentially for 2 min each at RT and air‐dried. A biotinylated EBV DNA probe (Enzo, Cat. No 40 836), which targeting the 2.68 Bam HI “V” region (the internal repeat) of the EBV genome, was dissolved in hybridization solution (Enzo) at a ratio of 1:20, denatured for 5 min at 80 °C, and incubated for 30 min at 37 °C. The slide was placed in a denaturing solution (70% formamide dissolved in 20× SSC) for 4 min at 80 °C, progressively dehydrated with 70%, 85%, and 95% ethanol for 2 min each at RT, and air‐dried. The probe was then added to the slide, covered with a coverslip, and sealed with rubber cement. The slide was incubated overnight at 37 °C in a humidified chamber, the coverslip then removed, and the slide washed twice with 50% formamide for 5 min at 45 °C, and then twice with 2× SSC for 5 min at 45 °C. Fluor‐conjugated streptavidin (Thermo Fisher Scientific) was added to the slide and incubated at RT for 40 min. The slide was then washed twice with 2× SSC for 5 min at 45 °C, dehydrated with 70%, 85%, and 95% ethanol sequentially for 2 min each at RT, and air‐dried in nitrogen gas. DAPI (4′,6‐diamidino‐2‐phenylindole) was added to the slide for DNA staining, and a coverslip was applied. Fluorescence images were captured using a Carl Zeiss LSM 980 confocal microscope and analyzed using ZEN microscope software (Zeiss).

### RT‐qPCR

The PrimeScript RT Reagent Kit with gDNA Eraser (Takara Bio, Cat. No RR037B) was utilized to convert RNA into complementary DNA (cDNA), while removing genomic DNA in the initial step. The reverse transcription process took 15 min, using Oligo dT Primer and random 6‐mers. The resulting cDNA was diluted 1:20 with RNase‐free water to serve as templates. qPCR reactions were set up using TB Green Premix Ex Taq (Takara Bio, Cat. No RR82WR) and executed in a LightCycler 480 instrument. The cycle thresholds (Ct) values were assessed using the software included with the instrument, employing the 2nd derivative max method. The relative gene expression was determined using the 2^−ΔΔCT^ method. The primers applied in this process are listed in Table S1 (Supporting Information).

### RNA FISH/IFA

Quasar 570‐conjugated probe sets for *lncBARTs* were designed using the Stellaris Probe Designer and synthesized by Biosearch Technologies. FISH was carried out following the manufacturer's guidelines. Briefly, cells were cultured on 12 mm round cover glasses (Carolina) in a 24‐well cell culture plate coated with Poly‐L‐Lysine Solution (Santa Cruz Biotechnology). Cells on the cover glasses were fixed with 4% PFA and permeabilized using 0.25% Triton‐X. After washing with FISH washing buffer (10% formamide and 10% SSC in H_2_O), the cells on cover glasses were incubated with Stellaris RNA probes and primary antibodies targeting the protein of interest (Table S1, Supporting Information), diluted 1:100 in Hybridization Buffer (comprising 90% Stellaris RNA FISH Hybridization Buffer from Biosearch Technologies and 10% deionized formamide), at 37 °C for 6 to 16 h in the dark. The cover glasses were then washed with FISH washing buffer (Biosearch Technologies) and incubated with the appropriate secondary antibody at RT for 1 h in the dark. After several washes with FISH washing buffer, the cover glasses were mounted with Vectashield Mounting Medium, positioning the cells downwards, onto microscope slides. Images were captured using a Carl Zeiss LSM 980 confocal microscope and analyzed using ZEN software.

### CARPID

This part of the study was conducted in collaboration with Professor Jian Yan ‘s group at City University of Hong Kong, the developers of CARPID.^[^ ^37^ ^]^ The dCasRx plasmid was fused with the modified biotin ligase BASU and co‐transfected into C666‐1 cells along with gRNA targeting specific regions of *lncBART*s. Cells transfected with an empty gRNA‐expressing vector served as the control. Biotin was added to the culture medium and incubated with the transfected cells, allowing proteins bound to *lncBART*s to be biotin‐labeled. The specificity of the gRNA was confirmed by anti‐biotin immunoprecipitated RNA, which showed a significant enrichment of *lncBART*s targeted by gRNA compared to the control gRNA. Cells were collected and lysed in 1 ml lysis buffer (50 mM Tris‐HCl (pH 7.4), 150 mM NaCl, 0.5% Triton X‐100, 1 mM EDTA supplemented with fresh protease inhibitors (Roche)) at 4 °C for 10 min with rotation. The enriched biotinylated proteins were immunoprecipitated by incubation MyOne streptavidin T1 beads (Thermo Fisher) for 2 h at 4 °C with rotation and then washes three times 1 ml ice‐cold lysis buffer. For on‐bead digestion, enriched streptavidin beads were washed three times with 50 mM ammonium bicarbonate (pH 8.0) (Sigma) at 4 °C, then resuspended in 50 µL elution buffer I (50 mM Tris‐HCl pH 8.0; 2 m urea, 10 µg mL^−1^ sequencing‐grade trypsin (Thermo Fisher, 1 mM DTT) and mixed at 400 rpm.at 30 °C, for 60 min. The supernatant was collected in a fresh vial, with the beads being eluted twice more with 25 µL elution buffer II (50 mM Tris‐HCl pH 8.0, 2 m urea, 5 mM iodoacetamide), and all 3 eluates then were combined. An additional 0.25 µg trypsin was added to the combined eluates, followed by incubation at 37 °C overnight. The digestion was quenched by adding 10% formic‐acid solution (FA) at a ratio of 1:25 (vol/vol). The digested samples were then desalted using C18 tips (Thermo Fisher), following the manufacturer's instructions, and reconstituted in 20 µL 0.1% FA. The liquid chromatography‐tandem MS (LC‐MS/MS) analysis was performed using an Short Gradient LC‐MS/MS Run using timsTOF Pro system.

Each protein needed to be identified by more than two unique peptides. The comparative binding analysis between the *lncBART*s binding protein and the control was conducted using DESeq2. The results were considered significant if the absolute log2 fold change ≥ 2 and *q*‐value < 0.05.

### Western Blotting

Cells were lysed in a buffer solution (150 mM NaCl, 1% TritonX‐100, protease inhibitor cocktail from Roche, 50 mM Tris‐HCl pH 7.4) at 4 °C for 30 min while rotating. Centrifugation was used to remove cell debris, and the supernatant collected and combined with a 6x loading buffer (0.35 m Tris‐HCl pH 6.8, 10% SDS, 30% glycerol, 0.6 M DTT, 0.12% Bromophenol blue). After heating at 95 °C for 10 min, the samples were prepared for sodium dodecyl sulfate‐polyacrylamide gel electrophoresis (SDS‐PAGE). The separation gel percentages were determined based on target protein sizes, and the running current was set to 30 A per gel. The separated proteins were then transferred from the gel to a nitrocellulose membrane (Bio‐Rad) at 120 V. The membranes were blocked with 3% skim milk (Sigma‐Aldrich) in PBS at RT for ≈30 min and incubated with primary antibodies diluted in blocking buffer (2.5% BSA and 0.05% NaN_3_ in PBS) overnight at 4 °C. The primary antibodies used are listed in Table S1 (Supporting Information). Following this, the membranes were washed with PBST (0.2% Tween‐20 in PBS) and exposed to IR‐Dye labelled secondary antibodies (Li‐Cor Bioscience) in the dark for 1 h at RT. The membranes were then washed with PBST, dried, and imaged using the Odyssey imaging system (Li‐Cor Bioscience).

### Cco‐IP

Cells were collected and lysed in lysis buffer (as for western blot) at 4 °C for 30 min. Meanwhile, the corresponding antibodies were combined with either Dynabeads Protein A or G (Thermo Fisher Scientific) and incubated at RT for 10 min. 10% of the supernatant was reserved as input, while the rest was mixed with pre‐washed Anti‐FLAG M2 Magnetic Beads (Sigma‐Aldrich) or antibodies (pre‐attached to protein A/G Dynabeads) and incubated at 4 °C overnight. As a control, IgG was bound to Dynabeads Protein A or G. After incubation, the bead complexes were washed five times with lysis buffer, resuspended in SDS loading buffer, and heated at 95 °C for 10 min. The samples were then analyzed by western blot.

### RIP

RIP of *lncBART*s interaction with BRD4 was performed using the Magna RIP Kit (MILLIPORE, Cat. No 17‐700) following the manufacturer's protocol. All necessary reagents were provided in the kit. Briefly, cells were lysed in RIP lysis buffer and incubated on ice for 5 min. 10% of the RIP lysate was reserved as input and stored at −80 °C until RNA purification. Antibodies for proteins of interest and isotype control were incubated with magnetic beads while rotating for 30 min at RT. Immunoprecipitation of protein‐RNA complexes was carried out in RIP immunoprecipitation buffer by mixing the RIP lysate and beads with antibodies, followed by overnight incubation with rotation at 4 °C. After incubation, the beads were washed five times with cold RIP wash buffer and treated with proteinase K to digest any remaining proteins. The RNA was then purified using phenol:chloroform:isoamyl alcohol and precipitated with glycogen (Thermo Fisher Scientific, Cat. No R0551) in absolute ethanol. Finally, the RNA was analyzed by RT‐qPCR.

### RNA Pulldown

Biotinylated RNAs were incubated overnight at 4 °C with BRD4 1‐444, BRD4 1‐722, BRD4 1047‐1362, and BRD4 1224‐1362 proteins, which were purified from bacteria. M‐280 Streptavidin beads (Thermo Fisher Scientific, Cat. No 11205D) were washed sequentially with buffer A (0.1 m NaOH and 0.05 m NaCl) and buffer B (0.1 m NaCl) to inactivate potential RNases before adding to the RNA‐protein mixture. After incubation at 4 °C for 4 h, the beads were washed with lysis buffer (as for western blot) containing 0.05 U µL^−1^ RNaseOUT Recombinant Ribonuclease Inhibitor (Thermo Fisher Scientific, Cat. No 10 777 019), resuspended in 2× SDS sample, boiled at 95 °C for 10 min and then analyzed by western blot.

### IVT

RNA was synthesized from a DNA template containing a T7 promoter using the HiScribe T7 High Yield RNA Synthesis Kit (New England Biolabs, Cat. No K0441). Briefly, T7 RNA polymerase mix, reaction buffer, ATP, GTP, CTP and UTP provided in the kit were mixed and incubated with biotin labelled‐11‐dUTP (Thermo Fisher, Cat. No R0081) and template DNA at 37 °C for 2‐4 h. The RNA from IVT was purified using NucleoSpin RNA Clean‐up kits (Macherey Nagel). The biotinylated RNA was heated to 65 °C for 10 min and slowly cooled down to 4 °C to allow proper RNA folding. The purified RNA was stored at −80 °C for future use. The RNA quality was assessed using the Agilent 2100 Bioanalyzer at the Centre for PanorOmic Sciences.

### SpyCLIP

The genome‐wide BRD4‐RNA interactions were identified using SpyCLIP‐seq.^[^ ^61^ ^]^ The FLAG/Spy‐tagged BRD4‐overexpressing C666‐1 cells were irradiated at 400 mJ cm^−2^ using a UV Crosslinker. In brief, cells were lysed, DNA was removed by Turbo DNase (Invitrogen, Cat. No AM2238), and RNA was then fragmented using 1:200 diluted RNase I (Invitrogen, Cat. No AM2295). Mixed lysates were incubated with Anti‐FLAG M2 Magnetic Beads (Sigma‐Aldrich, Cat. No F3165) for 1 h at 25 °C. After stringent washing, beads were digested with proteinase K (Roche, Cat. No 3 115 828 001) RNA was purified using an RNeasy MinElute Cleanup Kit (Qiagen, Cat. No 74 204), and libraries were constructed using the NEBNext Ultra RNA Library Prep Kit for Illumina (New England Biolabs, Cat. No E7770). Sequencing was performed on the Illumina NovaSeq 6000 platform using the paired‐end 150bp method (Novogene).

The read quality was checked by FastQC. FLEXBAR was used to trim adaptor sequences and STAR for with reference human (hg38) and EBV (GenBank accession number NC_0 07605.1) genomes. Picard was used to remove PCR duplicates. FindMotifsGenome from HOMER and STREME were used to identify the consensus motif of *lncBART*s with *p*‐values <0.05.

### ATAC‐seq

ATAC‐seq was performed on biological replicates of samples following the method described by Buenrostro et al.^[^ ^62^ ^]^ In brief, cells (50 000) were lysed in cold lysis buffer (10 mm Tris‐HCl pH 7.4, 10 mm NaCl, 3 mm MgCl_2_, 0.1% NP‐40). Cell nuclei were collected by quickly centrifuging the cell lysates. Tagment DNA Enzyme 1 (Illumina, Cat. No E7645S) was used for transposition according to the product instructions. The transposed DNA was purified using a Qiagen MinElute Kit (Cat. No 28 004). The transposed DNA fragments were amplified by PCR reaction by adding NEBNext High‐Fidelity 2× PCR Master Mix (New England Biolabs, Cat. No M0541S), SYBR Green I (Invitrogen, Cat. No S7563), and primers. The number of PCR cycles was determined by quantitative PCR using a LightCycler 480 instrument. The libraries were sequenced by the Centre for PanorOmic Sciences on the Illumina NovaSeq 6000 platform using the paired‐end 150bp approach.

Data analysis was performed following the ENCODE ATAC‐Seq pipeline (v1.0), which includes alignment to the reference human (hg38) and EBV (GenBank accession number NC_0 07605.1) genomes, peak calling, and quality evaluation. The narrowpeaks files were summarized, and BAM files were used to generate the raw count at the summarized peak regions. Differential peak analysis was performed using DESeq2 to identify the differentially accessible regions with the absolute log_2_ fold change ≥2 and q‐value <0.01 being considered significant.

### ChIRP‐seq

ChIRP of *lncBART*s was conducted using the Magna ChIRP RNA Interactome Kit (MILLIPORE, Cat. No 17‐10494) following the manufacturer's protocol. The experiment was performed in triplicate to evaluate the reproducibility of the ChIRP‐seq data. Briefly, cells (50 000 000) were harvested and lysed in Lysis Buffer containing 5 µL of 200X Protease Inhibitor Cocktail and 5 uL of RNase inhibitor. Chromatin was sheared though a sonication (BioruptorPico UltraSonication System) at pulse intervals of 30 s ON, 30 s OFF, for 4 h. Sheared DNA chromatin materials from sonicated cells were hybridized with 100 pmol of biotinylated tiling DNA probes targeting *lncBART*s, which were designed using the online probe design program available at www.biosearchtech.com/support/tools/design‐software/chirp‐probe‐designer. The biotin‐labeled DNA chromatin was captured by adding 100 µL of Streptavidin‐magnetic beads. The *lncBART*s complexes were released by addition of Proteinase K (Cat. No CS207286). For each sample 100 ul of released *lncBART*s complexes were processed using QIAGEN miRNeasy Mini Kit (Cat. No 217 084) for RNA isolation, and 900 ul released *lncBART*s complexes for DNA isolation. Quantitative RT‐PCR analysis was performed on RNA‐isolated samples to confirm *lncBART*s retrieval. For sequencing, 1.5 µL RNase A (Part # 20‐297) and 1.5 µL RNase H (Part # CS216564) were used to isolate the genomic DNA. The libraries were generated and sequenced by the Centre for PanorOmic Sciences on the Illumina NovaSeq 6000 platform using the paired‐end 150bp method.

The ENCODE ChIRP‐seq pipeline has been followed. Trimmomatic was used to trim the adaptor sequences. Sequencing reads were aligned to human (hg38) and EBV (GenBank accession number NC_0 07605.1) genomes using Bowtie2. Aligned reads were sorted and PCR duplicates were removed by Picard. MACS2 was used for the peak calling at ‐q 0.05.

### CUT&RUN‐seq

The CUTANATM ChIC/CUT&RUN Kit (EpiCypher, Cat. No 14‐1048) was used to identify BRD4 and CTCF binding sites in *lncBART*s knockdown and control C666‐1 cells. Cells (500000) were harvested by trypsinization and concanavalin A‐coated (ConA) magnetic beads used to capture the cells. Using 5% digitonin to permeabilised the cell membranes allowedprimary antibodies against CTCF (Millipore, Cat. No 07‐729), BRD4 (EpiCypher, Cat. No 13‐2003), H3K4me3 (Epicypher, Cat. No 13‐0041), H3K27ac (Abcam, Cat. No ab4729) and IgG‐specific (Epicypher, Cat. No 13‐0042) secondary antibodies to bind to the DNA chromatin during incubation overnight at 4 °C. Enzyme cutting was initiated by adding 2 uL of CUTANA pAG‐MNase (Cat. No 15‐1016) for 10 min at room temperature and then incubating with 100 mM calcium chloride for 2 h at 4 °C. Enriched DNA was purified and prepared for sequencing library using the NEBNext Ultra DNA Library Prep Kit for Illumina (Cat. No E7370L). The libraries were sequenced with 20 million (150 bp paired‐end) sequencing reads using the NovaSeq 6000 platform at the Centre for PanorOmic Sciences.

Sequencing adaptors were trimmed by Trimmomatic. Bowtie2 was used to align reads to the human (hg38) and EBV (GenBank accession number NC_0 07605.1) genomes with the parameters ‐p 2 –dovetail –phred33. Aligned reads were sorted and PCR duplicates removed by Picard. MACS2 was used for the peak calling using matched control IgG at ‐q 0.05. The signal tracks were normalised by RPKM at binsize 1.

### Hi‐C

The Omni‐C library was prepared using the Omni‐C Kit (Dovetail, Cat. No 21 005) following the manufacturer's guidelines. In brief, 500 000 freshly harvested cells were lysed and the released chromatin fixed in the nucleus using disuccinimidyl glutarate (DSG) and formaldehyde. The cross‐linked chromatin was then digested in situ using DNase I. The digestion efficiency was measured by a bioanalyzer within the 100–2500 bp range. After digestion, cells were lysed with SDS to extract chromatin fragments. Chromatins were bound to Chromatin Capture Beads, repaired and ligated to a biotinylated bridge. Ligated chromatin underwent reverse crosslinking and DNA purification. Libraries were then constructed using the Dovetail Library Module for Illumina (Cat. No 25 004) and subsequently sequenced with 150 bp paired‐end reads on the NovaSeq 6000 platform at the Centre for PanorOmic Sciences.

The sequencing reads were aligned to the human (hg38) and the EBV (GenBank accession number NC_0 07605.1) genomes using the BWA‐MEM alignment method. Valid ligation events and the ligation junctions were recorded with –min‐mapq 40, –walks‐policy 5unique, and –max‐inter‐align‐gap 30. Aligned reads were sorted and PCR duplicates eliminated using SAMtools. Random sampling to normalise to an equal amount of 200 million paired reads has been applied to the *lncBART*s knockdown and control C666‐1 cells to ensure comparability. The top 5% highest confidence interchromosomal EBV‐DNA interactions were calculated, and a chi‐square test was individually performed to assess significant binding (*p*‐value ≤ 0.01).

### Single‐Cell Spatial Analysis with the CosMx Spatial Molecular Imager (SMI)

The SMI sequencing method uses in situ hybridization to link RNA probes with fluorescent barcodes. The analysis involved 1000+ RNA probes targeting *RPMS1* on FFPE slides obtain from Queen Elizabeth Hospital in Hong Kong. The RNA probes with fluorescent barcodes were in situ hybridised onto the slides and x, y, and z spatial positions registered. Nuclei were defined by DAPI staining and the raw data were processed and imaged via the AtoMx Spatial Information Platform. The raw counts were further evaluated, normalised and scaled through R package Seurat (v5). The UMAP method was used for dimensional reduction, and the epithelial clusters were identified based on the PanCK staining score and expression of cytokeratin genes. Only the epithelial cells with detectable expression of *MYC* and *RPMS1* were used for evaluating the co‐expression of two genes.

### IHC

The streptavidin‐biotin‐peroxidase complex method was used for IHC staining. Paraffin sections were deparaffinized and rehydrated through a series of xylene, 100% ethanol, 95% ethanol, and 70% ethanol. Microwave heating at 100 W for 20 min in 10mM sodium citrate (pH 6.0) was used for antigen retrieval. Specimens were then blocked with 3% H_2_O_2_ for 5 min and in blocking buffer (3%BSA in 0.1%PBST) for 1 h. Primary antibody was added and incubated at 4 °C overnight. EnVision Plus System‐HRP (DAB; DAKO) was applied to amplify the signal, followed by hematoxylin counterstaining and dehydration. Images were captured using a color imaging microscope.

### Quantification and Statistical Analysis

Error bars were derived from triplicate biological experiments (mean ± SD, n = 3). Statistical significance was determined using an unpaired two‐tail Student's *t*‐test. Data are presented as mean ± SEM. Significance levels are indicated as *p < 0.05, **p < 0.01, ***p < 0.001, ****p < 0.0001, with “ns” denoting no significance. All graphs and analyses were generated using Graphpad Prism version 10 and R version 4.3.2.

## Conflict of Interest

The authors declare no conflict of interest.

## Author Contributions

H.C. and J.L. designed the studies; J.L., D.L‐S.C., C.J.C., Y.L., S. H., W.Y., P.W., and B.W.‐Y.M performed the experiments; H.C., J.L., D.L‐S.C., M.J., L.K‐Y. C. J. Y., and W.D. analyzed the data; H.C. and J.L. wrote the paper.

## Supporting information



Supporting Information

Supporting Information

Supporting Information

Supporting Information

Supporting Information

Supporting table 1

Supporting table 2

## Data Availability

The data that support the findings of this study are available in the supplementary material of this article.
