# Supplementary material for: Epstein‐Barr Virus Expressed Long Non‐Coding RNA (lncBARTs) Regulate EBV Latent Genome Replication
Source: Adv Sci (Weinh). 2025 Nov 11;13(8):e07286. doi: 10.1002/advs.202507286 (PMC12884799; doi:10.1002/advs.202507286)
Supplement: Supplementary file 1 — Supporting Information [file ADVS-13-e07286-s004.pdf]

Fig.4

c

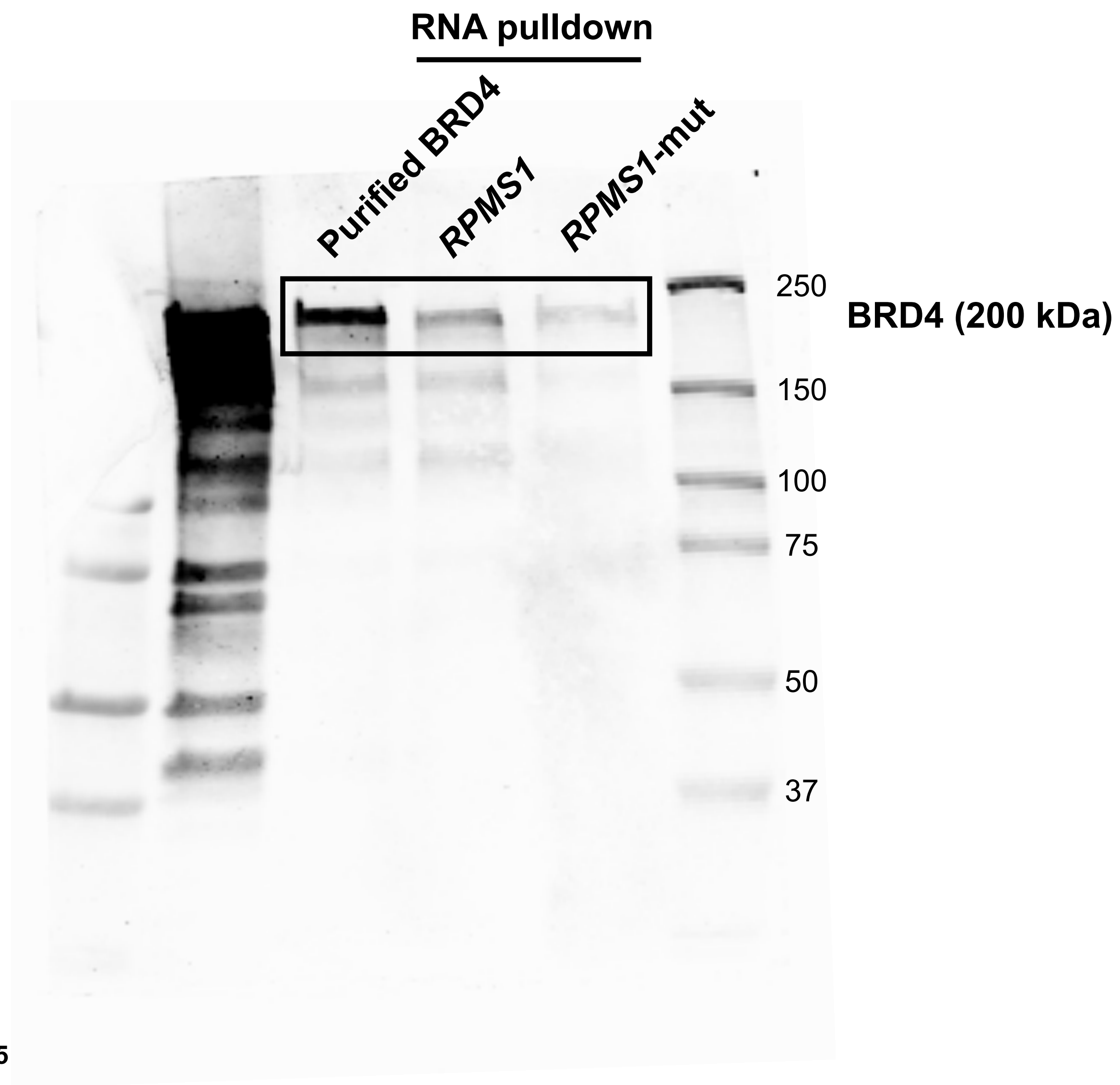

f

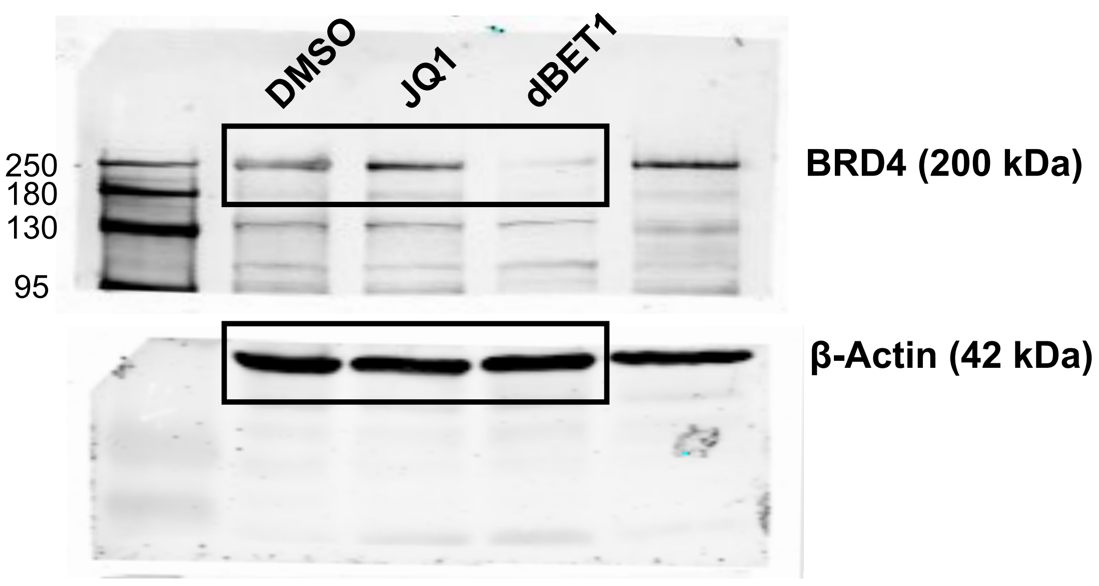

Fig.5

a

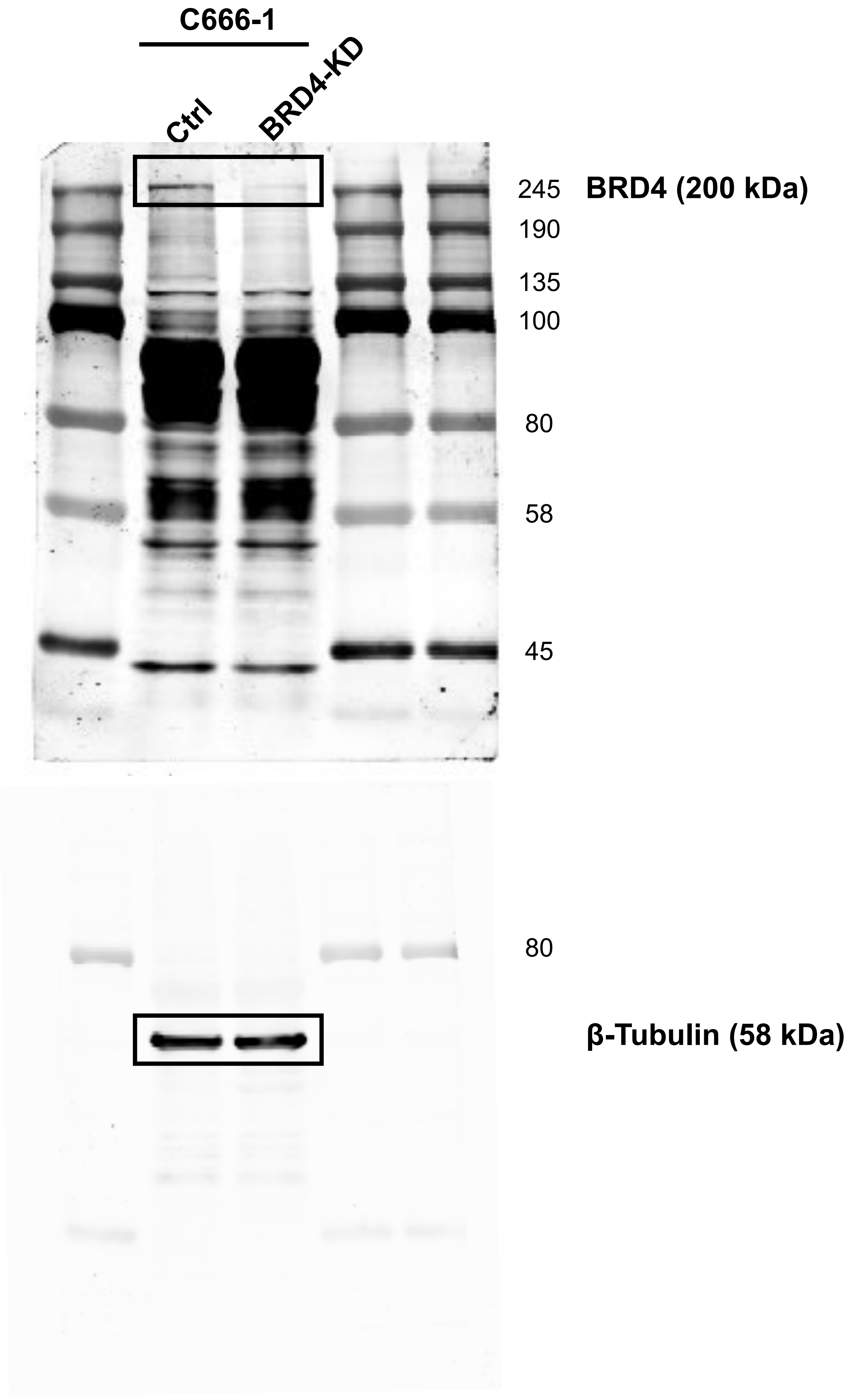

f

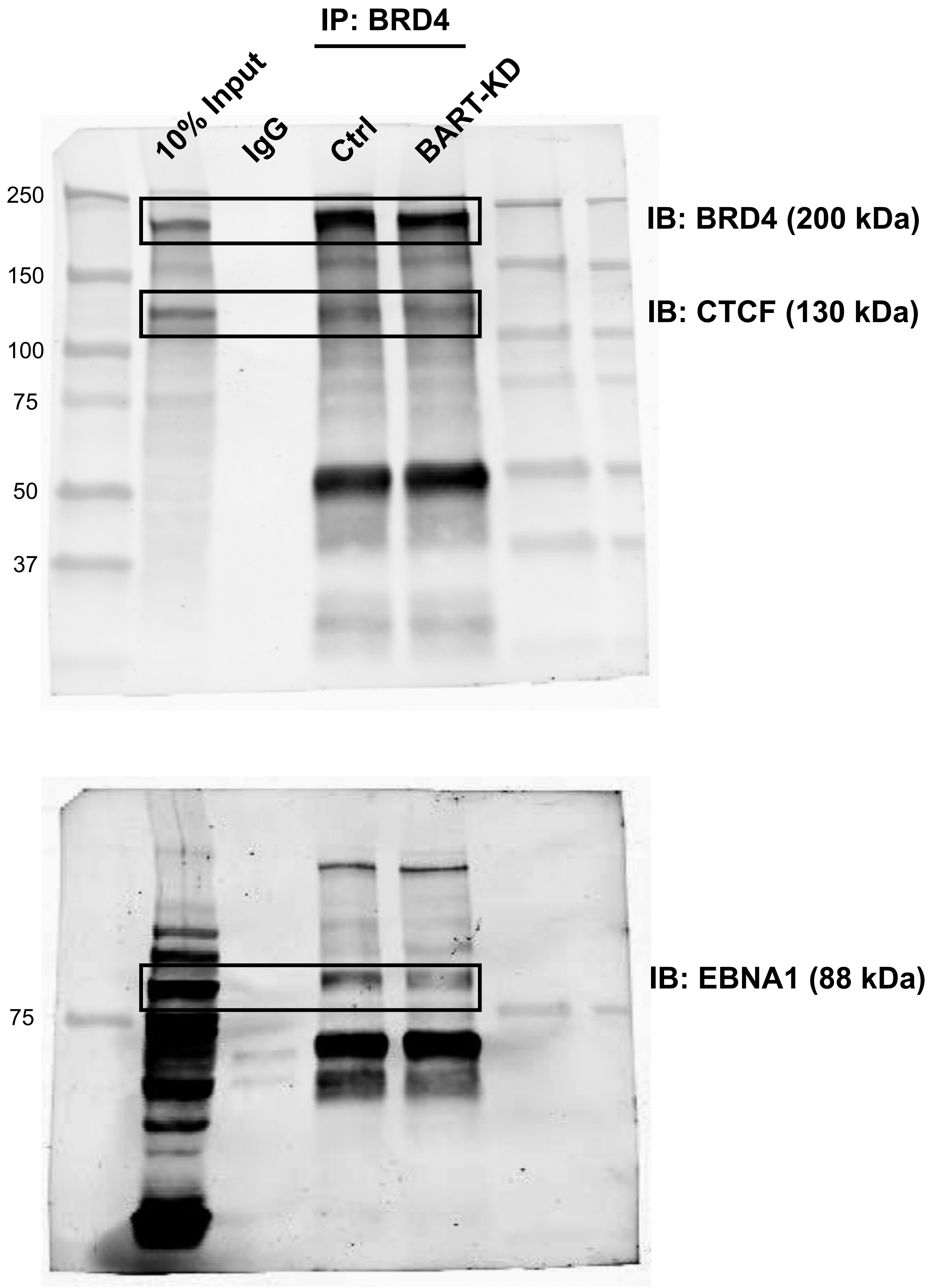

Fig.6
e

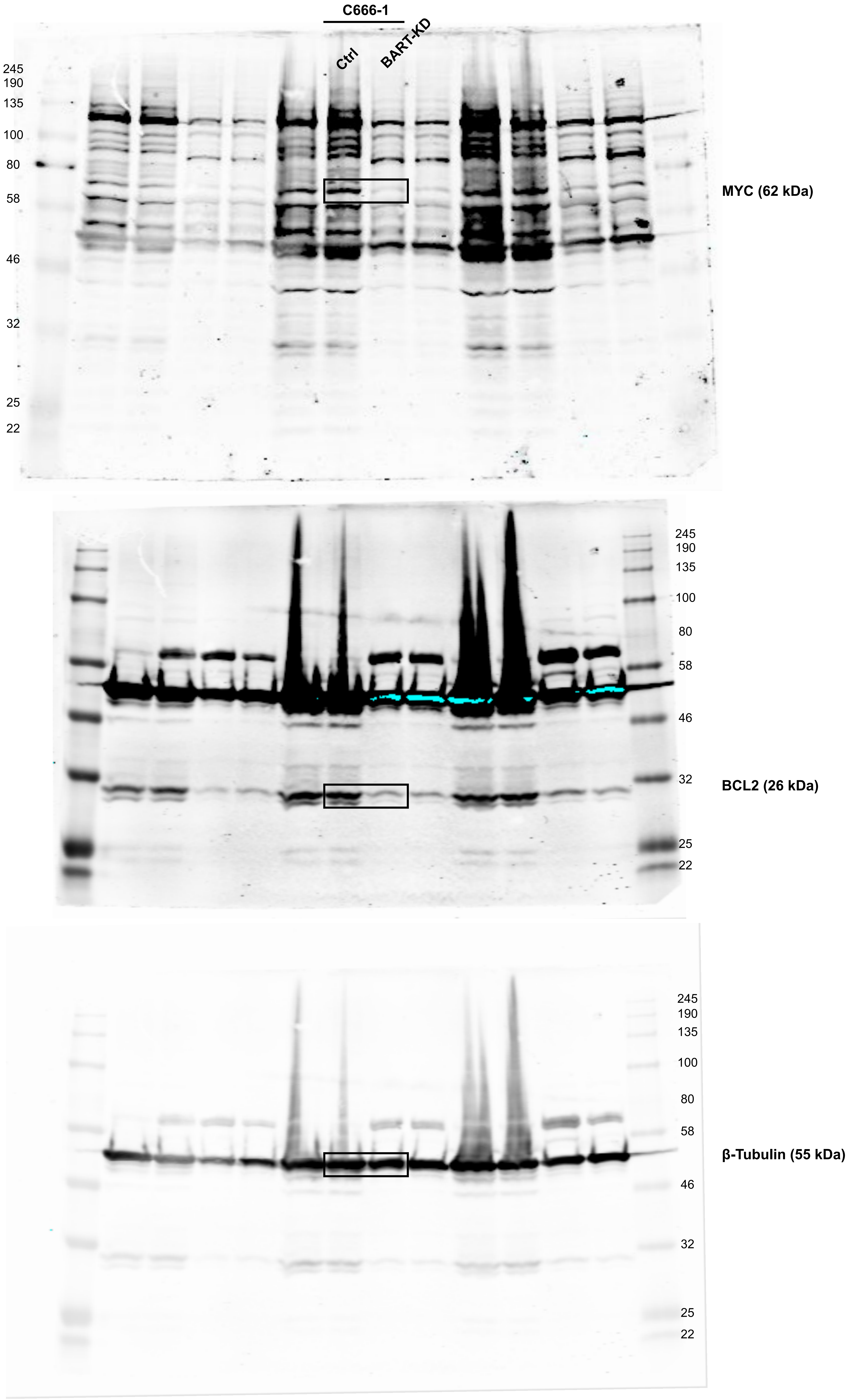

C666-1
Ctrl
BART-KD
245
190
135
100
80
58
46
32
25
22
MYC (62 kDa)
BCL2 (26 kDa)
β-Tubulin (55 kDa)

Fig.6
e

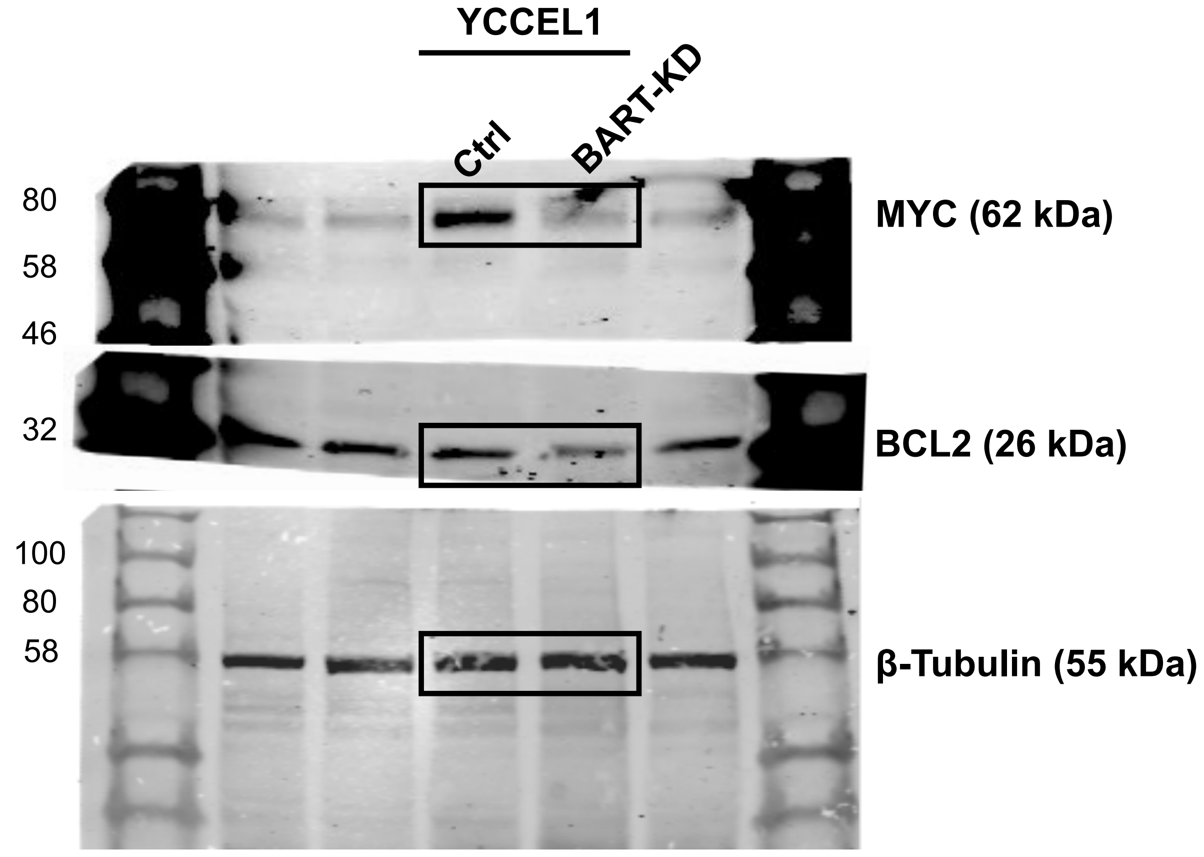

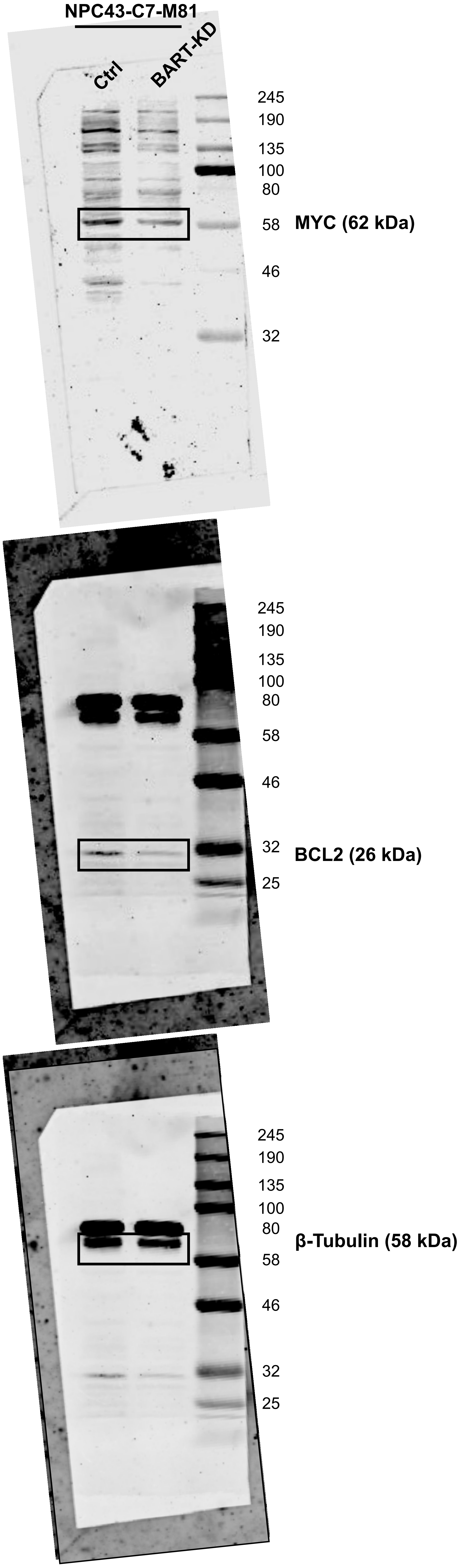

Note:

1. The original blot was stained simultaneously with BCL-2 and β-Tubulin antibodies. Images of BCL-2 and β-Tubulin were obtained from the same blot using different exposure times, respectively.

2. β-Tubulin blot shows two distinct bands in this blot, likely due to post-translational modifications (e.g., phosphorylation, glycosylation) of Tubulin proteins (Song Y, Brady ST. Post-translational modifications of tubulin: pathways to functional diversity of microtubules. Trends Cell Biol. 2015). We selected one of the β-Tubulin bands as the loading control.

Fig.6

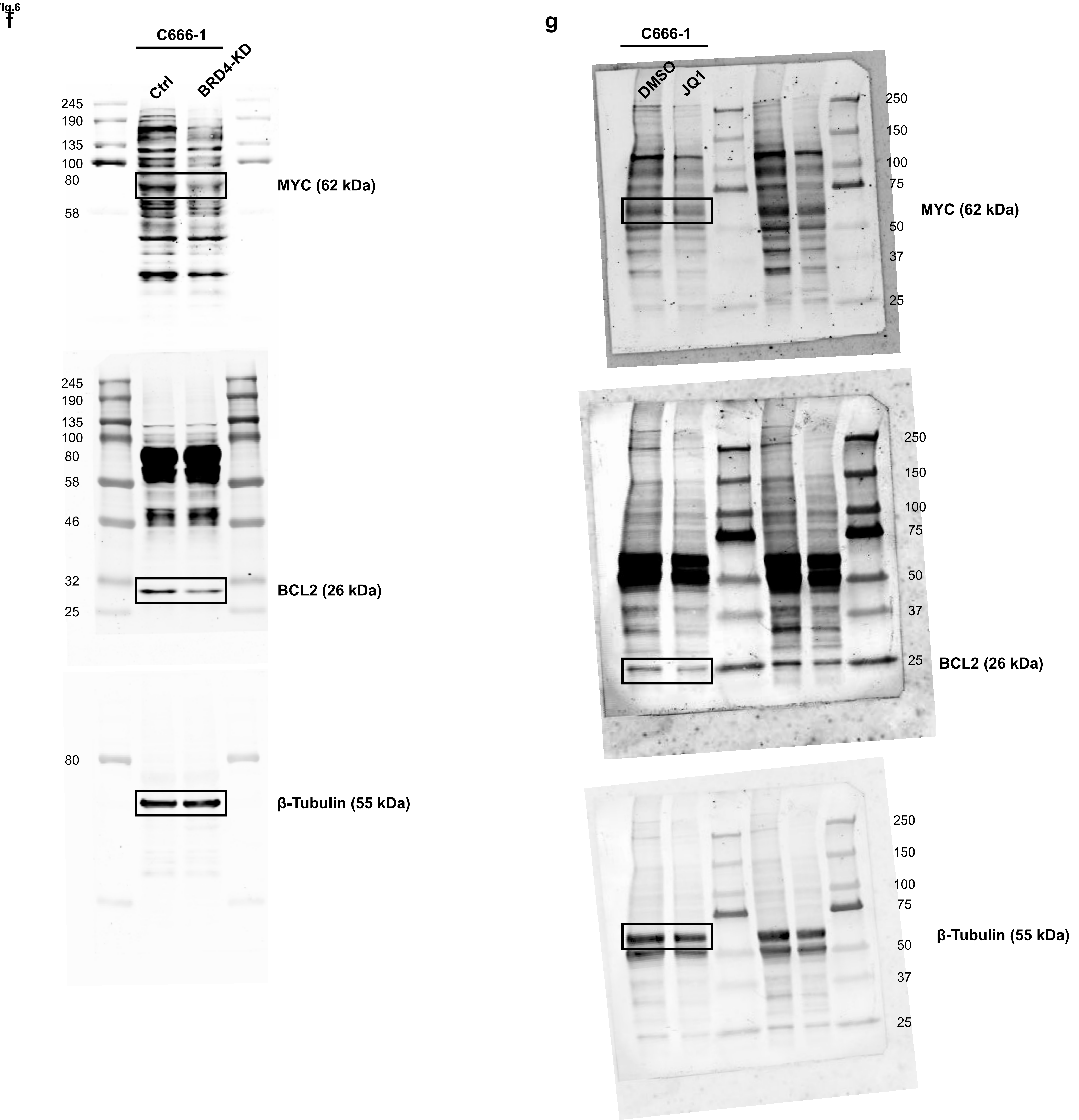
f
C666-1
Ctrl
BRD4-KD
245
190
135
100
80
58
MYC (62 kDa)
245
190
135
100
80
58
46
32
25
BCL2 (26 kDa)
80
β-Tubulin (55 kDa)
g
C666-1
DMSO
JQ1
250
150
100
75
50
37
25
MYC (62 kDa)
250
150
100
75
50
37
25
BCL2 (26 kDa)
250
150
100
75
50
37
25
β-Tubulin (55 kDa)

Fig.S2

h

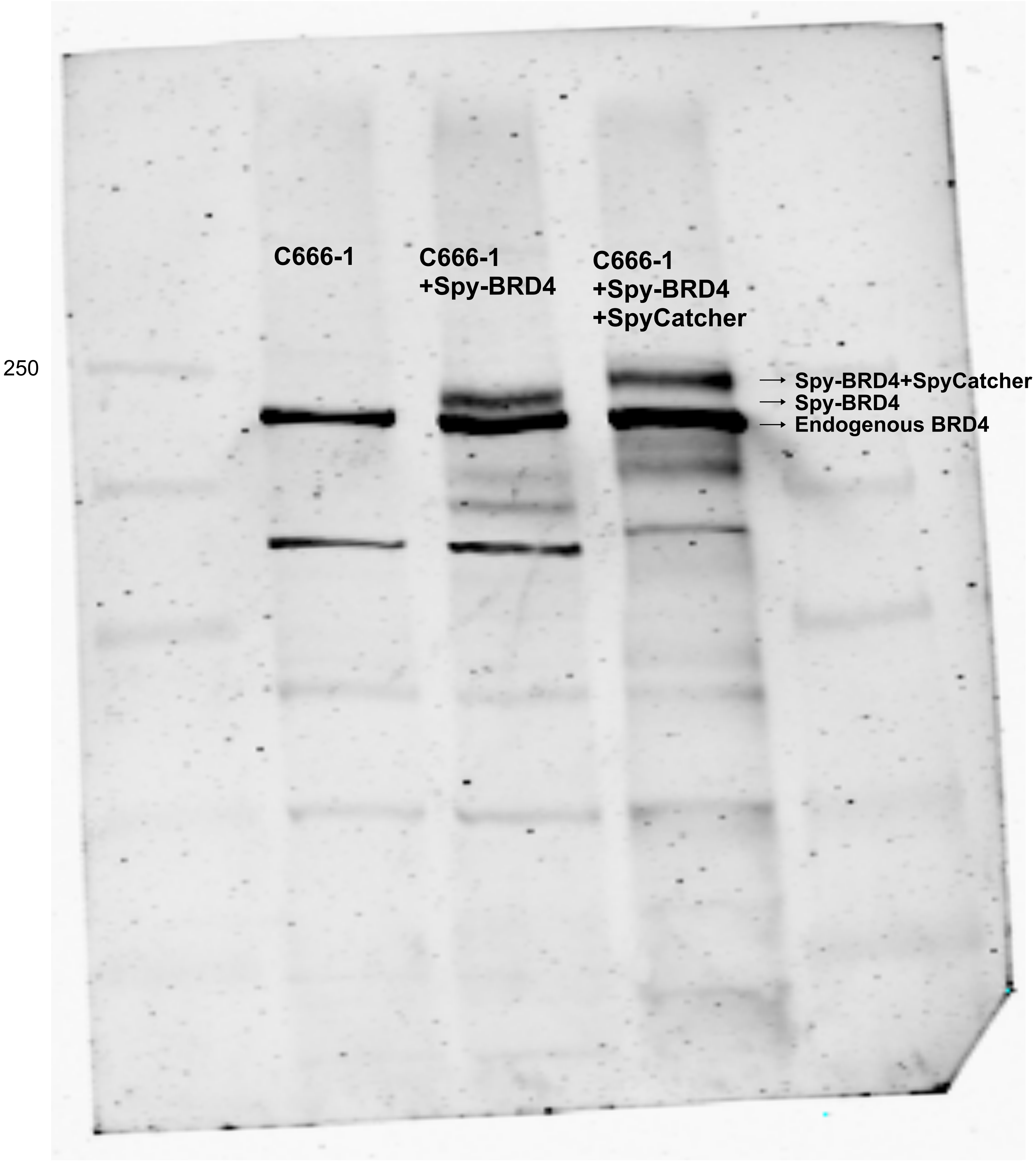

j

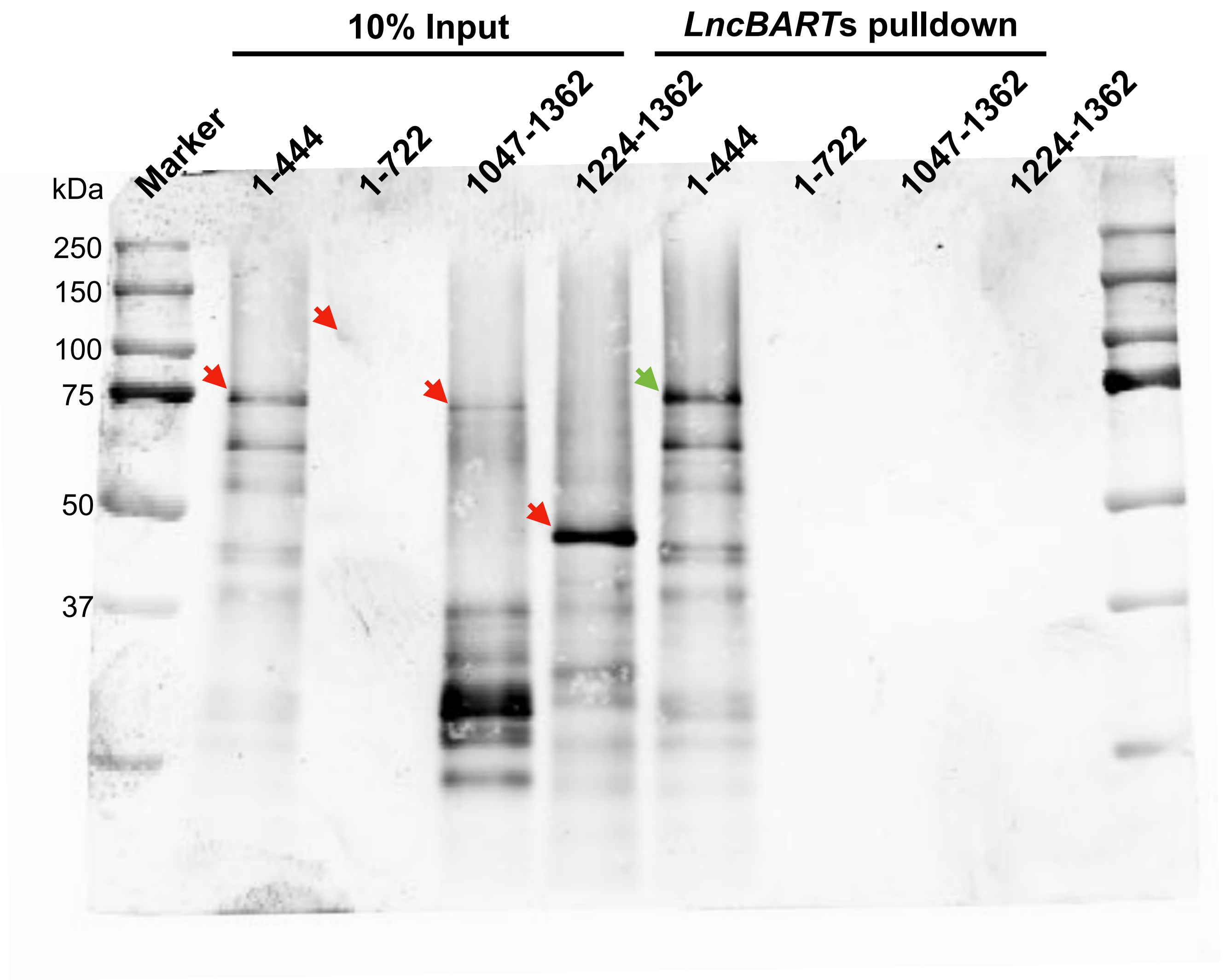

Fig.S3

a

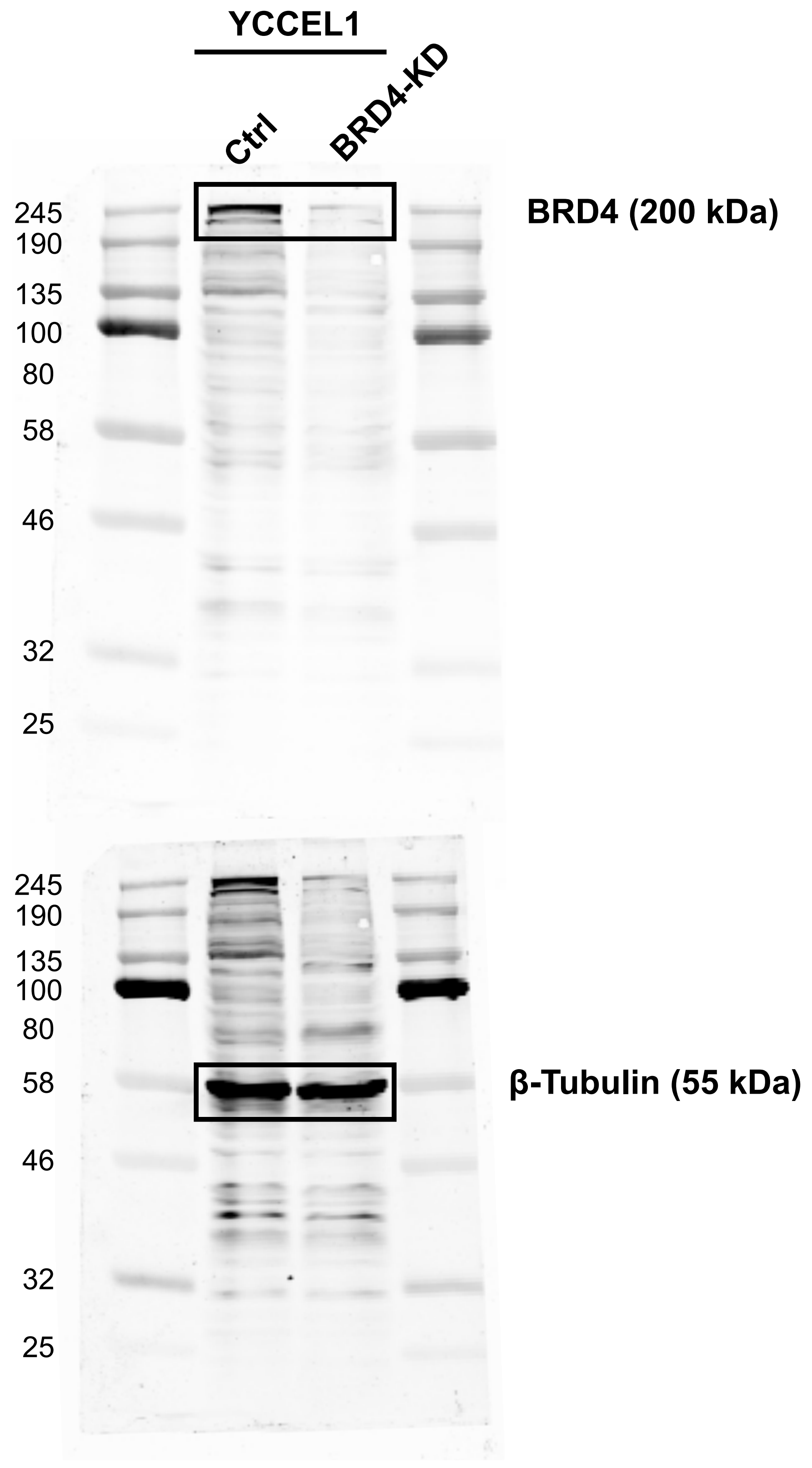

Fig.5

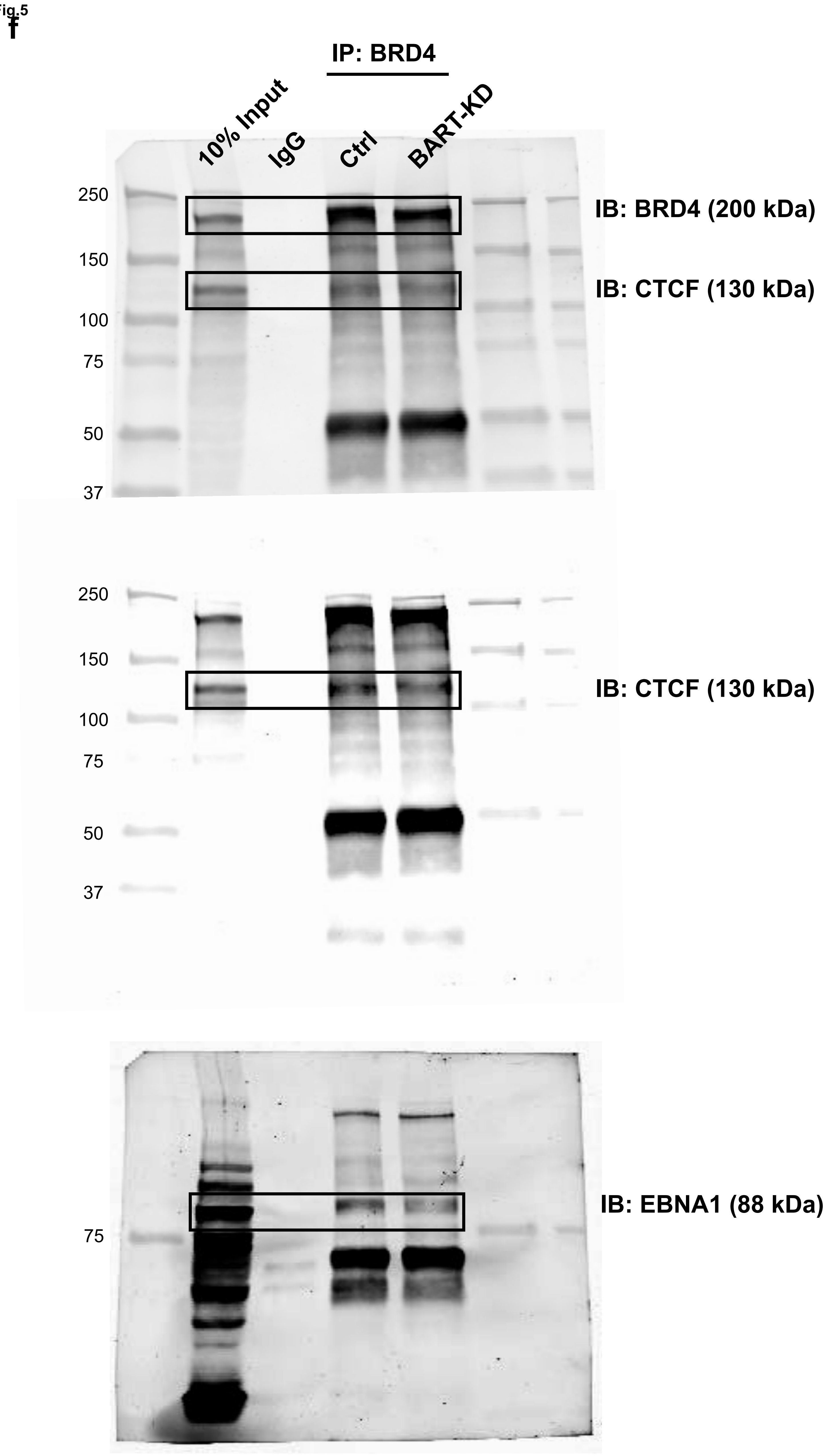

f
IP: BRD4
10% Input
IgG
Ctrl
BART-KD
250
150
100
75
50
37
IB: BRD4 (200 kDa)
IB: CTCF (130 kDa)
250
150
100
75
50
37
IB: CTCF (130 kDa)
75
IB: EBNA1 (88 kDa)

Fig.6
e

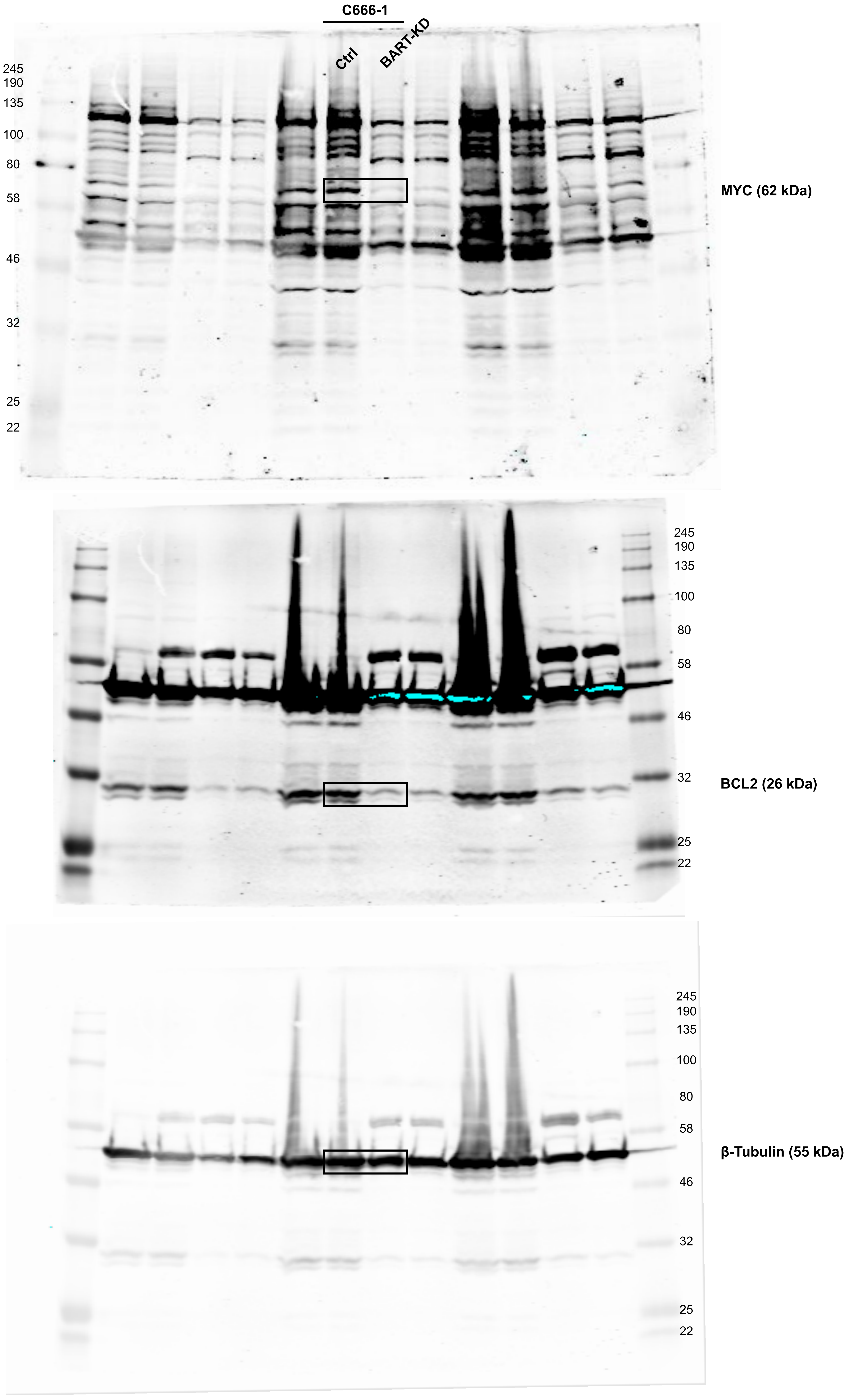
C666-1
Ctrl
BART-KD
245
190
135
100
80
58
46
32
25
22
MYC (62 kDa)
BCL2 (26 kDa)
β-Tubulin (55 kDa)

Fig.6

e

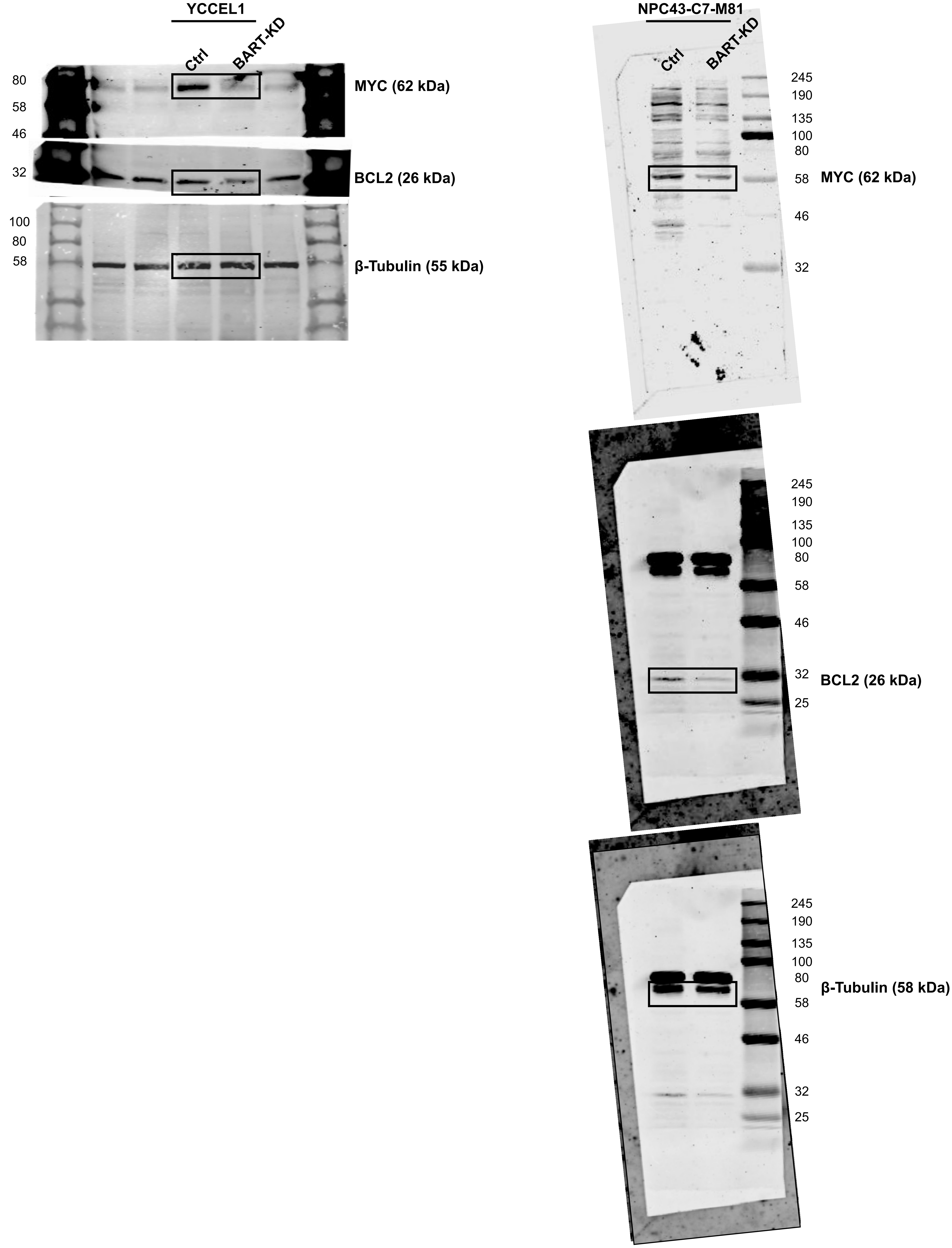

YCCEL1
Ctrl
BART-KD
80
58
46
MYC (62 kDa)
32
BCL2 (26 kDa)
100
80
58
β-Tubulin (55 kDa)
NPC43-C7-M81
Ctrl
BART-KD
245
190
135
100
80
58
MYC (62 kDa)
46
32
245
190
135
100
80
58
46
32
BCL2 (26 kDa)
25
245
190
135
100
80
β-Tubulin (58 kDa)
58
46
32
25

Fig.S2

j

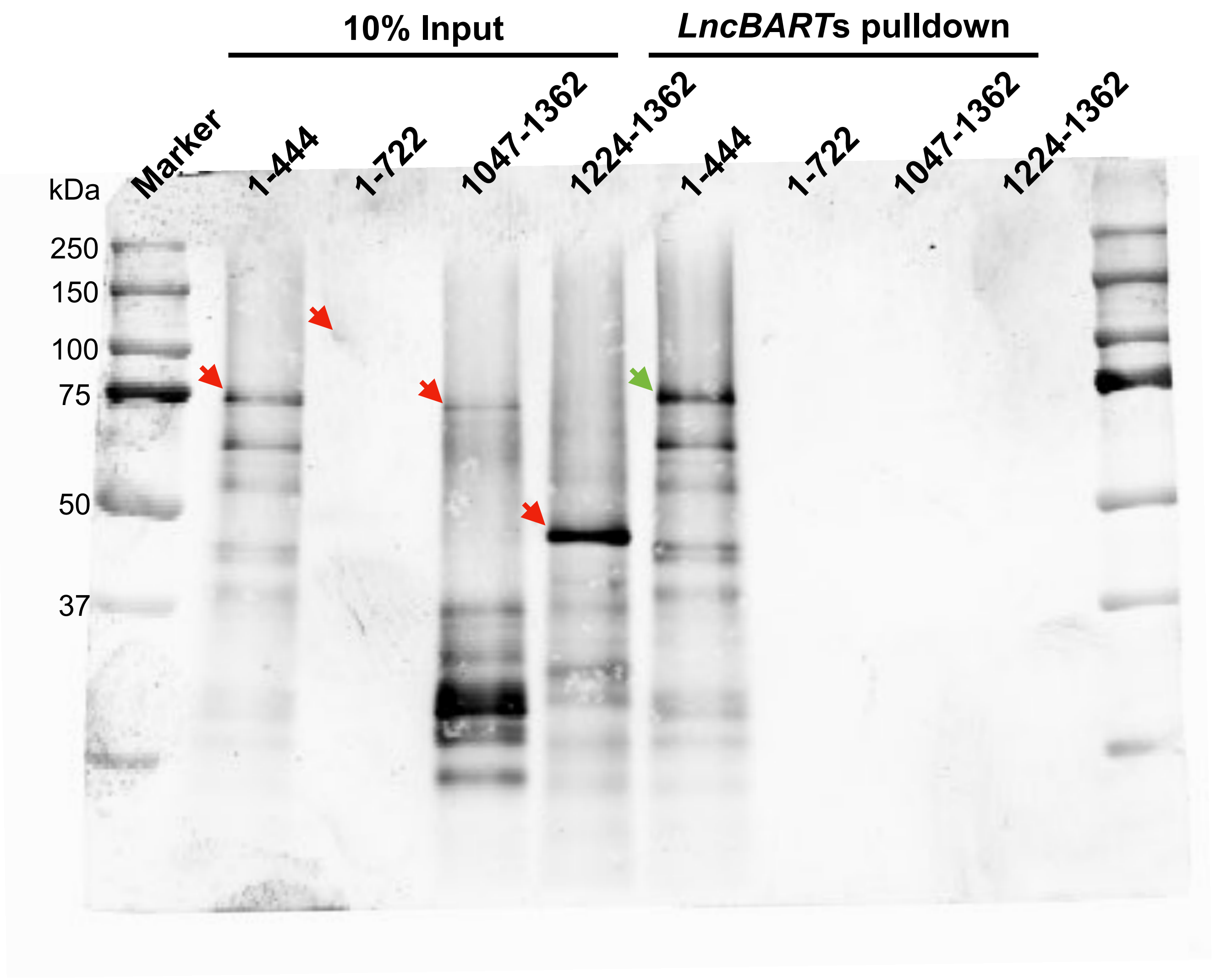

10% Input
LncBARTs pulldown
Marker
1-444
1-722
1047-1362
1224-1362
1-444
1-722
1047-1362
1224-1362
kDa
250
150
100
75
50
37
