# Supplementary material for: Epstein‐Barr Virus Expressed Long Non‐Coding RNA (lncBARTs) Regulate EBV Latent Genome Replication
Source: Adv Sci (Weinh). 2025 Nov 11;13(8):e07286. doi: 10.1002/advs.202507286 (PMC12884799; doi:10.1002/advs.202507286)
Supplement: Supplementary file 2 — Supporting Information [file ADVS-13-e07286-s002.pdf]

Fig.S1

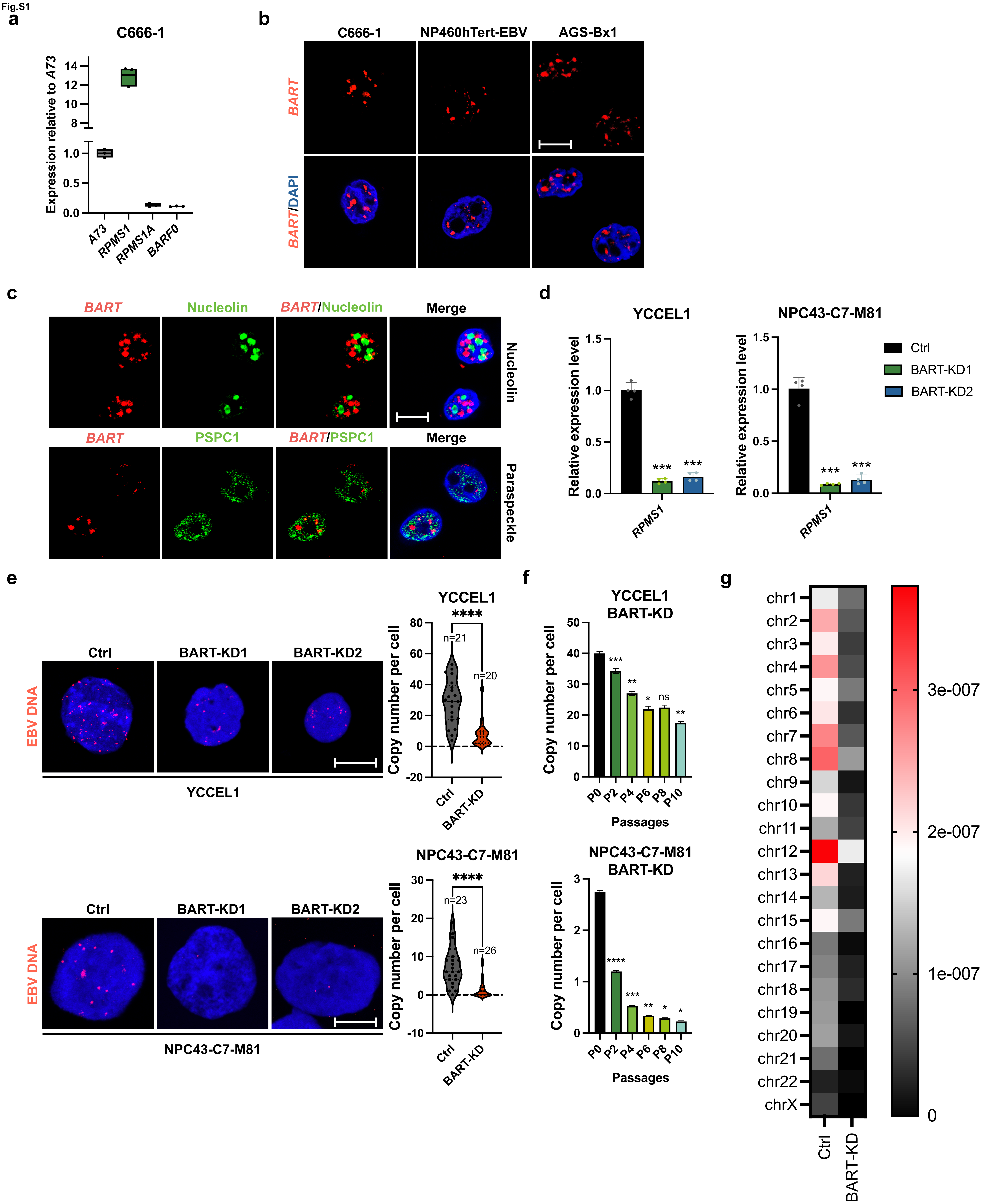

a
C666-1
Expression relative to A73
A73
RPMS1
RPMS1A
BARF0
b
C666-1
NP460hTert-EBV
AGS-Bx1
BART
BART/DAPI
c
BART
Nucleolin
BART/Nucleolin
Merge
Nucleolin
PSPC1
BART/PSPC1
Paraspeckle
d
YCCEL1
NPC43-C7-M81
Relative expression level
RPMS1
Ctrl
BART-KD1
BART-KD2
e
Ctrl
BART-KD1
BART-KD2
EBV DNA
YCCEL1
NPC43-C7-M81
Copy number per cell
n=21
n=20
n=23
n=26
****
BART-KD
f
YCCEL1
BART-KD
NPC43-C7-M81
BART-KD
Copy number per cell
Passages
P0
P2
P4
P6
P8
P10
g
chr1
chr2
chr3
chr4
chr5
chr6
chr7
chr8
chr9
chr10
chr11
chr12
chr13
chr14
chr15
chr16
chr17
chr18
chr19
chr20
chr21
chr22
chrX
Ctrl
BART-KD
3e-007
2e-007
1e-007
0

Fig.S1

h

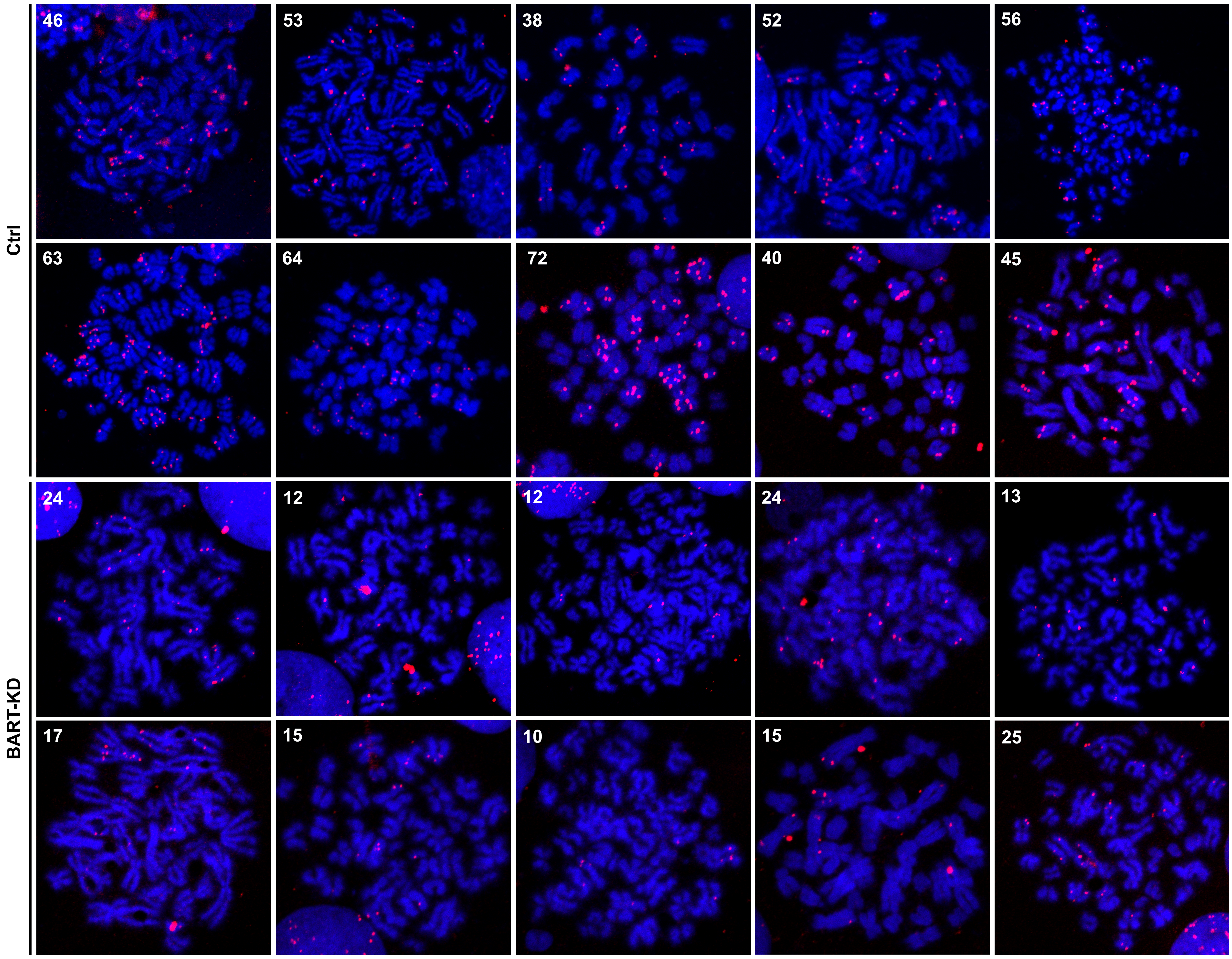
Ctrl
46
53
38
52
56
63
64
72
40
45
BART-KD
24
12
12
24
13
17
15
10
15
25

i

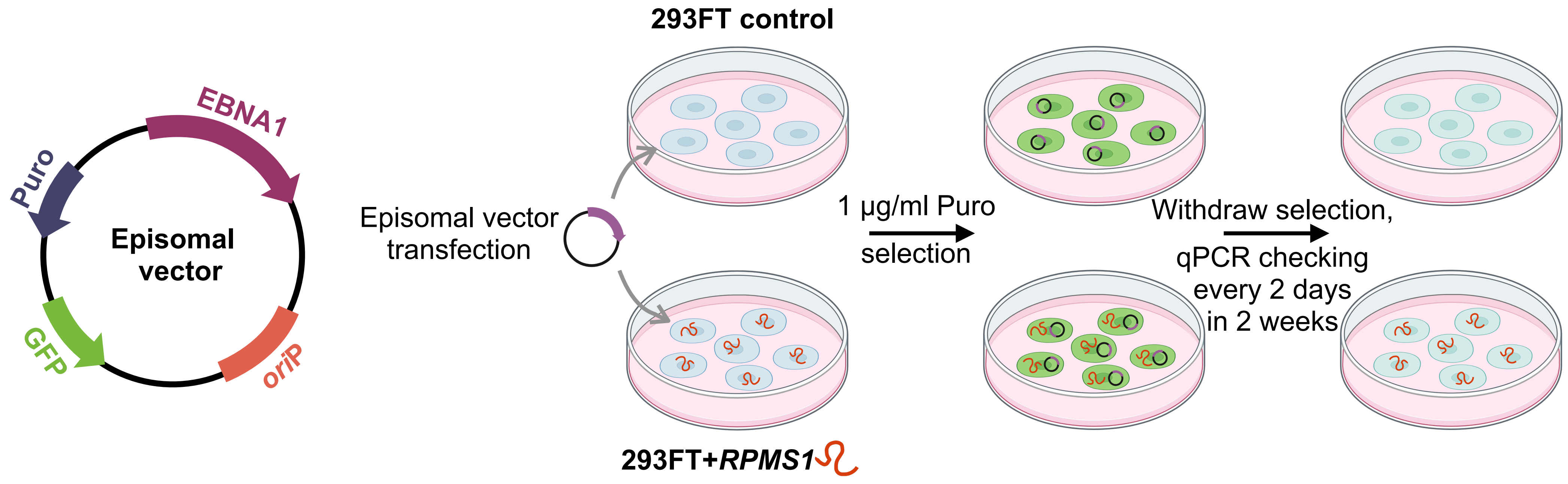
EBNA1
Puro
Episomal vector
GFP
oriP
Episomal vector transfection
293FT control
293FT+RPMS1
1 μg/ml Puro selection
Withdraw selection, qPCR checking every 2 days in 2 weeks

Fig.S1

Figure S1. Related to Figure 1 and 2.

(a) Expression of *RPMS1*, *A73*, *RPMS1A* and *BARF0* was analyzed by RT-qPCR in C666-1 cells. Gene expression was normalized to that of *GAPDH* and is presented as fold-difference compared to A73 (n = 3). (b) Representative images of RNA FISH assay for *lncBARTs* (red) in C666-1, NP460hTert-EBV and AGS-Bx1 cell lines. Scale bar, 10 μm. (c) Representative images of RNA FISH/IF assay for *lncBART*s (red) and subnuclear organelles (green) stained with markers for nucleolin and paraspeckles (PSPC1) in C666-1 cells. Scale bar, 10 μm. (d) *LncBART*s knockdown cells were generated by transfection with 4 pairs of dox inducible shRNAs targeting BART exons. Expression of *lncBART*s in YCCEL1 and NPC43-C7-M81 knockdown (passage 16) and control cells were analyzed by RT-qPCR (n = 3). Control represents scramble shRNA with no specific target on the genome. (e) Representative images of DNA FISH assay of EBV genome (red) levels in *lncBART*s knockdown (passage 16) YCCEL1 and NPC43-C7-M81 and control cells. Scale bar, 10 μm. Scale bar, 10 μm. The accompanying bar charts illustrate the quantification and statistical comparison of EBV copy number between *lncBART*s knockdown (n = 20) and control (n = 21) YCCEL1 cells, as well as between *lncBART*s knockdown (n = 28) and control (n = 23) NPC43-C7-M81 cells. (f) Average EBV copy number per cell *lncBART*s knockdown YCCEL1 and NPC43-C7-M81 and control cells after serial passage (n = 3), determined by qPCR. (g) A Hi-C heat map analysis comparison of EBV tethering sites on each chromosome in *lncBART*s knockdown and control C666-1 cells. (h) Representative images of DNA FISH assay showing the localization of EBV DNA copies (red) in ten control C666-1 cells and ten *lncBART*s knockdown C666-1 cells in mitotic spreads. The cells were treated with 0.5 mg/ml nocodazole (NOC) at 37°C for 16 hours to induce metaphase arrest. Scale bar, 10 μm. The number of EBV copies in each cell is displayed in white in the upper left corner of each image. (i) Schematic of the episomal vector maintenance test. This episomal vector consists of *ori*P/EBNA1 elements, a puromycin resistance gene (puro), and GFP. The episomal vector can replicate within eukaryotic cells and can be passed on to the daughter cells. Without drug selection, the episomal vector can be lost. The episomal vector was introduced into 293FT cells with or without *RPMS1*. The rates of episomal vector loss were measured every two days by qPCR targeting GFP sequence. Statistical analysis was performed using unpaired two-tailed Student's t-test. Data are presented as mean ± SEM. *p < 0.05, **p < 0.01, ***p < 0.001, ****p < 0.0001, ns, no significance.

Fig.S2

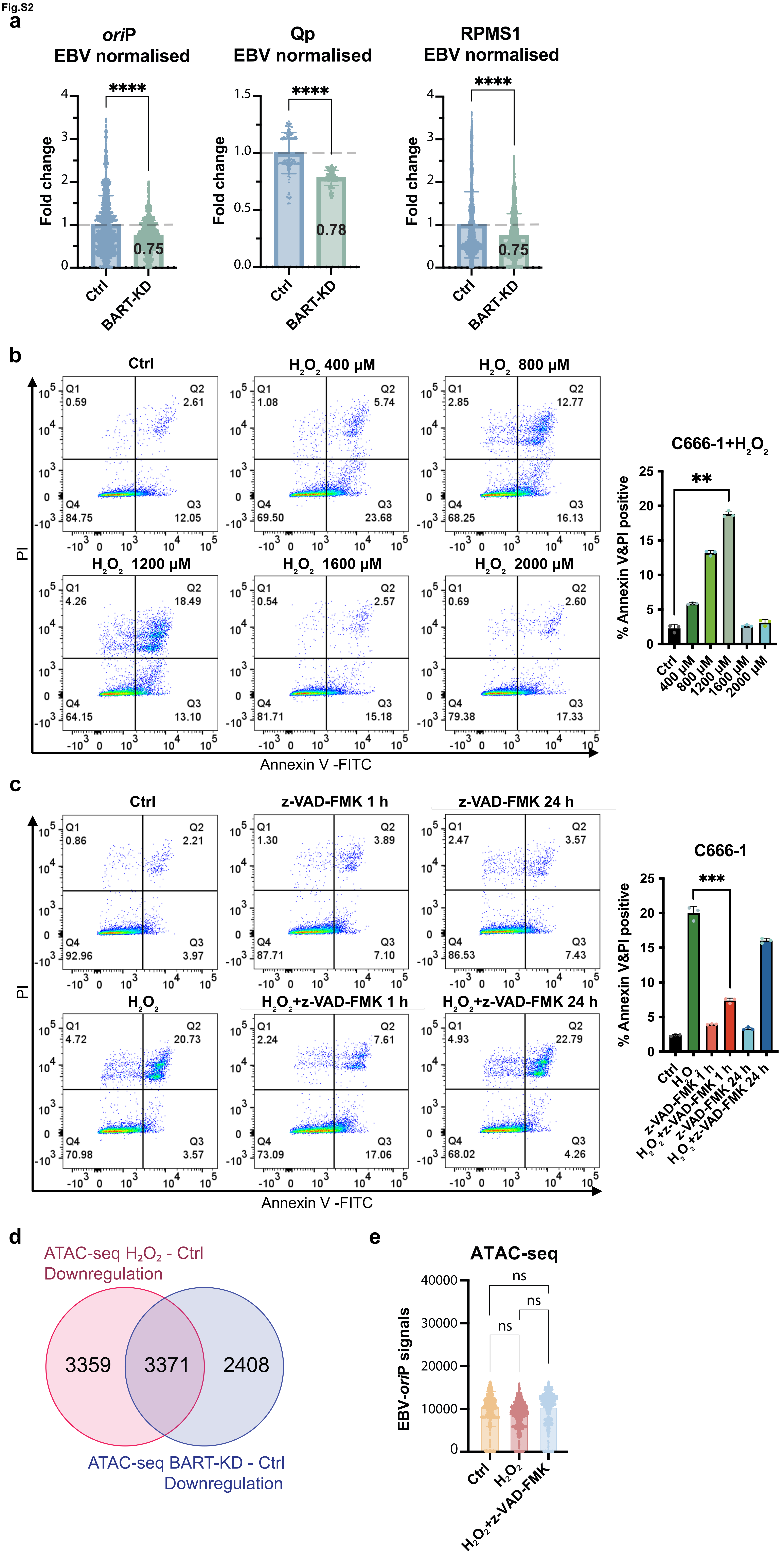
a
oriP
EBV normalised
Qp
EBV normalised
RPMS1
EBV normalised
Fold change
****
0.75
0.78
0.75
Ctrl
BART-KD
b
Ctrl
H2O2 400 μM
H2O2 800 μM
H2O2 1200 μM
H2O2 1600 μM
H2O2 2000 μM
Q1 0.59 Q2 2.61 Q4 84.75 Q3 12.05
Q1 1.08 Q2 5.74 Q4 69.50 Q3 23.68
Q1 2.85 Q2 12.77 Q4 68.25 Q3 16.13
Q1 4.26 Q2 18.49 Q4 64.15 Q3 13.10
Q1 0.54 Q2 2.57 Q4 81.71 Q3 15.18
Q1 0.69 Q2 2.60 Q4 79.38 Q3 17.33
PI
Annexin V -FITC
C666-1+H2O2
% Annexin V&PI positive
**
Ctrl
400 μM
800 μM
1200 μM
1600 μM
2000 μM
c
Ctrl
z-VAD-FMK 1 h
z-VAD-FMK 24 h
H2O2
H2O2+z-VAD-FMK 1 h
H2O2+z-VAD-FMK 24 h
Q1 0.86 Q2 2.21 Q4 92.96 Q3 3.97
Q1 1.30 Q2 3.89 Q4 87.71 Q3 7.10
Q1 2.47 Q2 3.57 Q4 86.53 Q3 7.43
Q1 4.72 Q2 20.73 Q4 70.98 Q3 3.57
Q1 2.24 Q2 7.61 Q4 73.09 Q3 17.06
Q1 4.93 Q2 22.79 Q4 68.02 Q3 4.26
PI
Annexin V -FITC
C666-1
% Annexin V&PI positive
***
d
ATAC-seq H2O2 - Ctrl
Downregulation
3359
3371
2408
ATAC-seq BART-KD - Ctrl
Downregulation
e
ATAC-seq
EBV-oriP signals
ns
ns
ns
Ctrl
H2O2
H2O2+z-VAD-FMK

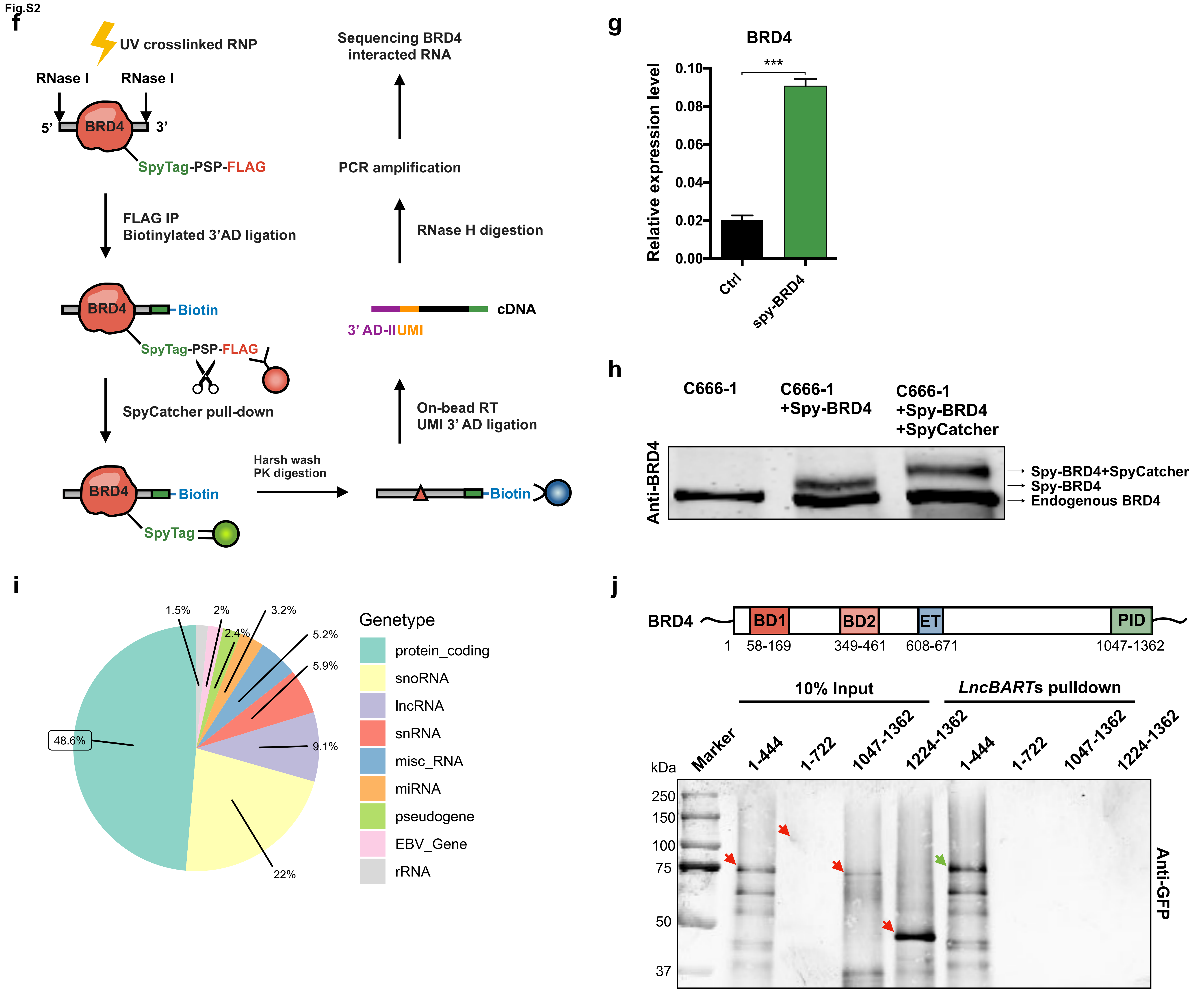

Figure S2. Related to Figure 3.

(a) Bar graph illustrating the quantitative ATAC-seq signals at the *ori*P, Qp and RPMS1 region, normalized to the EBV copy number for each condition. Fold change between control and *lncBART*s knockdown is indicated by the number on each column. (b) Flow cytometric analysis of Annexin V and PI staining of C666-1 cells treated with various concentrations of H_2O_2 (400 µM, 800 µM, 1200 µM, 1600 µM, and 2000 µM) for 24 h. Experiments were performed in triplicate, and representative flow cytometry plots are shown. The adjacent bar chart quantifies the percentage of Annexin V and PI-positive cells. (c) Flow cytometric analysis of Annexin V and PI staining of C666-1 cells pre-treated with 100 µM z-VAD-fmk for either 1 h or 24 h prior to exposure to 1200 µM H_2O_2 for 24 h. Experiments were performed in triplicate, and representative flow cytometry plots are shown. The adjacent bar chart quantifies the percentage of Annexin V and PI positive cells. (d) Venn diagram illustrating the overlap of downregulated genes identified by ATAC-seq in H_2O_2-treated (1200 µM, 24 h) versus control samples, and in *lncBART*s knockdown versus control samples. (e) Bar graph illustrating the quantitative ATAC-seq signals at the *ori*P region across different conditions: H_2O_2 treatment (1200 µM, 24 h), pre-treatment with z-VAD-fmk (100 µM, 1 h) followed by H_2O_2 exposure, and untreated controls. (f) Schematic representation of the SpyTag-based CLIP (SpyCLIP) procedure using the SpyTag-SpyCatcher system to study endogenous RNA binding proteins (RBPs). (g) RT-qPCR analysis of C666-1 cells overexpressing SpyTag fused BRD4. Gene expression was normalized to that of *GAPDH* (n = 3). (h) Western blot analysis of comparative expression levels of SpyTag fused BRD4 and its endogenous counterpart in C666-1 cells. (i) Peak annotation displaying the types of SpyCLIP identified BRD4-bound RNAs. (j) Schematic of BRD4 domain features. A RNA pulldown assay was conducted by utilizing biotinylated full-length *lncBART*s synthesized by *in vitro* transcription (IVT) to capture purified recombinant truncated BRD4 protein with a GFP tag expressed in E.coli, including BRD4 truncations which included the BD domains (1-444), both the BD and ET domains (1-722), and the PID domain (1047-1362 and 1224-1362). Red arrows indicate the target proteins in the input samples, and green arrows indicate the target proteins in the pulldown samples. Statistical analysis was performed using two-tailed Student's t-test. Data are presented as mean ± SEM. $^{**}p < 0.01$, $^{***}p < 0.001$, $^{****}p < 0.0001$, ns, no significance.

Fig.S3

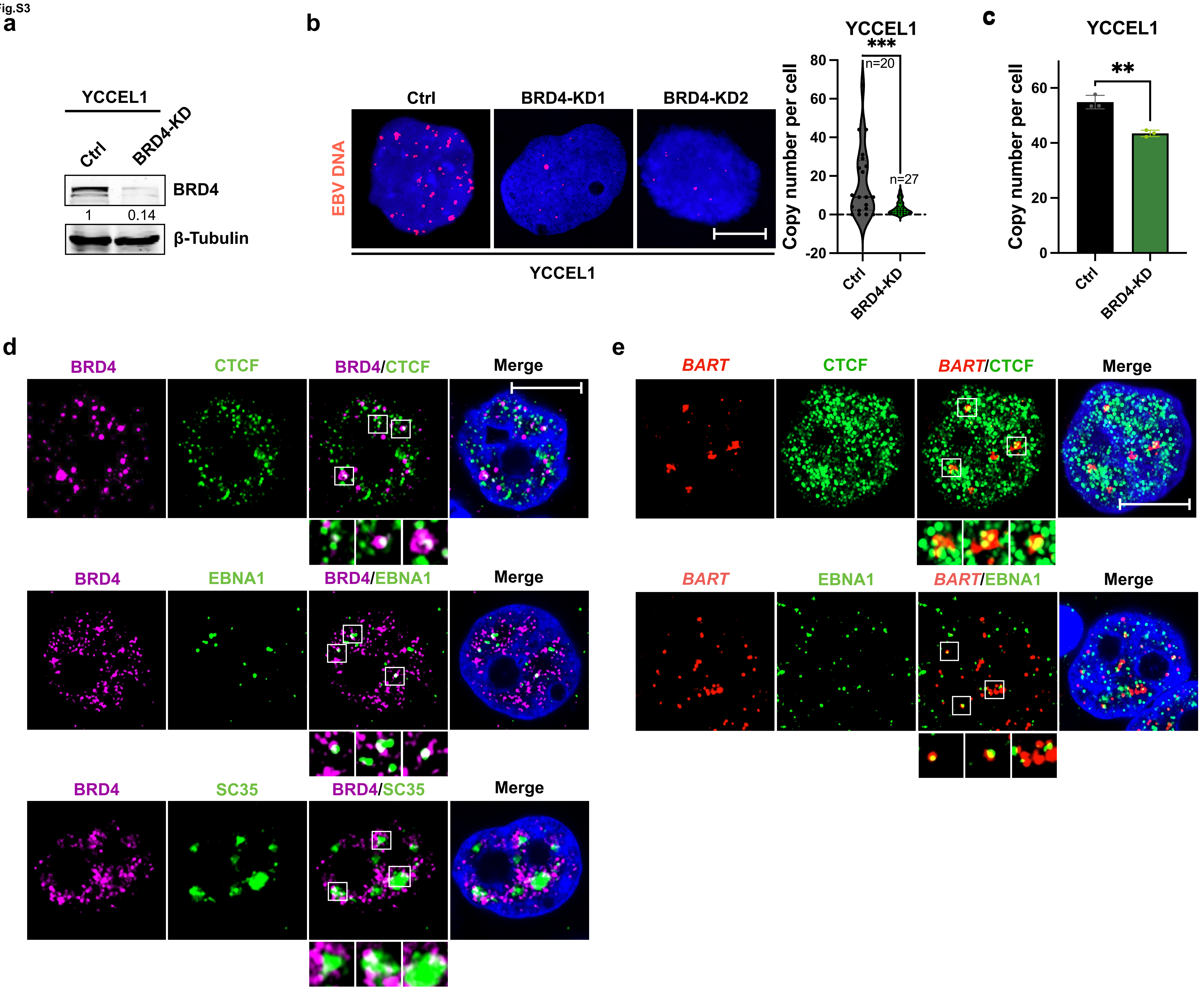
a
YCCEL1
Ctrl
BRD4-KD
BRD4
1
0.14
β-Tubulin
b
Ctrl
BRD4-KD1
BRD4-KD2
EBV DNA
YCCEL1
YCCEL1
***
n=20
n=27
Copy number per cell
80
60
40
20
0
-20
Ctrl
BRD4-KD
c
YCCEL1
**
Copy number per cell
60
40
20
0
Ctrl
BRD4-KD
d
BRD4
CTCF
BRD4/CTCF
Merge
BRD4
EBNA1
BRD4/EBNA1
Merge
BRD4
SC35
BRD4/SC35
Merge
e
BART
CTCF
BART/CTCF
Merge
BART
EBNA1
BART/EBNA1
Merge
