# Supplementary material for: Epstein‐Barr Virus Expressed Long Non‐Coding RNA (lncBARTs) Regulate EBV Latent Genome Replication
Source: Adv Sci (Weinh). 2025 Nov 11;13(8):e07286. doi: 10.1002/advs.202507286 (PMC12884799; doi:10.1002/advs.202507286)
Supplement: Supplementary file 3 — Supporting Information [file ADVS-13-e07286-s007.pdf]

Fig.S3

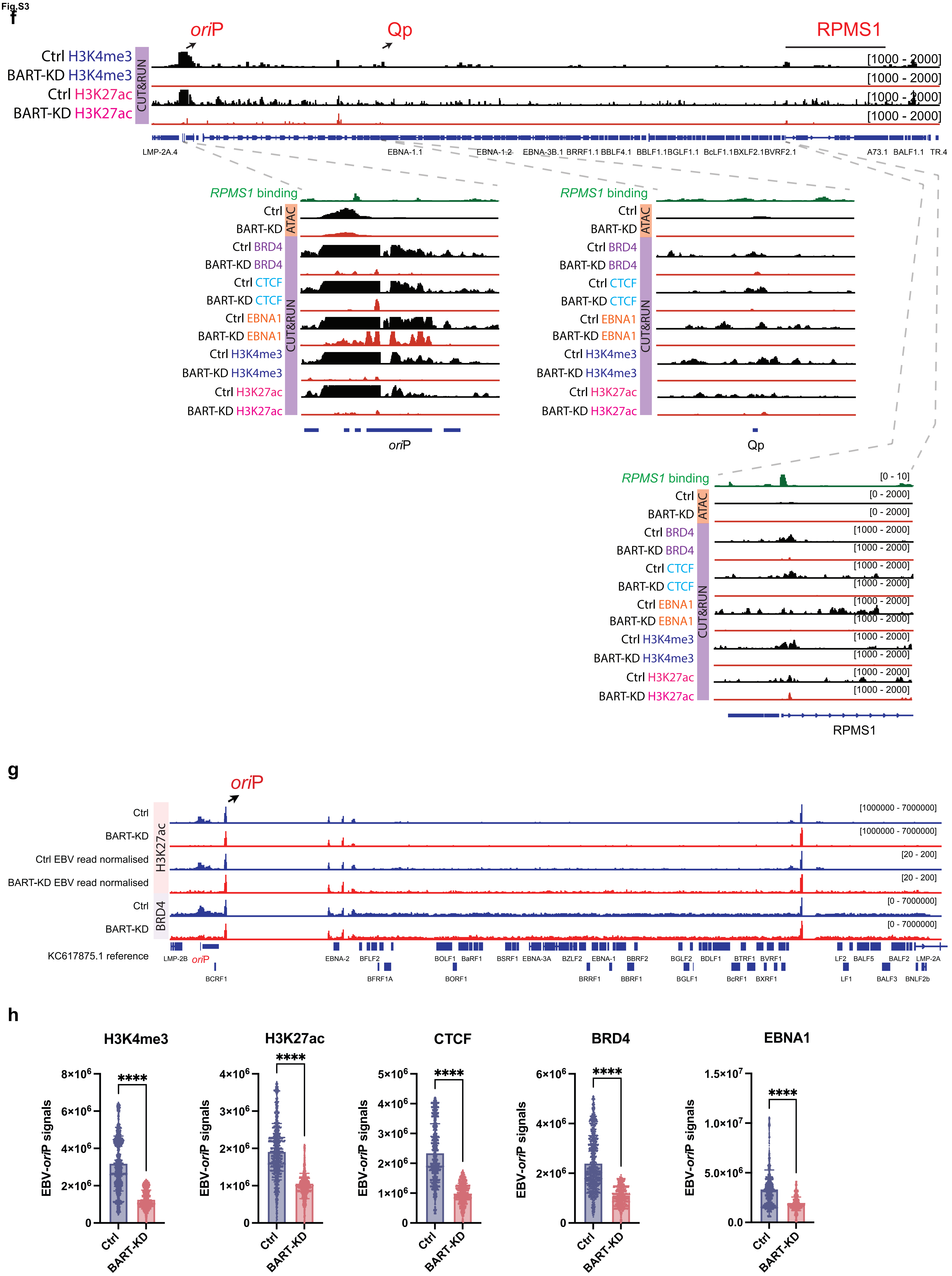
f
oriP
Qp
RPMS1
Ctrl H3K4me3
BART-KD H3K4me3
Ctrl H3K27ac
BART-KD H3K27ac
CUT&RUN
[1000 - 2000]
LMP-2A.4
EBNA-1.1
EBNA-1.2
EBNA-3B.1 BRRF1.1 BBLF4.1 BBLF1.1BGLF1.1 BcLF1.1BXLF2.1BVRF2.1
A73.1 BALF1.1 TR.4
RPMS1 binding
Ctrl
BART-KD
ATAC
Ctrl BRD4
BART-KD BRD4
Ctrl CTCF
BART-KD CTCF
Ctrl EBNA1
BART-KD EBNA1
Ctrl H3K4me3
BART-KD H3K4me3
Ctrl H3K27ac
BART-KD H3K27ac
oriP
Qp
[0 - 10]
[0 - 2000]
RPMS1
g
oriP
Ctrl
BART-KD
Ctrl EBV read normalised
BART-KD EBV read normalised
H3K27ac
BRD4
[1000000 - 7000000]
[20 - 200]
[0 - 7000000]
KC617875.1 reference
LMP-2B oriP BCRF1 EBNA-2 BFLF2 BFRF1A BOLF1 BaRF1 BORF1 BSRF1 EBNA-3A BZLF2 EBNA-1 BRRF1 BBRF2 BBRF1 BGLF2 BGLF1 BDLF1 BTRF1 BVRF1 BcRF1 BXRF1 LF2 LF1 BALF5 BALF3 BALF2 BNLF2b LMP-2A
h
H3K4me3
H3K27ac
CTCF
BRD4
EBNA1
EBV-oriP signals
****
Ctrl
BART-KD
8×10^6
6×10^6
4×10^6
2×10^6
0
3×10^6
1×10^6
5×10^6
1.5×10^7
1.0×10^7
5.0×10^6
0.0

Fig.S3

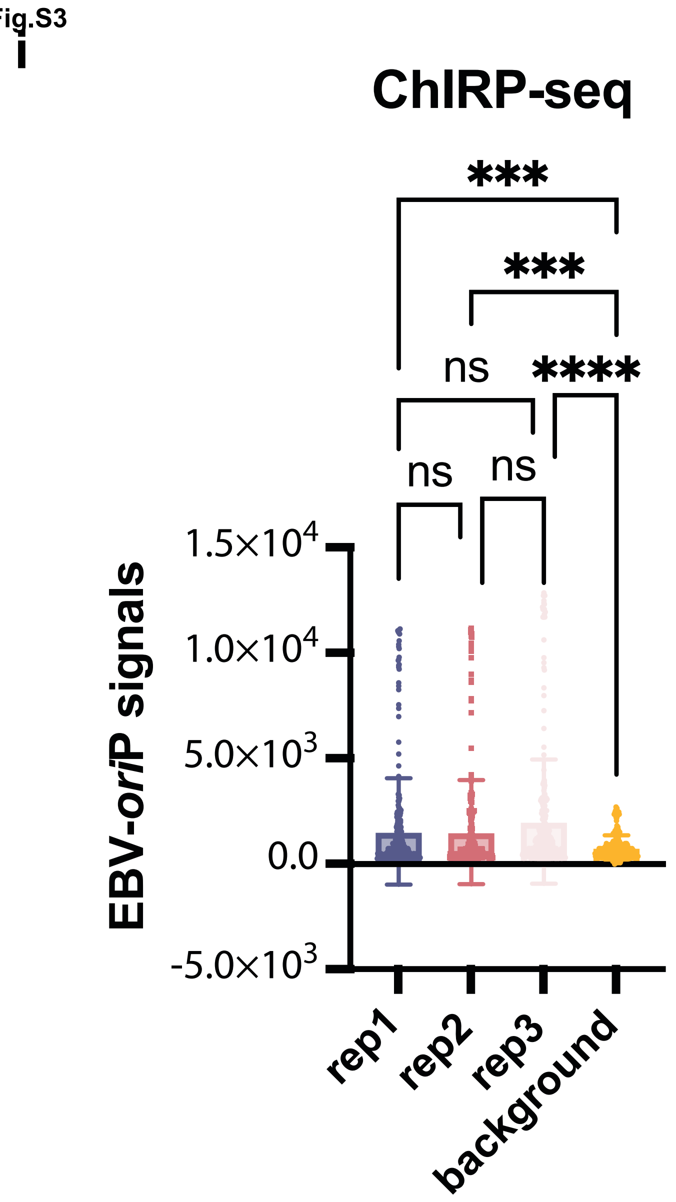

Figure S3. Related to Figures 5.

(a) Expression levels of BRD4 were evaluated by western blot in BRD4 knockdown and control YCCEL1 cells. (b) Representative images of DNA FISH assay of EBV genome (red) in BRD4 knockdown and control YCCEL1 cells. Scale bar, 10 µm. The accompanying bar charts illustrate the quantification and statistical comparison of EBV copy number between BRD4 knockdown (n = 20) and control (n = 27) cells. (c) EBV copy number as determined by qPCR in BRD4 knockdown YCCEL1 cells and controls (n = 3). (d) Representative images of IF assay for BRD4 (violet) and either CTCF, EBNA1 or SC35 (green) in C666-1 cells. Cropped and enlarged areas indicate the proteins complexes. Scale bar, 10 µm. (e) Representative images of RNA FISH/IF assay for *lncBARTs* (red), CTCF and EBNA1 (green) in C666-1 cells. Cropped and enlarged areas indicate *lncBARTs*-protein complexes. Scale bar, 10 µm. (f) The CUT&RUN-seq track images display the binding profiles of H3K4me3 and H3K27ac on the EBV genome (NC_007605.1) in *lncBARTs* knockdown and control C666-1 cells, visualized using IGV. Areas of co-occupancy adjacent to *ori*P, Qp and *RPMS1* revealed in ChIRP-seq, ATAC-seq, BRD4, CTCF, EBNA1, H3K4me3 and H3K27ac CUT&RUN-seq are highlighted and illustrated in detail in the lower panel. (g) The CUT&RUN-seq track images display the binding profiles of H3K27ac and BRD4 on the EBV genome (KC617875.1) in *lncBARTs* knockdown and control C666-1 cells, visualized using IGV. (h) Quantitative analysis of CUT&RUN-seq signals for H3K4me3, H3K27ac, CTCF, BRD4 and EBNA1 at the *ori*P region of the EBV genome (GenBank accession number KC617875.1) in *lncBARTs* knockdown and control C666-1 cells. (i) Quantitative analysis of ChIRP-seq data for *lncBARTs* at the same *ori*P region of the EBV genome (KC617875.1) in *lncBARTs* knockdown and control C666-1 cells. Statistical analysis was performed using two-tailed Student's t-test. Data are presented as mean ± SEM. **p < 0.001, ***p < 0.001, ****p < 0.0001, ns, no significance.

Fig.S4

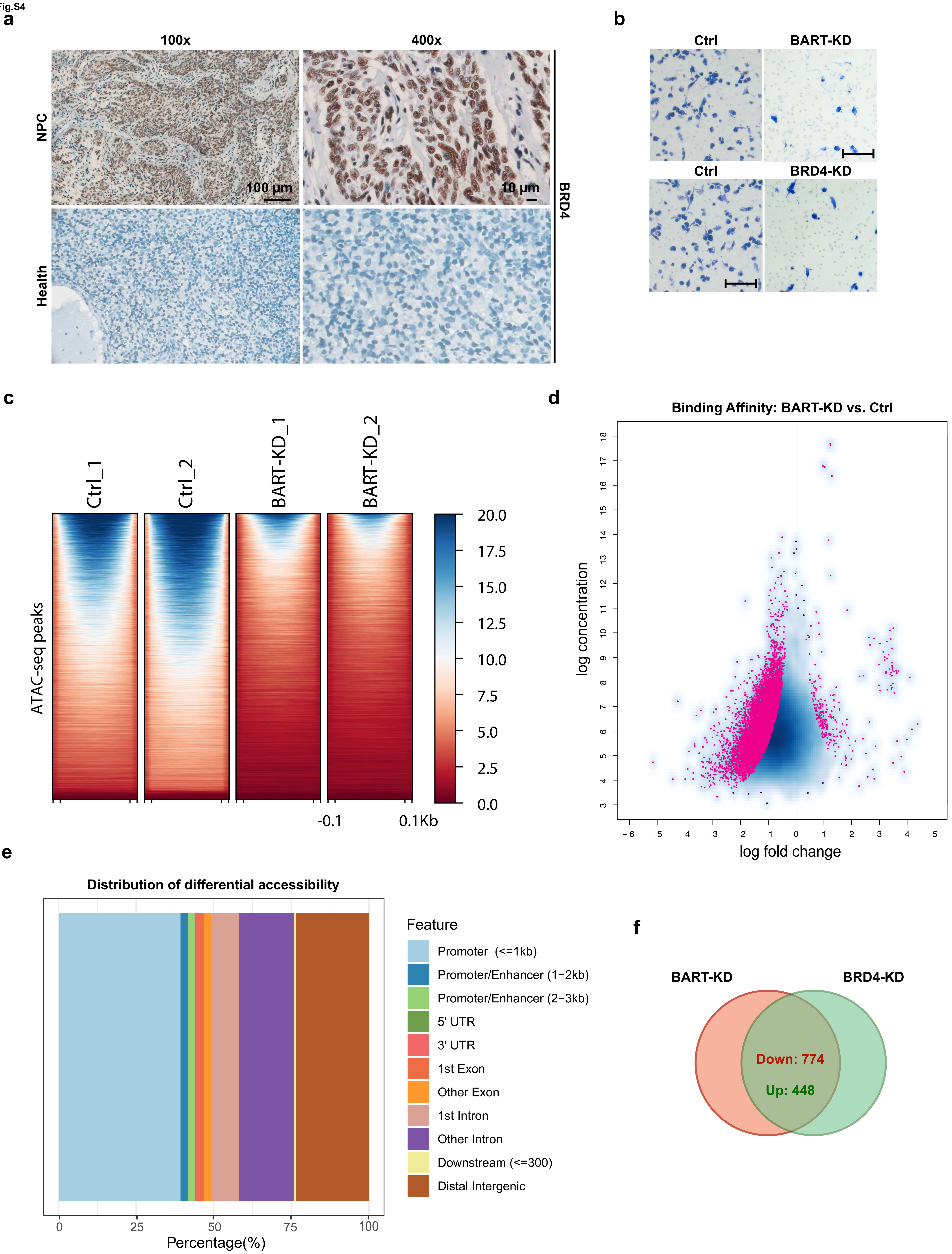

a
100x
400x
NPC
Health
100 μm
10 μm
BRD4
b
Ctrl
BART-KD
Ctrl
BRD4-KD
c
Ctrl_1
Ctrl_2
BART-KD_1
BART-KD_2
ATAC-seq peaks
20.0
17.5
15.0
12.5
10.0
7.5
5.0
2.5
0.0
-0.1
0.1Kb
d
Binding Affinity: BART-KD vs. Ctrl
log concentration
log fold change
e
Distribution of differential accessibility
Percentage(%)
0
25
50
75
100
Feature
Promoter (<=1kb)
Promoter/Enhancer (1−2kb)
Promoter/Enhancer (2−3kb)
5' UTR
3' UTR
1st Exon
Other Exon
1st Intron
Other Intron
Downstream (<=300)
Distal Intergenic
f
BART-KD
BRD4-KD
Down: 774
Up: 448

Fig.S4

g

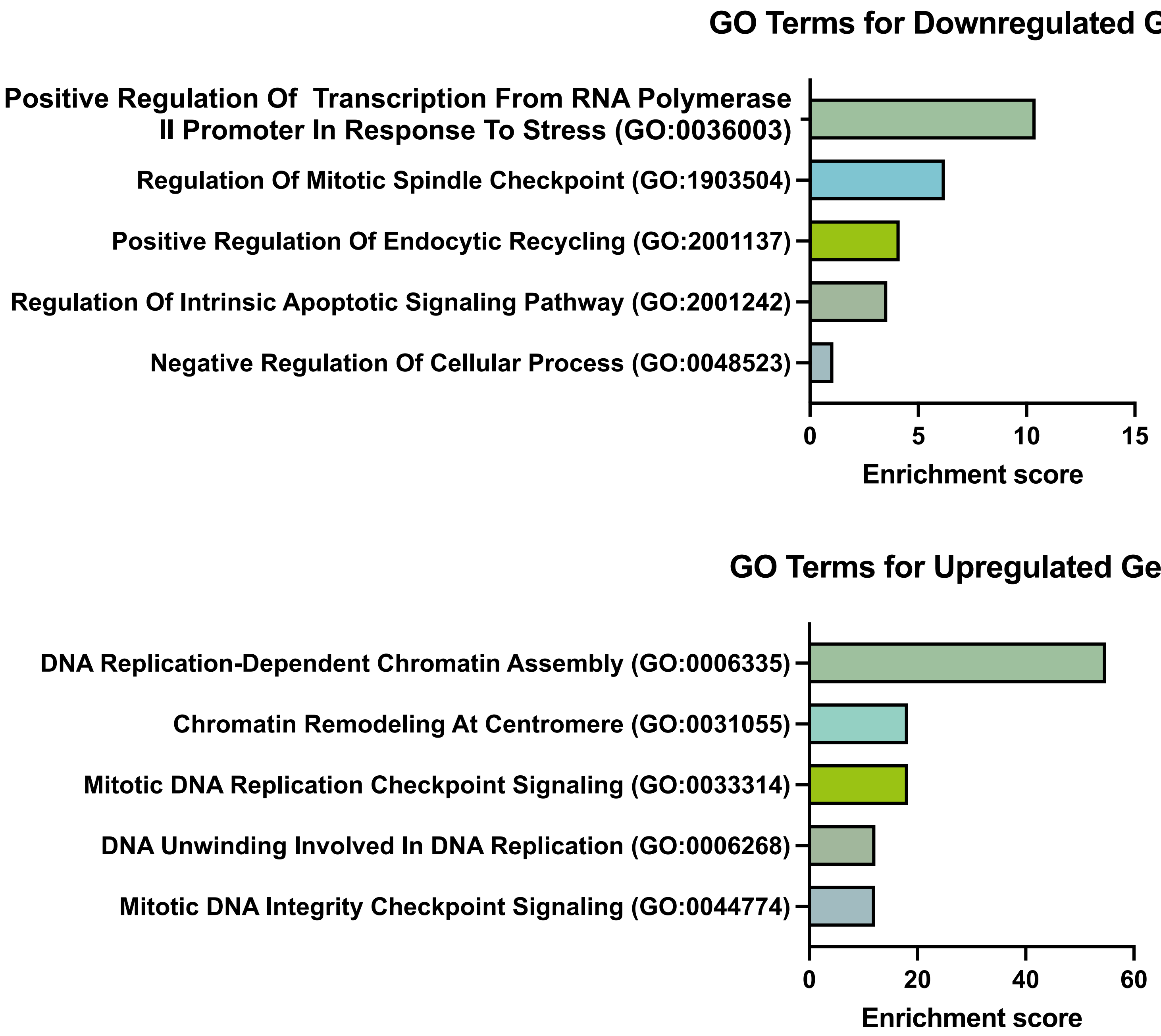
GO Terms for Downregulated Genes
Positive Regulation Of Transcription From RNA Polymerase II Promoter In Response To Stress (GO:0036003)
Regulation Of Mitotic Spindle Checkpoint (GO:1903504)
Positive Regulation Of Endocytic Recycling (GO:2001137)
Regulation Of Intrinsic Apoptotic Signaling Pathway (GO:2001242)
Negative Regulation Of Cellular Process (GO:0048523)
0 5 10 15
Enrichment score
GO Terms for Upregulated Genes
DNA Replication-Dependent Chromatin Assembly (GO:0006335)
Chromatin Remodeling At Centromere (GO:0031055)
Mitotic DNA Replication Checkpoint Signaling (GO:0033314)
DNA Unwinding Involved In DNA Replication (GO:0006268)
Mitotic DNA Integrity Checkpoint Signaling (GO:0044774)
0 20 40 60
Enrichment score

h

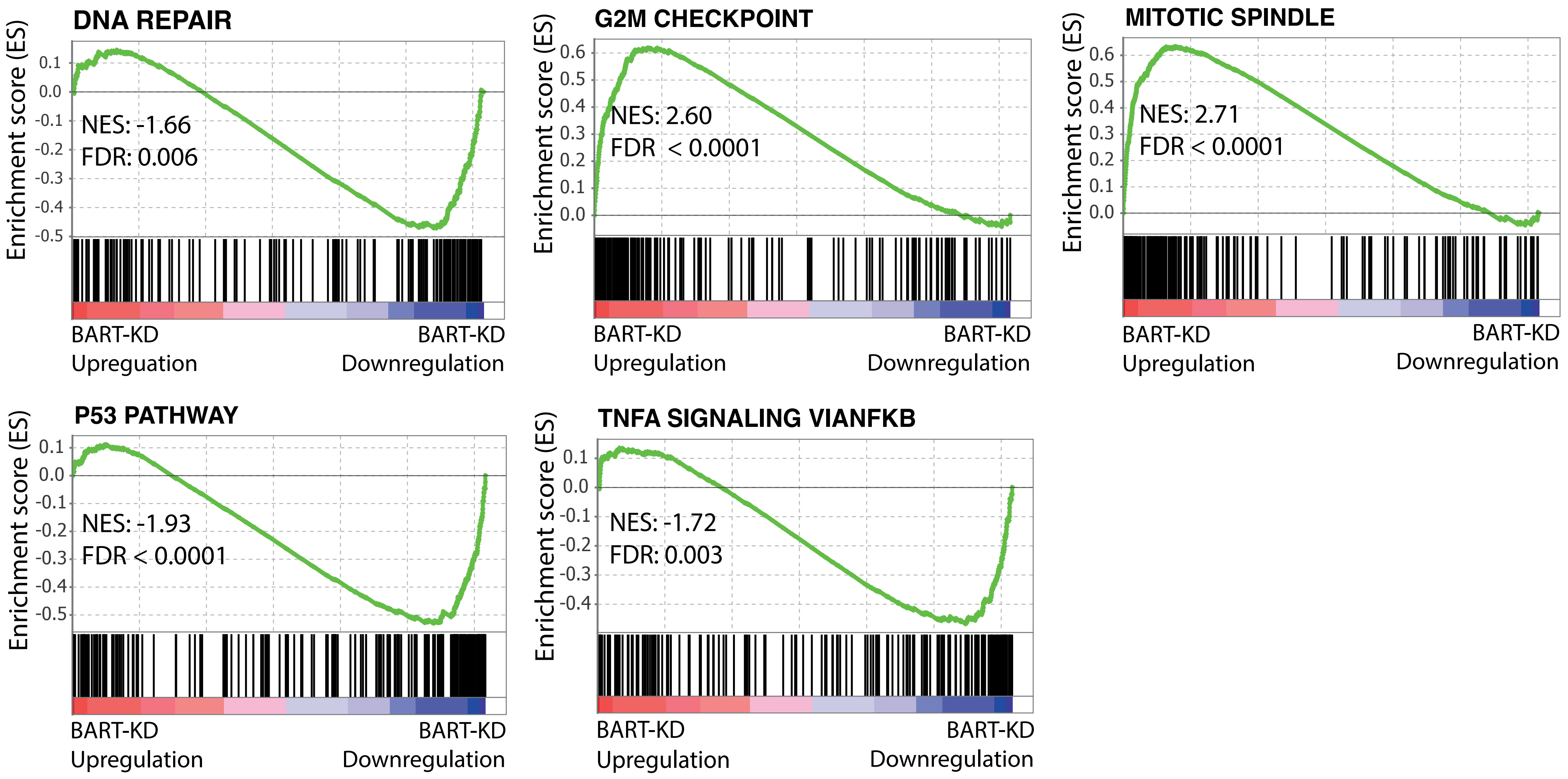
DNA REPAIR
Enrichment score (ES)
NES: -1.66
FDR: 0.006
BART-KD Upreguation
BART-KD Downregulation
G2M CHECKPOINT
NES: 2.60
FDR < 0.0001
BART-KD Upregulation
BART-KD Downregulation
MITOTIC SPINDLE
NES: 2.71
FDR < 0.0001
BART-KD Upregulation
BART-KD Downregulation
P53 PATHWAY
NES: -1.93
FDR < 0.0001
BART-KD Upregulation
BART-KD Downregulation
TNFA SIGNALING VIANFKB
NES: -1.72
FDR: 0.003
BART-KD Upregulation
BART-KD Downregulation

i

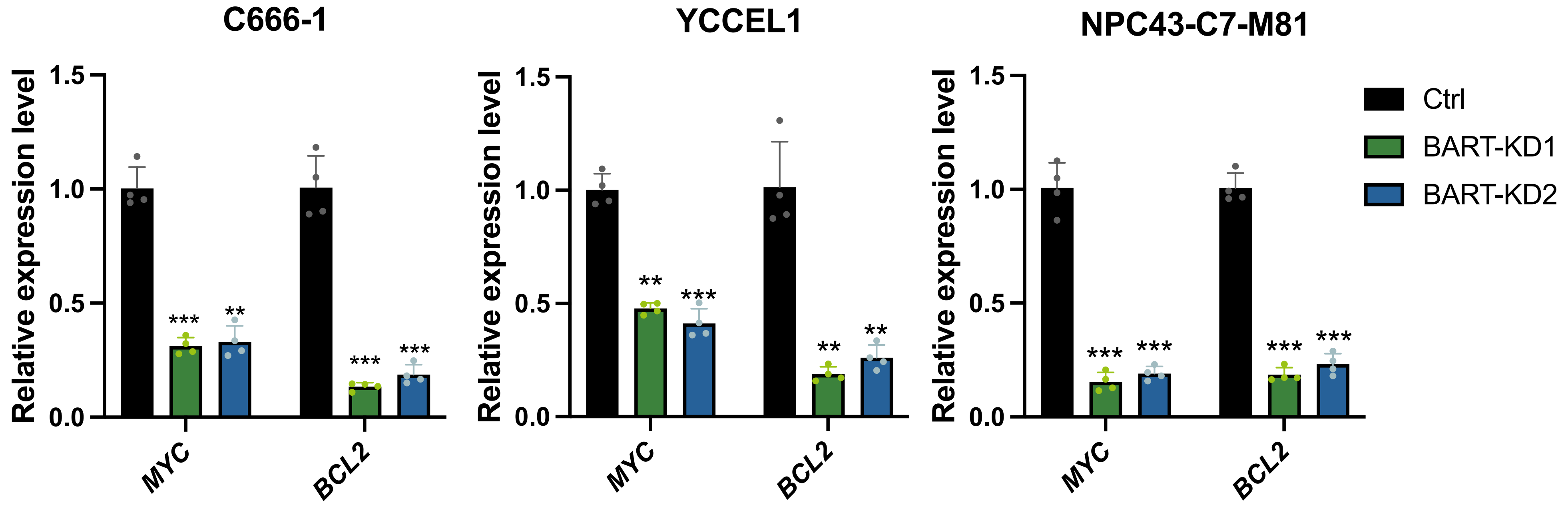
C666-1
YCCEL1
NPC43-C7-M81
Relative expression level
MYC
BCL2
Ctrl
BART-KD1
BART-KD2

j

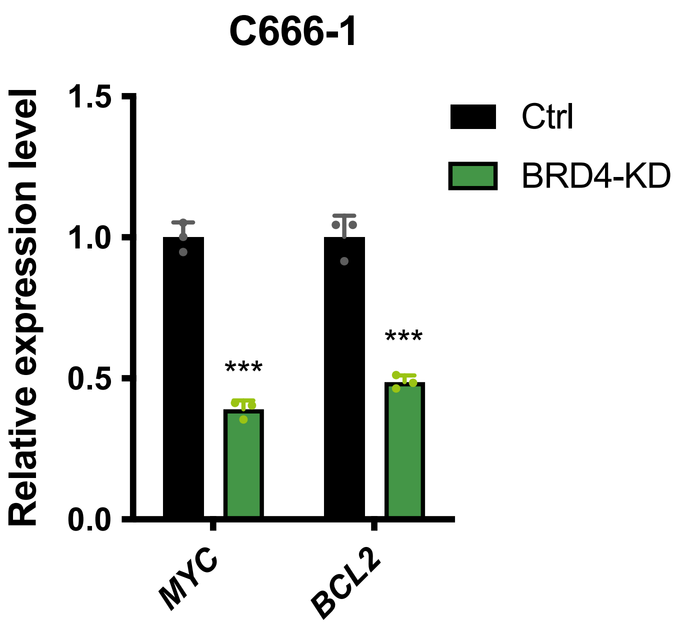
C666-1
Relative expression level
MYC
BCL2
Ctrl
BRD4-KD

Fig.S4

Figure S4. Related to Figure 6.

(a) Immunohistochemical staining at 100x (left) and 400x (right) magnification shows BRD4 expression staining in NPC biopsies of stage IV (top) and healthy control (bottom). Sections were stained with anti-BRD4 antibody. Scale bar, 100 μm (left), 10 μm (right). (b) Transwell invasion assay indicates the invasion abilities in *lncBARTs* and BRD4 knockdown (passage 20) and control YCCEL1 cells (passage 20). Scale bar, 100 μm. (c) A plotHeatmap analysis of the ATAC-seq signals in *lncBARTs* knockdown C666-1 and control cells. (d) The MA plot summarizes ATAC-seq coverage at the 7664 called peaks of differential accessibility that were identified. Peaks that passed a threshold of $padj < 0.05$ and absolute $log2foldChange > 1$ in differential accessibility analysis are colored in pink. The relative loss of open chromatin regions in *lncBARTs* knockdown C666-1 cells was below the 0-threshold line (blue vertical line), while the relative gain of open chromatin regions in *lnc-BARTs* knockdown C666-1 cells was above the 0-threshold line. (e) The peak annotation chart illustrates the distribution of differential accessibility across various genomic regions, including the promoter, enhancer, 5' UTR, 3'UTR, exon, intron, and intergenic region. (f) The Venn diagram illustrating overlap of down-regulated and up-regulated genes in RNA-seq of *lncBARTs* and BRD4 knockdown C666-1 cells, in comparison to the control cells. (g) GO pathway enrichment analysis for all differentially expressed genes that are either down-regulated and up-regulated in *lncBARTs* and BRD4 knockdown C666-1 cells, in comparison to the control cells. (h) GSEA enrichment plots show significant enrichment of Hallmark gene sets related to DNA repair, G2M checkpoint, mitosis spindle, p53 pathway and TNFA signalling via NFKB in *lncBARTs* knockdown C666-1 compared to control cells, as determined by RNA-seq. Black bars in the red region indicate genes within the corresponding gene set that are upregulated, while black bars in the blue region indicate genes that are downregulated. NES and FDR are provided for each gene set. NES, Normalized Enrichment Score. FDR, False Discovery Rate q-value. (i) The expression levels of *MYC* and *BCL2* in *lncBARTs* knockdown C666-1, YCCEL1, and NPC43-C7-M81 cells, along with their controls, were assessed using qPCR analysis, gene expression was normalized to that of *GAPDH* ($n = 3$). (j) The expression levels of *MYC* and *BCL2* in BRD4 knockdown and control C666-1 cells, were assessed using qPCR analysis, gene expression was normalized to that of *GAPDH* ($n = 3$). Statistical analysis was performed using two-tailed Student's t-test. Data are presented as mean ± SEM. $^{**}p < 0.01$, $^{***}p < 0.001$.
