# Supplementary material for: Epstein‐Barr Virus Expressed Long Non‐Coding RNA (lncBARTs) Regulate EBV Latent Genome Replication
Source: Adv Sci (Weinh). 2025 Nov 11;13(8):e07286. doi: 10.1002/advs.202507286 (PMC12884799; doi:10.1002/advs.202507286)
Supplement: Supplementary file 5 — Supporting Information [file ADVS-13-e07286-s001.pdf]

PANGENIA
新亞生命科技
A Pangenia Company

PANGENIA LIFESCIENCES LTD
10/F, Stelux House,
No.698 Prince Edward Road East,
San Po Kong, Kowloon, Hong Kong

T +852 3989 6160
F +852 3989 6161
E info@pangenialife.com
pangenialife.com

# Cell Line Authentication Test Report

| | | | |
|---|---|---|---|
| **Investigator Name:** | Jiayan LIU | **Laboratory No.:** | L-250711733P |
| **Institution/Company:** | Department of Microbiology, HKU | **Date of Registration:** | 17 July 2025 |
| **Sample Type:** | DNA | **Date of Issue:** | 29 July 2025 |
| **Sample Name:** | C666-1 P118 | **Report No.:** | 2500000421 |

## TEST RESULTS

| DNA Marker | C666-1 (CVCL_7949)[4] | C666-1 P118 (L-250711733P) |
|---|---|---|
| AMEL | X, Y | X, Y |
| CSF1PO | 11, 15, 16 | 11, 16 |
| D13S317 | 8, 11 | 8, 11 |
| D16S539 | 10 | 10 |
| D5S818 | 11, 12 | 11 |
| D7S820 | 11, 12 | 11, 12 |
| TH01 | 6, 8 | 6, 8 |
| TPOX | 8, 11 | 8, 11 |
| vWA | 17, 18 | 17, 18 |
| D18S51 | 16 | 15, 16 |
| D21S11 | 28, 29, 30.2, 31.2 | 29, 31.2 |
| D3S1358 | 16, 17 | 16, 17 |
| D8S1179 | 11, 13, 14, 15 | 11, 14, 15 |
| FGA | 23, 24 | 23, 24 |
| Penta D | 9, 10 | 9, 10 |
| Penta E | 11, 15 | 10, 15 |
| Number of shared alleles | | 30 |
| Total number of alleles in the reference profile | | 35 |
| Percent match | | 86% |

## COMMENT

The tested cell line has a 86% match with C666-1. Therefore, they are considered to be related from a common ancestry.

**Assay Description & Methodology:**

This test examines 15 human autosomal short tandem repeat (STR) loci and Amelogenin using the PowerPlex® 16HS Kit[1]. Sample DNA is subjected to PCR amplification and is then separated by capillary electrophoresis. The matching criterion is based on an algorithm recommended by the ATCC® Standards Development Organization[2] that compares the number of shared alleles between two cell line samples, expressed as a percentage (Percent match). Cell lines with ≥ 80% match are considered to be related and derived from a common ancestry[2,3]. Cell lines with 55% - 80% match require further analysis for authentication of relatedness. Cell lines with ≤ 55% match are considered unrelated. The test sample is reported as cross-contaminated when the STR profile involves more than the theoretical number of alleles at three or more loci[2].

***Signed out by:***

For and on behalf of Pangenia Lifesciences Ltd.

**References:**

1. PowerPlex® 16HS System Technical Manual, Part#TMD022, Promega Corporation.
2. ANSI/ATCC ASN-0002-2011, Designation: ASN-0002 "Authentication of Human Cell Lines: Standardization of STR Profiling"
3. JR Masters *et al.* Proc Natl Acad Sci USA 2001; 98:8012–7.
4. Cellosaurus 2019. CLASTR 1.4.4. https://web.expasy.org/cellosaurus-str-search.

PANGENIA
新亞生命科技
A Pangenia Company

PANGENIA LIFESCIENCES LTD
10/F, Stelux House,
No.698 Prince Edward Road East,
San Po Kong, Kowloon, Hong Kong
T +852 3989 6160
F +852 3989 6161
E info@pangenialife.com
pangenialife.com

# Cell Line Authentication Test Report

| | | | |
|---|---|---|---|
| **Investigator Name:** | Jiayan LIU | **Laboratory No.:** | L-250711740P |
| **Institution/Company:** | Department of Microbiology, HKU | **Date of Registration:** | 17 July 2025 |
| **Sample Type:** | DNA | **Date of Issue:** | 26 July 2025 |
| **Sample Name:** | NPC43 P164 | **Report No.:** | 2500000428 |

## TEST RESULTS

| DNA Marker | NPC43 (CVCL_UH64)[4] | NPC43 P164 (L-250711740P) |
|---|---|---|
| AMEL | X | X |
| CSF1PO | 13 | 13 |
| D13S317 | 12 | 12 |
| D16S539 | 10 | 10 |
| D5S818 | 11 | 11 |
| D7S820 | 12, 13 | 12, 13 |
| TH01 | 7 | 7 |
| TPOX | 8 | 8 |
| vWA | 14, 16 | 14, 16 |
| D18S51 | 13, 15 | 13, 15 |
| D21S11 | 32 | 32 |
| D3S1358 | 18 | 18 |
| D8S1179 | 11 | 11 |
| FGA | 22 | 22 |
| Penta D | 8 | 8 |
| Penta E | -- | -- |
| | Number of shared alleles | 18 |
| | Total number of alleles in the reference profile | 18 |
| | Percent match | 100% |

## COMMENT

The tested cell line has a 100% match with NPC43. Therefore, they are considered to be related from a common ancestry.

**Assay Description & Methodology:**

This test examines 15 human autosomal short tandem repeat (STR) loci and Amelogenin using the PowerPlex® 16HS Kit[1]. Sample DNA is subjected to PCR amplification and is then separated by capillary electrophoresis. The matching criterion is based on an algorithm recommended by the ATCC® Standards Development Organization[2] that compares the number of shared alleles between two cell line samples, expressed as a percentage (Percent match). Cell lines with ≥ 80% match are considered to be related and derived from a common ancestry[2,3]. Cell lines with 55% - 80% match require further analysis for authentication of relatedness. Cell lines with ≤ 55% match are considered unrelated. The test sample is reported as cross-contaminated when the STR profile involves more than the theoretical number of alleles at three or more loci[2].

***Signed out by:***

For and on behalf of Pangenia Lifesciences Ltd.

**References:**

1. PowerPlex® 16HS System Technical Manual, Part#TMD022, Promega Corporation
2. ANSI/ATCC ASN-0002-2011, Designation: ASN-0002 "Authentication of Human Cell Lines: Standardization of STR Profiling"
3. JR Masters *et al.* Proc Natl Acad Sci USA 2001; 98:8012–7
4. Cellosaurus 2019. CLASTR 1.4.4. https://web.expasy.org/cellosaurus-str-search.

PANGENIA
新亞生命科技
A Pangenia Company

PANGENIA LIFESCIENCES LTD
10/F, Stelux House,
No.698 Prince Edward Road East,
San Po Kong, Kowloon, Hong Kong
T +852 3989 6160
F +852 3989 6161
E info@pangenialife.com
pangenialife.com

# Cell Line Authentication Test Report

| | | | |
|---|---|---|---|
| **Investigator Name:** | Jiayan LIU | **Laboratory No.:** | L-250711735P |
| **Institution/Company:** | Department of Microbiology, HKU | **Date of Registration:** | 17 July 2025 |
| **Sample Type:** | DNA | **Date of Issue:** | 26 July 2025 |
| **Sample Name:** | NPC43-C7-M81 P29 | **Report No.:** | 2500000423 |

## TEST RESULTS

| DNA Marker | NPC43 (CVCL_UH64)[4] | NPC43-C7-M81 P29 (L-250711735P) |
|---|---|---|
| AMEL | X | X |
| CSF1PO | 13 | 13 |
| D13S317 | 12 | 12 |
| D16S539 | 10 | 10 |
| D5S818 | 11 | 11 |
| D7S820 | 12, 13 | 12, 13 |
| TH01 | 7 | 7 |
| TPOX | 8 | 8 |
| vWA | 14, 16 | 14, 16 |
| D18S51 | 13, 15 | 13, 15 |
| D21S11 | 32 | 32 |
| D3S1358 | 18 | 18 |
| D8S1179 | 11 | 11 |
| FGA | 22 | 22 |
| Penta D | 8 | 8 |
| Penta E | -- | -- |
| Number of shared alleles | | 18 |
| Total number of alleles in the reference profile | | 18 |
| Percent match | | 100% |

## COMMENT

The tested cell line has a 100% match with NPC43. Therefore, they are considered to be related from a common ancestry.

**Assay Description & Methodology:**

This test examines 15 human autosomal short tandem repeat (STR) loci and Amelogenin using the PowerPlex® 16HS Kit[1]. Sample DNA is subjected to PCR amplification and is then separated by capillary electrophoresis. The matching criterion is based on an algorithm recommended by the ATCC® Standards Development Organization[2] that compares the number of shared alleles between two cell line samples, expressed as a percentage (Percent match). Cell lines with ≥ 80% match are considered to be related and derived from a common ancestry[2,3]. Cell lines with 55% - 80% match require further analysis for authentication of relatedness. Cell lines with ≤ 55% match are considered unrelated. The test sample is reported as cross-contaminated when the STR profile involves more than the theoretical number of alleles at three or more loci[2].

***Signed out by:***

For and on behalf of Pangenia Lifesciences Ltd.

**References:**

1. PowerPlex® 16HS System Technical Manual, Part#TMD022, Promega Corporation.
2. ANSI/ATCC ASN-0002-2011, Designation: ASN-0002 "Authentication of Human Cell Lines: Standardization of STR Profiling"
3. JR Masters *et al.* Proc Natl Acad Sci USA 2001; 98:8012–7.
4. Cellosaurus 2019. CLASTR 1.4.4. https://web.expasy.org/cellosaurus-str-search.

PANGENIA
新亞生命科技
A Pangenia Company

PANGENIA LIFESCIENCES LTD
10/F, Stelux House,
No.698 Prince Edward Road East,
San Po Kong, Kowloon, Hong Kong
T +852 3989 6160
F +852 3989 6161
E info@pangenialife.com
pangenialife.com

# Cell Line Authentication Test Report

| | | | |
|---|---|---|---|
| **Investigator Name:** | Jiayan LIU | **Laboratory No.:** | L-250711731P |
| **Institution/Company:** | Department of Microbiology, HKU | **Date of Registration:** | 17 July 2025 |
| **Sample Type:** | DNA | **Date of Issue:** | 26 July 2025 |
| **Sample Name:** | NP460hTert-EBV P15 | **Report No.:** | 2500000419 |

## TEST RESULTS

| DNA Marker | NP460hTert (CVCL_X205)[4] | NP460hTert-EBV P15 (L-250711731P) |
|---|---|---|
| AMEL | X, Y | X, Y |
| CSF1PO | 13, 14 | 13, 14 |
| D13S317 | 9, 10 | 9, 10 |
| D16S539 | 11, 12 | 11, 12 |
| D5S818 | 11, 12 | 11, 12 |
| D7S820 | 10, 11 | 10, 11 |
| TH01 | 7, 10 | 7, 10 |
| TPOX | 9, 11 | 9, 11 |
| vWA | 17, 19 | 17, 19 |
| D18S51 | 13, 21 | 13, 21 |
| D21S11 | 29, 32.2 | 29, 32.2 |
| D3S1358 | 15, 16 | 15, 16 |
| D8S1179 | 13, 14 | 13, 14 |
| FGA | 22, 25 | 22, 25 |
| Penta D | -- | 11, 12 |
| Penta E | -- | 5, 16 |
| Number of shared alleles | | 28 |
| Total number of alleles in the reference profile | | 28 |
| Percent match | | 100% |

## COMMENT

The tested cell line has a 100% match with NP460hTert. Therefore, they are considered to be related from a common ancestry.

**Assay Description & Methodology:**

This test examines 15 human autosomal short tandem repeat (STR) loci and Amelogenin using the PowerPlex® 16HS Kit[1]. Sample DNA is subjected to PCR amplification and is then separated by capillary electrophoresis. The matching criterion is based on an algorithm recommended by the ATCC® Standards Development Organization[2] that compares the number of shared alleles between two cell line samples, expressed as a percentage (Percent match). Cell lines with ≥ 80% match are considered to be related and derived from a common ancestry[2,3]. Cell lines with 55% - 80% match require further analysis for authentication of relatedness. Cell lines with ≤ 55% match are considered unrelated. The test sample is reported as cross-contaminated when the STR profile involves more than the theoretical number of alleles at three or more loci[2].

***Signed out by:***

For and on behalf of Pangenia Lifesciences Ltd.

Pangenia Life Sciences Limited 新亞生命科技有限公司

**References:**
1. PowerPlex® 16HS System Technical Manual, Part#TMD022, Promega Corporation.
2. ANSI/ATCC ASN-0002-2011, Designation: ASN-0002 "Authentication of Human Cell Lines: Standardization of STR Profiling"
3. JR Masters *et al.* Proc Natl Acad Sci USA 2001; 98:8012–7.
4. Cellosaurus 2019. CLASTR 1.4.4. https://web.expasy.org/cellosaurus-str-search.

PANGENIA
新亞生命科技
A Pangenia Company

PANGENIA LIFESCIENCES LTD
10/F, Stelux House,
No.698 Prince Edward Road East,
San Po Kong, Kowloon, Hong Kong
T +852 3989 6160
F +852 3989 6161
E info@pangenialife.com
pangenialife.com

# Cell Line Authentication Test Report

| | | | |
|---|---|---|---|
| **Investigator Name:** | Jiayan LIU | **Laboratory No.:** | L-250711737P |
| **Institution/Company:** | Department of Microbiology, HKU | **Date of Registration:** | 11 July 2025 |
| **Sample Type:** | DNA | **Date of Issue:** | 15 August 2025 |
| **Sample Name:** | NP361hTert-EBV P17 | **Report No.:** | 2500000425 |

## TEST RESULTS

| DNA Marker | NP361hTert-EBV P17 (L-250711737P) |
|---|---|
| AMEL | X |
| CSF1PO | 10 |
| D13S317 | 8, 10 |
| D16S539 | 10, 13 |
| D5S818 | 12 |
| D7S820 | 11, 12 |
| TH01 | 6, 10 |
| TPOX | 8, 11 |
| vWA | 14, 18 |
| D18S51 | 15, 18 |
| D21S11 | 29, 32.2 |
| D3S1358 | 18, 19 |
| D8S1179 | 10, 14 |
| FGA | 23, 26 |
| Penta D | 9, 12 |
| Penta E | 11, 12 |

## COMMENT

**Assay Description & Methodology**:

This test examines 15 human autosomal short tandem repeat (STR) loci and Amelogenin using the PowerPlex® 16HS Kit[1]. Sample DNA is subjected to PCR amplification and is then separated by capillary electrophoresis. The matching criterion is based on an algorithm recommended by the ATCC® Standards Development Organization[2] that compares the number of shared alleles between two cell line samples, expressed as a percentage (Percent match). Cell lines with ≥ 80% match are considered to be related and derived from a common ancestry[2,3]. Cell lines with 55% - 80% match require further analysis for authentication of relatedness. Cell lines with ≤ 55% match are considered unrelated. The test sample is reported as cross-contaminated when the STR profile involves more than the theoretical number of alleles at three or more loci[2].

***Signed out by:*** 

Pangenia Lifesciences Limited 新亞生命科技有限公司

For and on behalf of Pangenia Lifesciences Ltd.

**References:**

1. PowerPlex® 16HS System Technical Manual, Part#TMD022, Promega Corporation.
2. ANSI/ATCC ASN-0002-2011, Designation: ASN-0002 "Authentication of Human Cell Lines: Standardization of STR Profiling"
3. JR Masters *et al*. Proc Natl Acad Sci USA 2001; 98:8012–7.

PANGENIA
新亞生命科技
A Pangenia Company

PANGENIA LIFESCIENCES LTD
10/F, Stelux House,
No.698 Prince Edward Road East,
San Po Kong, Kowloon, Hong Kong
T +852 3989 6160
F +852 3989 6161
E info@pangenialife.com
pangenialife.com

# Cell Line Authentication Test Report

**Investigator Name:** Jiayan LIU
**Institution/Company:** Department of Microbiology, HKU
**Sample Type:** DNA
**Sample Name:** YCCEL1 P14

**Laboratory No.:** L-250711732P
**Date of Registration:** 11 July 2025
**Date of Issue:** 15 August 2025
**Report No.:** 2500000420

## TEST RESULTS

| DNA Marker | YCCEL1 P14 (L-250711732P) |
|---|---|
| AMEL | X, Y |
| CSF1PO | 11, 12 |
| D13S317 | 8, 13 |
| D16S539 | 9 |
| D5S818 | 9, 11 |
| D7S820 | 10, 11 |
| TH01 | 7 |
| TPOX | 11 |
| vWA | 14, 17 |
| D18S51 | 13 |
| D21S11 | 29.2, 31.2 |
| D3S1358 | 15 |
| D8S1179 | 10, 15 |
| FGA | 18, 23 |
| Penta D | 9 |
| Penta E | 10, 12 |

## COMMENT

**Assay Description & Methodology:**

This test examines 15 human autosomal short tandem repeat (STR) loci and Amelogenin using the PowerPlex® 16HS Kit[1]. Sample DNA is subjected to PCR amplification and is then separated by capillary electrophoresis. The matching criterion is based on an algorithm recommended by the ATCC® Standards Development Organization[2] that compares the number of shared alleles between two cell line samples, expressed as a percentage (Percent match). Cell lines with ≥ 80% match are considered to be related and derived from a common ancestry[2,3]. Cell lines with 55% - 80% match require further analysis for authentication of relatedness. Cell lines with ≤ 55% match are considered unrelated. The test sample is reported as cross-contaminated when the STR profile involves more than the theoretical number of alleles at three or more loci[2].

Pangenia Lifesciences Limited 新亞生命科技有限公司

***Signed out by:***

For and on behalf of Pangenia Lifesciences Ltd.

**References:**

1. PowerPlex® 16HS System Technical Manual, Part#TMD022, Promega Corporation.
2. ANSI/ATCC ASN-0002-2011, Designation: ASN-0002 "Authentication of Human Cell Lines: Standardization of STR Profiling"
3. JR Masters *et al.* Proc Natl Acad Sci USA 2001; 98:8012–7.

PANGENIA
新亞生命科技
A Pangenia Company

PANGENIA LIFESCIENCES LTD
10/F, Stelux House,
No.698 Prince Edward Road East,
San Po Kong, Kowloon, Hong Kong

T +852 3989 6160
F +852 3989 6161
E info@pangenialife.com
pangenialife.com

# Cell Line Authentication Test Report

| | | | |
|---|---|---|---|
| **Investigator Name:** | Jiayan LIU | **Laboratory No.:** | L-250711738P |
| **Institution/Company:** | Department of Microbiology, HKU | **Date of Registration:** | 17 July 2025 |
| **Sample Type:** | DNA | **Date of Issue:** | 26 July 2025 |
| **Sample Name:** | AGS-Bx1 P25 | **Report No.:** | 2500000426 |

## TEST RESULTS

| DNA Marker | AGS (CRL_1739)[4] | AGS-Bx1 P25 (L-250711738P) |
|---|---|---|
| AMEL | X | X |
| CSF1PO | 11, 12 | 11, 12 |
| D13S317 | 12 | 12 |
| D16S539 | 11, 13 | 11, 13 |
| D5S818 | 9, 12 | 9, 12 |
| D7S820 | 10, 11 | 10, 11 |
| TH01 | 6, 7 | 6, 7 |
| TPOX | 11, 12 | 11, 12 |
| vWA | 16, 17 | 16, 17 |
| D18S51 | 13 | 13 |
| D21S11 | 29 | 29 |
| D3S1358 | 16 | 16 |
| D8S1179 | 13 | 13 |
| FGA | 23, 24 | 23, 24 |
| Penta D | -- | 9, 10 |
| Penta E | -- | 13, 16 |
| Number of shared alleles | | 22 |
| Total number of alleles in the reference profile | | 22 |
| Percent match | | 100% |

## COMMENT

The tested cell line has a 100% match with AGS. Therefore, they are considered to be related from a common ancestry.

**Assay Description & Methodology**: This test examines 15 human autosomal short tandem repeat (STR) loci and Amelogenin using the PowerPlex® 16HS Kit[1]. Sample DNA is subjected to PCR amplification and is then separated by capillary electrophoresis. The matching criterion is based on an algorithm recommended by the ATCC® Standards Development Organization[2] that compares the number of shared alleles between two cell line samples, expressed as a percentage (Percent match). Cell lines with ≥ 80% match are considered to be related and derived from a common ancestry[2,3]. Cell lines with 55% - 80% match require further analysis for authentication of relatedness. Cell lines with ≤ 55% match are considered unrelated. The test sample is reported as cross-contaminated when the STR profile involves more than the theoretical number of alleles at three or more loci[2].

***Signed out by:***

For and on behalf of Pangenia Lifesciences Ltd.

**References:**
1. PowerPlex® 16HS System Technical Manual, Part#TMD022, Promega Corporation
2. ANSI/ATCC ASN-0002-2011, Designation: ASN-0002 "Authentication of Human Cell Lines: Standardization of STR Profiling"
3. JR Masters *et al.* Proc Natl Acad Sci USA 2001; 98:8012–7
4. ATCC 2022. STR Profile Database. https://www.atcc.org/search-str-database

PANGENIA
新亞生命科技
A Pangenia Company

PANGENIA LIFESCIENCES LTD
10/F, Stelux House,
No.698 Prince Edward Road East,
San Po Kong, Kowloon, Hong Kong

T +852 3989 6160
F +852 3989 6161
E info@pangenialife.com
pangenialife.com

# Cell Line Authentication Test Report

| | | | |
|---|---|---|---|
| **Investigator Name:** | Jiayan LIU | **Laboratory No.:** | L-250801702P |
| **Institution/Company:** | Department of Microbiology, HKU | **Date of Registration:** | 1 August 2025 |
| **Sample Type:** | DNA | **Date of Issue:** | 7 August 2025 |
| **Sample Name:** | Namalwa | **Report No.:** | 2500000494 |

## TEST RESULTS

| DNA Marker | Namalwa.PNT (CVCL_1841)[4] | Namalwa (L-250801702P) |
|---|---|---|
| AMEL | X | X |
| CSF1PO | 10, 11 | 10, 11 |
| D13S317 | 11, 12 | 12 |
| D16S539 | 9 | 9 |
| D5S818 | 12, 13 | 12, 13 |
| D7S820 | 11 | 11 |
| TH01 | 7, 9.3 | 7, 9.3 |
| TPOX | 6, 11 | 6, 11 |
| vWA | 14 | 14 |
| D18S51 | 15 | 15 |
| D21S11 | 27, 28 | 27, 28 |
| D3S1358 | 16 | 16 |
| D8S1179 | 13, 15 | 13, 15 |
| FGA | 22 | 22 |
| Penta D | 8, 13 | 8, 13 |
| Penta E | 5, 15 | 5, 15 |
| Number of shared alleles | | 24 |
| Total number of alleles in the reference profile | | 25 |
| Percent match | | 96% |

## COMMENT

The tested cell line has a 96% match with Namalwa.PNT. Therefore, they are considered to be related from a common ancestry.

**Assay Description & Methodology**:

This test examines 15 human autosomal short tandem repeat (STR) loci and Amelogenin using the PowerPlex® 16HS Kit[1]. Sample DNA is subjected to PCR amplification and is then separated by capillary electrophoresis. The matching criterion is based on an algorithm recommended by the ATCC® Standards Development Organization[2] that compares the number of shared alleles between two cell line samples, expressed as a percentage (Percent match). Cell lines with ≥ 80% match are considered to be related and derived from a common ancestry[2,3]. Cell lines with 55% - 80% match require further analysis for authentication of relatedness. Cell lines with ≤ 55% match are considered unrelated. The test sample is reported as cross-contaminated when the STR profile involves more than the theoretical number of alleles at three or more loci[2].

***Signed out by:***

For and on behalf of Pangenia Lifesciences Ltd.

**References:**
1. PowerPlex® 16HS System Technical Manual, Part#TMD022, Promega Corporation.
2. ANSI/ATCC ASN-0002-2011, Designation: ASN-0002 "Authentication of Human Cell Lines: Standardization of STR Profiling"
3. JR Masters *et al.* Proc Natl Acad Sci USA 2001; 98:8012–7.
4. Cellosaurus 2019. CLASTR 1.4.4. https://web.expasy.org/cellosaurus-str-search.
